# Resilience as an emergent property of human-infrastructure dynamics: A multi-agent simulation model for characterizing regime shifts and tipping point behaviors in infrastructure systems

**DOI:** 10.1371/journal.pone.0207674

**Published:** 2018-11-21

**Authors:** Kambiz Rasoulkhani, Ali Mostafavi

**Affiliations:** Zachry Department of Civil Engineering, Texas A&M University, College Station, Texas, United States of America; US Army Engineer Research and Development Center, UNITED STATES

## Abstract

The objective of this study is to establish a framework for analyzing infrastructure dynamics affecting the long-term steady state, and hence resilience in civil infrastructure systems. To this end, a multi-agent simulation model was created to capture important phenomena affecting the dynamics of coupled human-infrastructure systems and model the long-term performance regimes of infrastructure. The proposed framework captures the following three factors that shape the dynamics of coupled human-infrastructure systems: (i) engineered physical infrastructure; (ii) human actors; and (iii) chronic and acute stressors. A complex system approach was adopted to examine the long-term resilience of infrastructure based on the understanding of performance regimes, as well as tipping points at which shifts in the performance regime of infrastructure occur under the impact of external stressors and/or change in internal dynamics. The application of the proposed framework is demonstrated in a case of urban water distribution infrastructure using the data from a numerical case study network. The developed multi-agent simulation model was then used in examining the system resilience over a 100-year horizon under stressors such as population change and funding constraints. The results identified the effects of internal dynamics and external stressors on the resilience landscape of infrastructure systems. Furthermore, the results also showed the capability of the framework in capturing and simulating the underlying mechanisms affecting human-infrastructure dynamics, as well as long-term regime shifts and tipping point behaviors. Therefore, the integrated framework proposed in this paper enables building complex system-based theories for a more advanced understanding of civil infrastructure resilience.

## Introduction

Globalization and large-scale urban development in the latter half of the 20^th^ century have precipitated massive demographic shifts in regions across the world, a trend which is expected to continue in the near future. In a bid to cater to these burgeoning developments, the demand for civil infrastructure has grown disproportionately to the supply that is currently made available by civic bodies. For instance, the American Society of Civil Engineers estimates that an additional 3.6 trillion dollars are required, merely to keep up with the increasing rate of demand for infrastructure [1].

An important factor affecting the demand-supply economics of civil infrastructure is the longevity and utility of said infrastructure over a given period of time. External factors such as inclement weather directly affect the performance of physical assets, a statement which is of greater importance today than ever before owing to climate change. In fact, climate change is expected to affect the performance of physical assets, both directly and indirectly [2] as evidenced by the following example. Climate change-induced increase in the number of freeze-thaw cycles directly affects the physical condition of pavements. In addition to this, climate change stimulates changes in the behavior of both users and administrative agencies responsible for the upkeep of pavements, which in turn also affects their physical condition [3]. More generally, civil infrastructure systems such as those catering to transport, power grids, water supply and sewage networks are exposed to various stressors which are broadly classified into two major groups: acute and chronic stressors. Acute stressors are extreme events such as natural and man-made disasters, whereas chronic stressors are gradual, low impact-high probability phenomena which affect infrastructure over a period of time, such as climate change, population growth and decline, and funding limitations, to name a few. Coupled with the aforementioned increase in demand and usage, the impact of these stressors poses a major challenge to scientists and policy-makers concerned with the sustainability of civil infrastructure.

The ability of civil infrastructure to cope with the impact of these stressors is characterized as resilience, which is an emerging topic in the field of infrastructure sustainability. Resilience, in this context, is defined as the capacity of civil infrastructure to experience the impact induced by various types of stressors, while retaining the function to provide services required for the socio-economic development and safety of humans [4]. In essence, it serves to inform scientists and policy-makers about ways to cope with the impact of stressors on infrastructure.

Considering the pivotal role played by civil infrastructure in sustainable socio-economic development as well as in protecting communities, a better understanding of the long-term transformation of infrastructure under external stressors is a critical step towards enhancing the sustainability and resilience of the communities themselves. Over the last decade, literature pertaining to infrastructure resilience [5–8] has centered on examining the physical attributes and topological characteristics that affect the behavior of infrastructure systems under disruption, through the use of an engineering-based approach. The focus of engineering-based approaches is to assess resilience in the presence of acute stressors, while chronic stressors which change the long-term dynamics of infrastructure over a long-time horizon are not fully considered. Engineering resilience approaches primarily focus on event-based analysis of resilience considering system dynamics related to loss of function and short-term recovery of infrastructure for a particular disruptive event. Dynamic behavior of infrastructure is governed by three major factors including (i) physical assets; (ii) human actors; and (iii) chronic and acute stressors, which affect its long-term transformation. The consequence of defining resilience as the final equilibrium state which is a product of the infrastructure’s robustness and resistance to acute stressors, is that the engineering-based approach ignores adaptive behavior seen in its human actors (users, administrative agencies and policy-makers), which is dynamic in nature. However, planning for long-term adaptation to evolving external stressors (e.g., climate change) must contend with potential changes in human decision-maker values and attitudes about the effects of external stressors on hazards and tradeoffs among adaptation options [9]. However, despite the growing literature in the areas of resilience, the characteristics of long-term resilience in civil infrastructure systems are not specified and evaluated. In particular, in the context of chronic stressors (e.g., population change), understanding of long-term resilience characteristics holds the key for robust adaptation planning. For example, performance regime shift is one of the important characteristics of long-term resilience [10]. Evaluation of performance regime shifts in infrastructure systems is very important for long-term adaptation planning and investment decision-making; due to the significant physical and institutional inertia in infrastructure systems, undesirable performance regime shifts are very difficult to reverse [11]. A critical knowledge gap is examining what attributes and relationships in stressor-human-infrastructure nexus would yield long-term resilience that will mitigate the potential impacts of evolving external stressors under different scenarios [12]. Recent developments in the field of complex systems science have addressed some of these concerns [13,14].

Civil infrastructure systems can be considered as complex systems [5] composed of facilities and assets associated with the physical infrastructure, the services they provide to a community, people using these services and the organizations that manage the infrastructure [15]. The resilience of such systems is a function of their internal and external dynamics [13], and their performance depends on the interplay between human actors and the physical assets. Thus, developing a holistic framework for assessing resilience of civil infrastructure requires a better understanding of human actors (users and administrative agencies) and their capacity for adaptive decision-making. Going one step further, under the complex systems approach, resilience of civil infrastructure itself needs to be redefined to account for its dynamic nature. Consequently, resilience may be defined as the ability of a complex system to adapt and transform internal feedback processes, to cope with chronic or “surprise” shocks, recover from internal/external disturbances [4]. That is, the system does not attempt to maintain status quo (equilibrium) through resistance and robustness, rather, the system evolves and improves with non-stationary conditions, through flexibility, diversity and adaptability, which makes resilience as an emergent property of complex systems [4]. An important feature of resilience is that it enables recovery and robustness to unknown and uncertain events [16]. The fact that in complex systems, threats are often impossible to foresee and quantify was one of the main motivations to complement risk-based approaches with resilience analysis [16,17]. Although the proposed approach in this study is not particular threat-diagnostic, it can be used in the presence of “threats”. Essentially, the impact of a threat (i.e., acute stressor) on a system over time would depend on the steady state performance of the system, which is governed by the internal dynamics and chronic stressors.

A complex systems approach holds the key to addressing knowledge gaps in the development of a holistic framework that is capable of conceptualizing and assessing the long-term resilience of civil infrastructure. Under this approach, resilience of civil infrastructure is characterized using three mechanisms: (i) external stressors; (ii) internal dynamics; and (iii) regime shifts. [13] theorized that the resilience of complex systems can be described based on their topology and dynamics. However, the understanding of dynamics that affect infrastructure resilience is rather limited. To address this inadequacy, this paper proposes a framework to capture important phenomena that affect the dynamics, model the long-term performance regimes of infrastructure using a complex systems approach, and analyze the resilience of civil infrastructure in a long-term horizon based on the performance regime shifts and tipping points of external stressors. The components and application of the proposed framework are presented in the context of urban water distribution infrastructure. To this end, a Multi-Agent Simulation (MAS) model was developed to integrate the institutional agencies’ renewal decision-making processes with physical components in order to simulate the transformations, capture the dynamics, and model the performance regimes of water distribution infrastructure system under the impact of external stressors such as population change and funding fluctuations. Using the developed simulation model, various experiments were designed to measure the long-term resilience based on the visual detection of regime shifts and identification of the threshold values (i.e., tipping points) associated with infrastructure performance regimes. That is to say, the long-term resilience of the water distribution infrastructure was evaluated in the presence of stressors using the proposed framework. Accordingly, the effectiveness of different renewal strategies was assessed in light of improving the resiliency of the infrastructure for various scenarios.

## Long-term resilience theoretical framework

**Fig 1:**
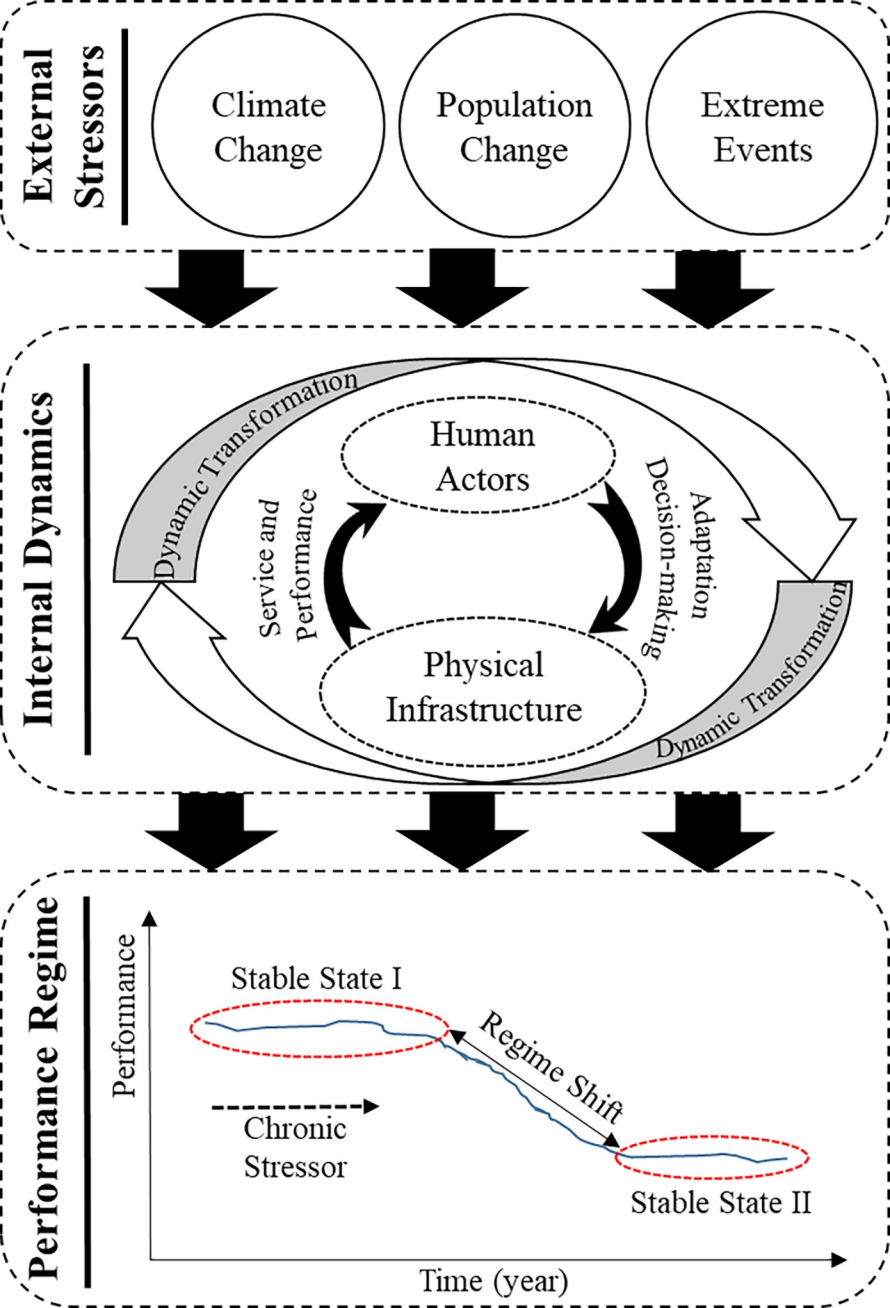
Long-term infrastructure resilience framework using a complex systems approach.

This study utilized a complex system-theoretic approach for the civil infrastructure long-term resilience investment. The literature related to ecological sciences has made significant advancements in adopting a complex systems perspective for understanding the long-term resilience in ecological systems. According to [18–20], resilience in complex ecological systems can be defined based on their ability to cope with the changes in the surrounding environment. In fact, complex systems frequently do not return to their prior state of performance following the impact of stressors. Instead a new equilibrium state is attained, as seen in other fields such as ecology and economics which inspired developments in the engineering resilience field [18]. In the proposed study, grounded theories, measures, and methods related to resilience in complex systems [10,13,18–22] were utilized to build a long-term resilience framework (Fig 1) for civil infrastructure systems under external stressors. The resilience of civil infrastructure system is contingent upon its transformation and adaptation to evolving conditions in the social and environmental sphere [21]. Complex systems approach addresses this need by considering the ability of the system to reach a stable state after a certain threshold (critical or tipping point) has been reached.

### Long-term resilience landscape

Using a complex systems approach, the long-term resilience landscape of civil infrastructure systems can be investigated based on stable states and regimes that determine the performance behavior of the system [10]. Changes in internal dynamics and external disturbances can cause shifts from the current stable state to a new stable state (better or worse) [10]. Accordingly, long-term resilience is defined as the ability of an infrastructure system to maintain its performance regime or transform to a better regime in response to internal and external disturbances. The performance regime of infrastructure system is the state related to the system’s long-term performance in terms of the environmental, social, economic, functionality, and vulnerability parameters, which is maintained by internal dynamics pertaining to stressors-human-infrastructure interactions (Fig 1). The regime shifts occur when a change in the stressor-human-infrastructure interaction triggers a different behavior in the performance of infrastructure system [23,24]. The occurrence of regime shifts is specified based on the detection of steady state transitions in infrastructure system performance behavior [23,24]. Accordingly, the system resilience is evaluated using two methods [20]: (i) examination of steady state transitions in infrastructure behavior over time; and (ii) sensitivity analysis of infrastructure cumulative performance with respect to input parameters (i.e., external stressors).

Based on the existing evidence related to resilience in complex systems [10,13,18–22] this study, as shown in Fig 1, identified three important characteristics related to long-term resilience of civil infrastructure systems: (i) external stressors, (ii) internal dynamics, and (ii) performance regime shifts. The following sub-sections explain the three mechanisms which characterize the resilience of complex infrastructure systems.

#### External stressors

External stressors can be chronic or acute in nature. Chronic stressors are gradual, low impact-high probability events that are caused by changes in the internal feedback processes and the external environment. Acute stressors are abrupt, high impact-low probability events that are mainly caused by extreme events. Both chronic (e.g. accelerated erosion due to climate change) and acute stressors (e.g. flooding) affect the dynamics of the infrastructure and as a consequence, its resilience. For instance, climate change (chronic stressor) is a major driver of changes in the socio-environmental conditions surrounding civil infrastructure [25,26]. Climate change impacts the sustainability and resilience of civil infrastructure in various ways such as (i) changes in temperature and precipitation which affect the erosion of the networks; (ii) population displacement which affects the demand on the networks; (iii) changes in the priorities of agencies which affects allocation of resources; and (iv) increased frequency and magnitude of extreme events (e.g. floods), leading to greater exposure of the networks to risks [27,28]. Hence, changes in decision and physical infrastructure attributes and relationship would change the sensitivity of civil infrastructure system to external stressors. The Threshold values of these attributes at which the sensitivity of infrastructure system to stressors varies can be examined as tipping points. Tipping points occur in complex systems when “a small smooth change made to the parameter values of a system causes a sudden qualitative or topological change in its behavior”. The parameter value at which state transition occurs is referred to as the tipping point or threshold point. Understanding tipping points is essential in describing long-term resilience in complex systems. In the context of civil infrastructure system resilience, regime shifts describe the extent to which the infrastructure system performance regime is sensitive to changes in external stressors magnitudes (e.g., population growth rate). On the other hand, tipping points, explain the critical values related to decision and physical infrastructure attributes (e.g., funding level and network age) that drastically increase or decrease the infrastructure system sensitivity to a certain scenario and stressor magnitude.

#### Internal dynamics

**Fig 2:**
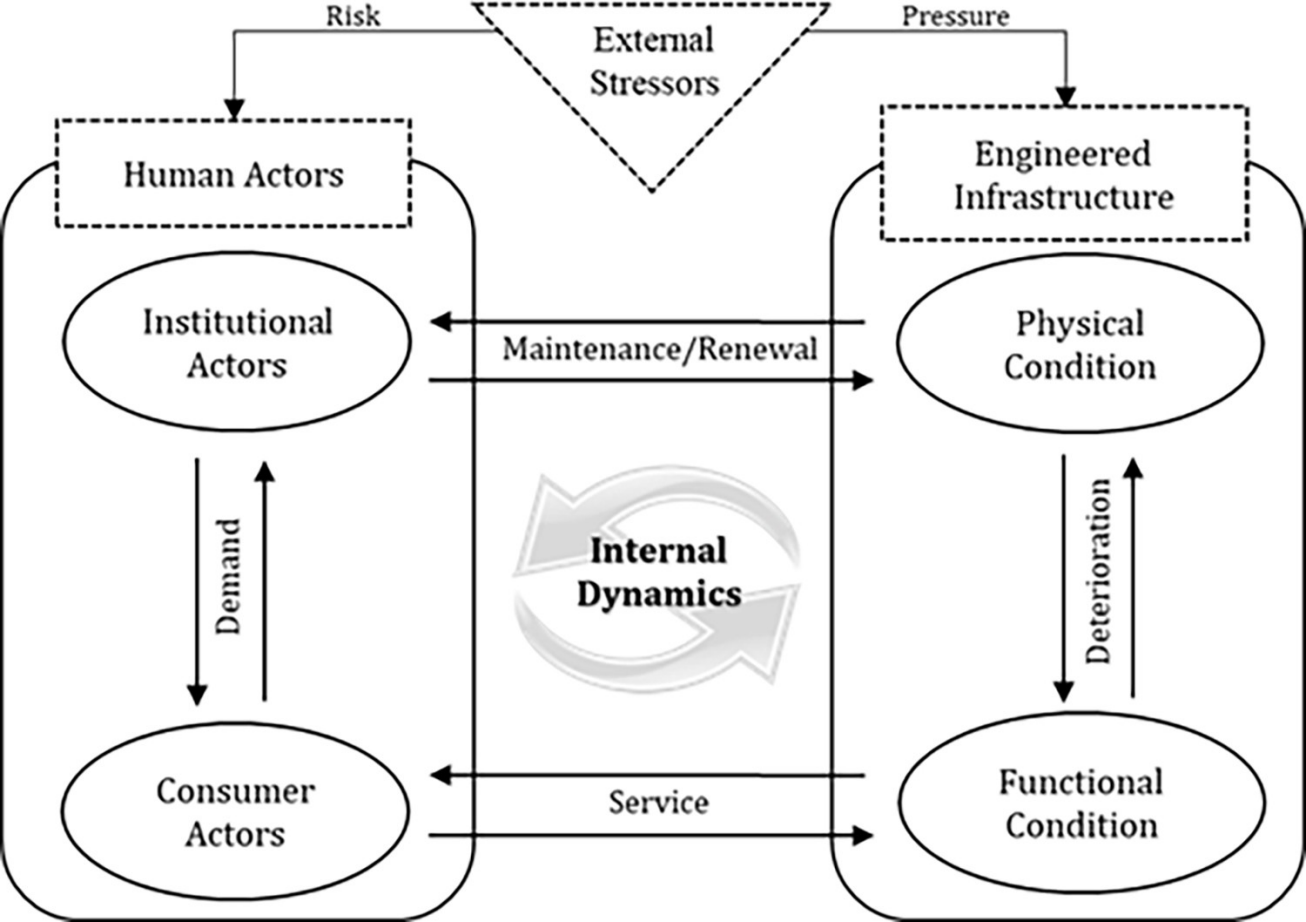
Internal dynamics in complex system of civil infrastructure.

Civil infrastructure as a complex system is composed of various components which are connected by a complex set of direct and indirect interactions and controlled by not one micro-behavior, but by a host of drivers. The observable collective behavior (overall performance) of civil infrastructure emerges from the underlying internal interactions and feedback among the system’s components (internal dynamics), as well as through the impact of external stressors. The internal dynamics of civil infrastructure are determined based on the interaction between human actors (i.e. agencies and users) and physical networks. The dynamics of each component (i.e. human actors and physical networks) are determined by two factors: (i) interaction between the components, which is governed by dynamic rules that impose the activity or decision on the human actors and distribute the condition (performance) to the physical networks; and (ii) global fluctuations in the overall performance of the infrastructure system [29]. Fig 2 depicts the internal dynamics of civil infrastructure through the lens of a complex systems approach. This figure illustrates the couplings between human activities and engineered infrastructure. On the human actor side, two distinct decision-making processes of institutional actors (i.e., infrastructure agencies) can affect the long-term transformation of physical infrastructure. First, actor’s decisions on maintenance, rehabilitation, or reconstruction of assets (referred to as preservation decisions) [22] influence transformation of physical infrastructure by affecting the degradation rates and renewal of assets. Second, the adaptation decisions of actors affect the vulnerability of assets to external stressors. As demonstrated in Fig 2, the decision-making processes of institutional actors affect the expansion, maintenance, and rehabilitation of physical infrastructure. In making their operational and strategic decisions, agencies adopt certain heuristics related to performance (functionality) requirements. These decision-making heuristics are affected by the demand and expectations of the users (consumer actors) as well as by the risks posed by external stressors (acute and chronic stressors). In addition to the decision-making processes of agencies, the functionality of infrastructure networks is affected by attributes (e.g. design, material and age) of physical assets in the network, behavior of its users (e.g. demand) and physical deterioration induced by aging of assets. These internal interactions between human actors and the physical infrastructure, both of which in turn are influenced by external stressors, lead to a certain performance regime for the infrastructure system as a whole. System and policy changes which affect interactions between human actors and physical networks can lead to shifts in the performance regime of the infrastructure. Hence, evaluation of such changes in the performance regime of infrastructure is a key aspect of resilience assessment from a complex systems perspective.

#### Performance regime shifts

The performance regime of infrastructure is defined as the steady state related to the system’s performance, which is maintained by internal dynamics associated with the interactions between human actors and physical infrastructure affected by different stressors. A regime is a characteristic behavior of a system which is maintained by mutually reinforced processes or internal dynamics [23]. A change of regime (regime shift) occurs when a change in an internal process (dynamics) or external disturbance triggers a completely different system performance behavior [19]. Regime shifts are large, abrupt, persistent changes in the performance behavior of a system [30]. The regime shifts occur at tipping points (critical points), where an external stressor interrupts the steady state of the system performance. Evaluation of performance regime shifts is critical for long-term decision-making. Due to significant physical and institutional inertia that is prevalent in civil infrastructure, undesirable performance regime shifts are very difficult to reverse. Identification and adoption of the critical points would facilitate early detection of regime shifts, prior to their occurrence. The occurrence of shifts in the performance regime of an infrastructure system can be detected using different methods such as (i) plotting mean and standard deviation values of the system performance parameters against socio-environmental parameters (e.g. population change, temperature change, and level of funding); and (ii) using time-series data related to different system performance measures in non-parametric methods. To this end, a visual investigation of system performance parameter values under different scenarios can be conducted to identify the non-linear trends and long-term regime shifts.

In the next step of this study, a dynamic (time-dependent) multi-agent simulation model was created and validated to capture and explain the complex dynamics of stressor-human-infrastructure nexus. The elements of the multi-agent model are explained in the following section.

## Multi-agent simulation model

Further to identifying different components of a complex system, capturing mechanisms that dictate dynamic interactions between them is a necessary step towards assessing the long-term resilience performance of the system as a whole. To this end, a Multi-Agent Simulation (MAS) method was adopted in order to capture the coupled human-infrastructure dynamics. MAS enables modeling complex and real-world systems through the adoption of influential concepts such as adaptation, emergence, and self-organization [31]. This method is routinely employed for analyzing problems which require distributed problem-solving capabilities in the absence of a centralized solution [32]. In this method, agents of a complex system are structured such that they are independent entities which function concurrently in the presence of specific relationships that govern the complex system. In MAS, an agent has several essential characteristics: active–initiating actions, reactive–responding to external stimulus, and autonomy [33]. MAS has been shown as an effective simulation approach for analyzing complex processes and interactions in civil infrastructure systems [22,34–36]. Many entities within an infrastructure system (e.g., users, human-decision makers, and physical infrastructure) can be viewed and modeled as an agent. Given that human actors and physical assets of an infrastructure system interact in unique ways at different levels, the MAS method enables capturing such behaviors (i.e., internal dynamics, performance regime, and regime shifts) in a comprehensive framework to assess the long-term resilience of complex systems. Indeed, through developing a MAS model of a water distribution network the proposed framework was tested and used for long-term resilience assessment of this complex infrastructure system. The following section describes the conceptual framework of MAS created for capturing internal dynamics and modeling the impact of external stressors on the performance regime of civil infrastructure, in the context of an urban water distribution system.

### Conceptual model for simulation

**Fig 3:**
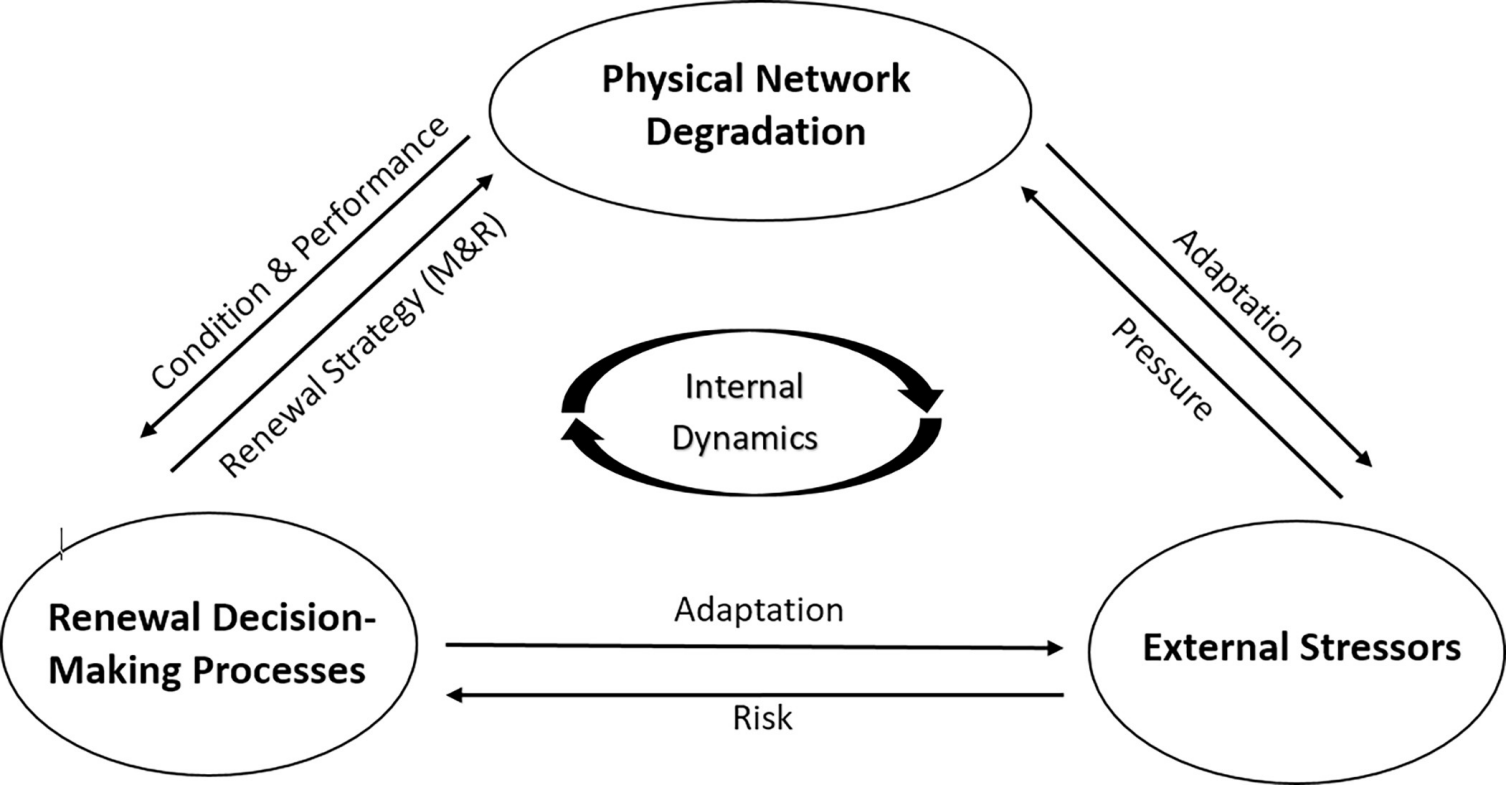
Conceptual modeling framework for water distribution infrastructure systems.

Water distribution infrastructure is a vital part of urban water supply systems, comprising of expensive and often complex physical assets. When thought of as a complex system, it includes physical pipeline networks delivering water to users, the municipality or private company as an institution that manages the infrastructure and the organizations and individuals who consume the water [37]. In water distribution networks, aging pipelines accelerate the effects produced on the infrastructure by external stressors, leading to a faster decay in the performance of this complex system as a whole. The three important drivers of dynamic interactions between agents of a water distribution are shown in Fig 3 below. They include (i) physical degradation of the infrastructure network and its components, which is a function of several factors such as aging, erosion and environmental conditions (ii) renewal decision-making processes and adaptive behavior of institutional actors [38] (iii) external stressors such as population change and fluctuations in funding, which either exacerbate physical degradation of the system or affect the response behavior/decision-making processes of its institutional actors.

#### Physical network degradation

The physical condition of an infrastructure asset denotes its structural capacity and ability to withstand different types of stressors. On the other hand, the functionality of infrastructure indicates its ability to serve its intended function at the desired level of service. The approach to modeling the mechanisms of physical degradation varies for different infrastructure (e.g., roadways vs. water systems) [39]. However, the modeling of infrastructure degradation essentially involves identifying indicators of physical and functional conditions of an asset and then integrating them into a unified performance measure [40]. A unified performance measure quantifies the state of an infrastructure asset at any given time based on variables such as design characteristics of the asset, asset’s age, ambient climate, and service load of the asset in that period [40]. Typical physical attributes of a water distribution network are pipeline age, materials used, length of pipes, fluid pressure and flow rate. The deterioration of physical assets in a network decreases the reliability of the infrastructure and affects the system as a whole in terms of maintenance and operation costs, rehabilitation needs and exposure of actors in the system to risks. Therefore, a complex system model of water distribution infrastructure needs to account for degradation and the effect that it has on service life, condition states, future maintenance and rehabilitation expenses. The physical degradation process of pipework is not clearly understood, with published literature citing several factors such as age, material and network pressure as driving mechanisms, meaning, different functions may be implemented to capture the process [41]. However, several deterioration model studies have correlated age with condition thereby establishing that the process is age-dependent [42].

From a system perspective, age-based deterioration can be used as a criterion to sort various sections (lengths) of piping in the network into discrete age groups based on pre-defined age ranges. As a consequence, aging sections would move from one age group to the next during the process of deterioration. Thus, modeling degradation as a dynamic process would capture long-term transformation effects in the network which would lend greater accuracy to the assessment of the system’s resilience.

#### Renewal decision-making processes

In addition to physical network degradation, human actors (users and administrative agencies) play an important role in the long-term dynamics of water distribution infrastructure. For instance, expectation of and demands placed by users determine the actual quantity of water supplied by the network, while municipalities and private institutions (administrative agencies) which manage the decision-making processes related to renewal and restoration, affect the ability of the network to maintain the required supply. Consider the following scenario which illustrates the interplay of human behavior in the network. The demand placed on the network fluctuates with changes to the user population. These fluctuations drive the price of water, which in turn affects the revenue generated by administrative agencies. Since revenue drives renewal decision processes as well as regular maintenance work, a direct consequence of this interplay is the degradation rate of physical assets in the water distribution network.

In response to the various factors which affect the decision-making process, administrative agencies often identify strategy targets to pursue when maintaining large infrastructure networks. A strategy target may be defined as that indicator of network performance (e.g. average condition and break frequency) which is deemed acceptable by the administrative agency. Thus, any strategy target is a combination of various factors in the behavior spectrum of its human actors. The proposed model captures various behavioral factors affecting the decision-making process such as capital and operational expenses, revenue, water price and capital improvement funds, which are uniquely combined to create three heuristic renewal strategy targets for the network: (i) controlling the average break frequency (ii) controlling average condition of network and (iii) regular renewal based on age of pipes. When the agency adopts a break-control strategy, the motive is to keep the frequency of breaks below a certain target threshold. The annual pipe renewal process would be maintained until the desired average annual break frequency over a five-year horizon is reached. If the target is average condition control, the agency strives to maintain the condition of the network below the required threshold through the renewal process. In either of these strategies, renewal costs exceeding the base allotment are met through the activation of a surplus called the capital improvement fund. This fund is allocated based on a 5-year budget from which 20% can be used annually towards the renewal process if expected costs are exceeded. In the case of an age-based (regular) renewal strategy, the agency performs maintenance work only on sections of the pipeline which are older than 100 years (100 years is the average service life of water pipes as reported by [43]). The capital improvement fund is unavailable when an age-based strategy target is adopted. Therefore, when faced with a deficit, the agency performs renewal based on the amount of available revenue.

It is apparent that renewal decision-making processes of the utility agency and other adaptive actions affect the dynamic variables of the infrastructure, resulting in different performance regimes. Hence, a long-term resilience assessment of the water distribution infrastructure system would be incomplete in their absence.

#### External stressors

The impact of stressors on civil infrastructure systems has been highlighted in the previous sections. The impact of chronic stressors in particular, has greater bearing when the long-term dynamics of a water distribution infrastructure are being investigated. The proposed model framework considers the impact of population change and funding fluctuations as external stressors. Accordingly, different levels of capital improvement fund and various rates of population change are accounted for, to investigate its influence on the dynamics of the infrastructure. Together, water demand fluctuations arising from population change, and availability of funds for renewal, produce critical points which lead to shifts in performance regimes of the water distribution network.

The following section presents the methodology and estimation procedures required to parameterize the MAS model and develop its computational components for a numerical case study of water distribution infrastructure system.

### Computational simulation model

**Fig 4:**
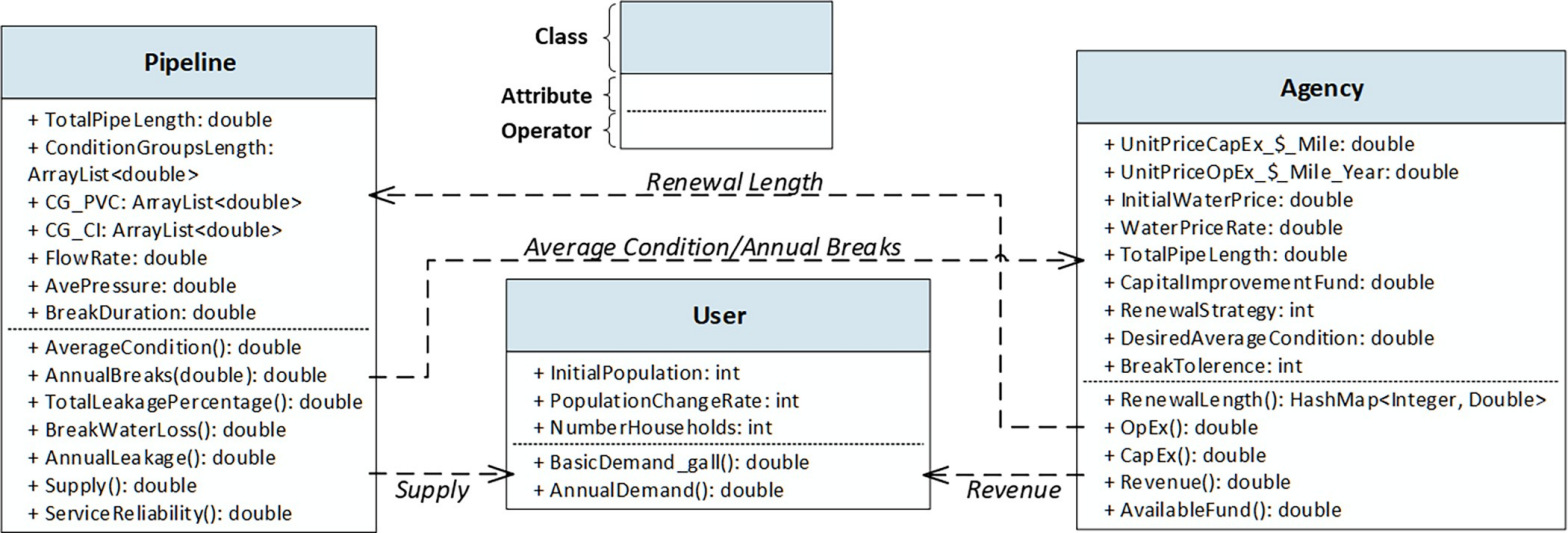
Unified Modeling Language (UML) class diagram of the model.

The MAS model in this study was created based on the conceptual logic and principals representing the real-world behaviors of an urban water distribution infrastructure system. The creation of a computation representation for all the input and output parameters of the conceptual model entails constructing mathematical algorithms to match the conceptual logic representing the behaviors of water distribution infrastructure. The computational representation of the MAS model was developed in an object-oriented programming platform (i.e., AnyLogic 7). It integrates the institutional actors’ renewal decision-making processes with the physical infrastructure performance in order to assess the long-term resilience behavior of water distribution infrastructure system under different stressors (e.g., user population changes) and various scenarios (e.g., renewal strategies). A numerical case of a water distribution network composed of 180 miles of pipes with different materials and age categories was used to create the computational simulation model that captures the dynamics of the system to examine its resilience. The population in the service area of this case is 113,000 (60,000 households). This MAS model of the water distribution infrastructure system includes three classes of agents: water pipeline network, water users, and utility agency, each of which is simulated in the model as an object (i.e., function, variable, or data structure that has memory in the computational model). Fig 4 depicts the Unified Modeling Language (UML) class diagram of the computational MAS model and summarizes the information regarding the attributes and functions implemented.

The relationships among agent classes and their attributes are based on the existing literature and empirical information. Fig 4 shows the attributes, functions, and relationships among different agent classes. The following subsections represent the mathematical implementation for the model agents and their attributes.

#### Pipeline network agent

**Fig 5:**
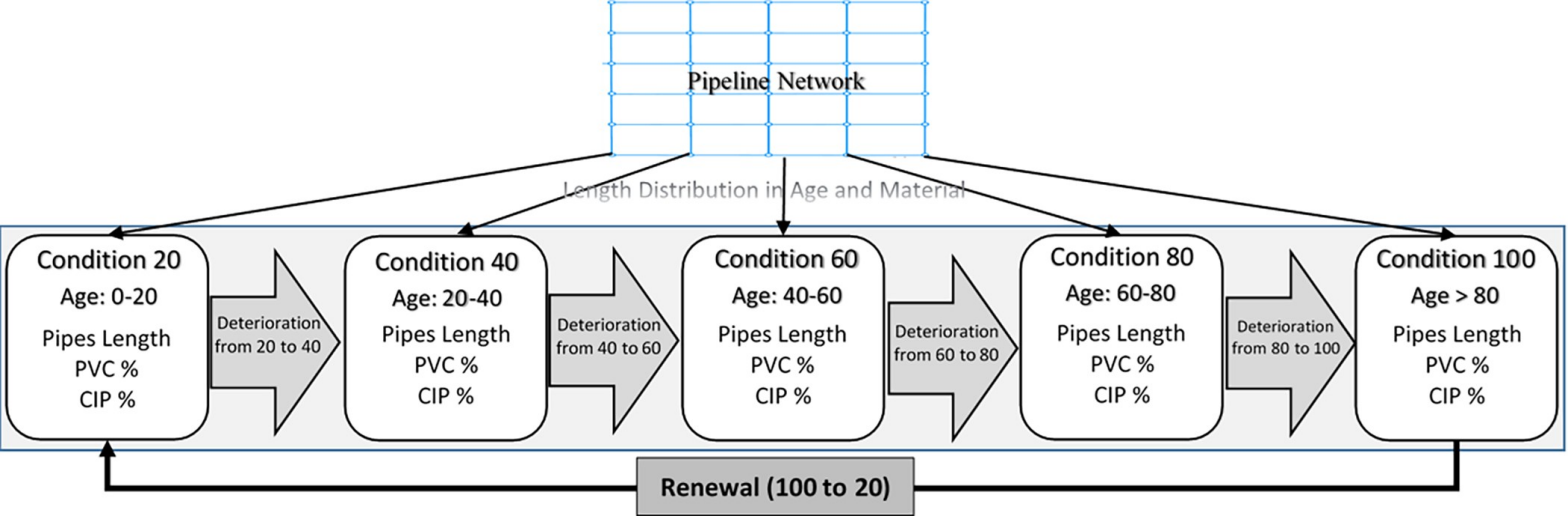
Modeling physical pipeline deterioration process.

The pipeline network agent includes different pipe classes with a defined total length (in miles) divided into 5 condition (age) group states (*i* = 20,40,60,80,100). For each group, which represents a certain condition level, there is a defined material (e.g., percentage of PVC and CIP type). Deterioration of pipes is modeled based on their age. As pipes age, their condition group may change; every year, 5 percent of pipes in each condition group (except condition group 100) moves to the next condition group. Another mechanism that affects the transition of pipes into different condition group states is pipe renewal. Based on the renewal decision-making outcomes of the agency agent (which is explained later), a certain percentage of pipes is renewed and moves from condition group 100 to condition group 20, annually. Fig 5 depicts the modeling deterioration mechanism of pipes in the model.

The aging of the pipes is also reflected in the changes in the percentage of materials for each group. At the beginning of simulation, the model starts with a defined percentage of PVC and CIP pipes for each group. As pipes age, these percentages change (because renewal is by PVC). Based on the portion of pipe length in each condition group, the model computes the network average condition ($\bar{C}$) using Eq 1 [43]:

$$\bar{C}=\frac{\sum_{i}(l_{i}*i)}{L_{N}}\;\left(i=20,40,60,\;80,100\right)$$ (1)

where *l*_*i*_ and *L*_*N*_ are the total length of pipes (mile) in condition group *i* and in the network, respectively. The other attributes of the pipeline network agent include breakage, leakage, and service reliability. Breakage is modeled using a stochastic process that determines the frequency of pipe breaks during one year. The frequency of breaks for each mile of pipes is a function of pipe’s material and age. The model uses a Poisson Process with a mean rate of break (*λ*) (shown in Eq 2) to calculate the annual frequency of pipe breaks per each mile of pipeline. In Eq 2, the Lambda (*λ*) represents the mean rate of break per mile per year [44]:

$$\lambda =N_{0}.e^{g\left(i-10\right)}$$ (2)

where *N*_0_ is the initial number of breaks per mile per year in a new pipe, *g* is the growth rate which is 0.07 for PVC and 0.078 for CIP pipes [45], and *i* is the pipe’s condition group. The total number of breaks in the network per year (*N*_*B*_) would be sum of the breaks in each mile of the entire network pipes. The annual water loss due to network breaks is calculated using Eq 3 [46]:

$$WL_{B}=1440*N_{B}*\bar{F}*d*\sqrt{\bar{P}/70}$$ (3)

where, *WL*_*B*_ is the annual water loss due to network breaks (gallon), *N*_*B*_ is the total number of annual breaks, $\bar{F}$ is the network average flow rate (gallon per minute), *d* is the total duration of disruption due to break (day), and $\bar{P}$ is the network average pressure (psi). $\bar{F},\bar{P}$, and *d* are user-defined input parameters into the model.

Similarly, network leakage is determined as total water volume lost in the network, which is a fraction of annual water demand. Leakage rate (*LR*), which is a unitless variable, depends on network average condition ($\bar{C}$) and is calculated using Eq 4 [47]:

$$LR=0.00075*e^{\bar{C}\left(0.07PVC+0.078CIP\right)}$$ (4)

where *PVC* and *CIP* are the fractions of pipeline network with each material type. Accordingly, the annual water loss due to network leakage is determined by Eq 5:

$$WL_{L}=LR*D$$ (5)

where *WL*_*L*_ and *D* denote the annual water loss due to network leakage (gallon) and the annual water demand (gallon), respectively.

Considering the total water loss in the network due to both breakage and leakage, annual water supply (*S*), which represents the delivered water to users (gallon per year), is calculated using Eq 6:

$$S=D-\left(WL_{B}+WL_{L}\right)$$ (6)

In order to determine whether the supply of water transported by the pipes can meet the given demand, a service reliability (*SR*) parameter is defined. This parameter represents the extent to which the supplied water through the network met the demand. To determine *SR*, in each year (*t*) the cumulative supply is divided by the cumulative demand and this gives the service reliability until that year (*SR*_*t*_) based on Eq 7:

$$SR_{t}=\frac{\sum_{j=1}^{t}S_{j}}{\sum_{j=1}^{t}D_{j}}$$ (7)

where *S*_*j*_ and *D*_*j*_ are delivered water and water demand (gallon) in year j, respectively.

#### Users agent

The agent of users represents population (*P*) and number of households (*N*_*h*_) consuming water from the given pipeline network, which determine the amount of water (gallons) demanded from the network. In each year, water demand (*D*_*t*_) is assumed to be the average indoor water demand (gallon/year) which is calculated using Eq 8 [48]:

$$D_{t}=87.4{\left(\frac{P_{t}}{N_{h}}\right)}^{0.69}*N_{h}*365$$ (8)

Population (*P*_*t*_) is changed in each year based on the user-defined values of growth/decline rate (r) and base population (*P*_0_). Population in each year (t) is computed based on Eq 9:

$$P_{t}=P_{0}*e^{t.r}$$ (9)

#### Utility agency agent

The utility agency agent models the decision-making processes of the agency. The renewal decision-making process is affected by revenue, operational and capital expenditures, and capital improvement fund. The initial water price (*WP*_0_) in the model is user-defined ($/gallon). Water price (*WP*_*t*_) can hike annually over the simulation period with a price hike rate (*w*) based on Eq 10. Accordingly, annual revenue (*Rev*_*t*_) is determined based on that year’s projected water demand (gallon/year) and water price ($/gallon) using Eq 11.

$$WP_{t}=WP_{0}*{\left(1+w\right)}^{t}$$ (10)

$$Rev_{t}=D_{t}*WP_{t}$$ (11)

The annual operational expenditure (*OpEx*), which increases exponentially based on the network average condition ($\bar{C}$), is determined using Eq 12 [47]:

$$OpEx=U_{OpEx}*L_{N}*\left[1+\left(1.4877e^{\left(0.0449\bar{C}\right)}\right)/100\right]$$ (12)

where *U*_*OpEx*_ is the unit cost (dollar per mile) for operating and maintaining the network (which is $0.06 million per mile per year based on [43]).

The renewal rate depends on the availability of funding for the capital expenditures. The available funding for the pipes renewal equals to the annual revenue minus the operational expenditures. The required funding for capital expenditures of renewing pipes is determined using Eq 13.

$$CapEx=U_{CapEx}*RL$$ (13)

where, *U*_*CapEx*_ is the cost of rehabilitation of one mile of pipes with PVC ($1.2 million), and *RL* is the length of pipes (mile) with age 100 or more (5% of pipes in condition group 100). If the available funding for capital expenditures is less than the required capital expenditures, the renewal length would be equal to the available funding divided by the unit cost of rehabilitation.

**Fig 6:**
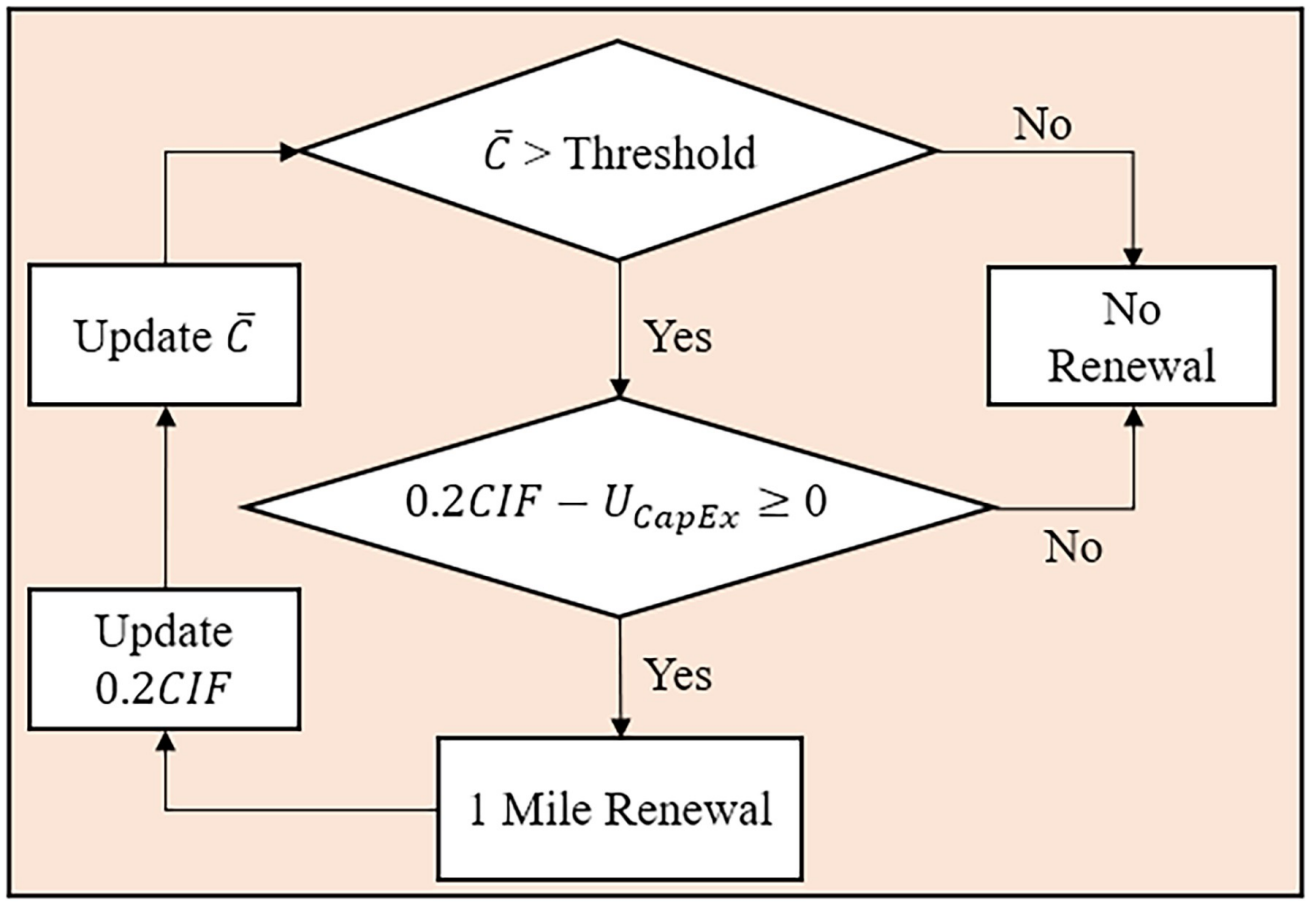
Action chart of renewal strategy for controlling network average condition.

For additional renewal and in order to keep the network performance in terms of break frequency or average condition below the user-defined threshold (i.e., strategy target), the agency would use a capital improvement fund (*CIF*) in addition to the annual revenues. The *CIF* investment is made every five years, and thus the renewal due to *CIF* occurs over five years. Hence, each year, up to twenty percent of *CIF* is invested for renewal. The annual *CIF* allocation would be utilized to implement more pipe renewals in the network after spending the revenue as explained before (i.e., the excessive revenue wouldn’t be used to replenish *CIF*). In the model, *CIF* ($ Million) is a user-defined input parameter. Fig 6 shows the action chart of annual renewal process under the strategy of controlling network average condition. Based on this action chart, the model identifies to what extent (i.e., mile) the renewal process should be conducted every year (as much as the *CIF* allows) in order for the network to reach the strategy target (i.e., desired average condition). That is, similarly, for the strategy of break control, the model determines to what extent the renewal process should be conducted in order for the network to reach the desired number of breaks.

### Verification and validation

Verification and validation techniques focus on verifying the data, rules, logic and computational algorithms [49]. They could be as simple as tracking variables at intermediate levels in simulation and running the simulation with extreme values of constructs [50]. Typically, attempts are made to replicate the outcomes seen in theory, failing which, systematic checks are carried out to identify errors in the code. In this study, a gradual, systemic, and iterative procedure was employed to conduct a thorough verification of the computational model. In addition, the validation of the model was ensured through the use of grounded theories, logic and equations utilized by previous studies in modeling the performance of water distribution networks such as [42,51].

Various internal and external validation techniques (e.g., predictive and face validation) were employed to verify the data, logic, and computational algorithms related to the simulation model. First, the initial conditions and the ranges of the parameters were compared to the existing empirical data to ensure the reliability of the parameters in the model [52]. For example, the parameters related to the physical network, such as pipes’ age, length, and material, were compared to the actual water distribution data related to another network (in Ontario, Canada). Second, the behaviors of model entities (e.g., network average condition) were followed so as to identify unusual model behaviors. Whenever an unusual behavior was observed, the model logic was checked to ensure that the behavior was not due to unreasonable assumptions or imperfect logic. Third, extreme value analyses were performed, where the model was run at different extreme conditions (for each component) to observe its response and verify its functionality under these scenarios. Fourth, several random replications (more than one thousand runs) of the model were compared to check for the consistency of the results [53]. Fifth, predictive validation of the model was conducted. In predictive validation, the model is used to predict the system’s behavior, and then the model’s forecast is compared with actual system’s behavior obtained, for example, from data related to behaviors of an operational system [54]. To conduct the predictive validation, the outputs related to each model specification were compared to the existing data related to the water distribution networks in Fort Collins, CO. For example, the simulated annual leakage and breakage rates of the network were compared to the real values based on historical data. Accordingly, most of the observed errors pertained to incorrect implementation of the algorithm (the algorithm itself proved robust) in the code and were rectified easily. Finally, a face validation was pursued by research team through examining the simulated behaviors of the model (output results) to ensure that they are reasonable for a real case. Following this, the quality of the model components was ensured for completeness, coherence, consistency and correctness (4Cs) [38] based on the performance of the model outputs.

## Simulation experiments

After using different internal verification and external validation techniques to ensure the model quality, the simulation model was used for building various experiments based on all possible scenarios. Various simulation experiments were conducted through the change of model input parameter values and logics in the computational model. The possible scenarios were established based on different combinations of the input variables (parameters) in the model, shown in Table 1. The combinations of these scenarios reflect changes in population growth rate, capital improvement fund level, water price hike, renewal strategy, and strategy target. Since network break frequency is modeled as a stochastic Poisson process, one thousand runs of Monte-Carlo experiments were conducted to determine the mean value of the model output parameters (e.g., service reliability) under each specific scenario.

**Table 1:** Model input variables.

Input Variable	Variable Value(s)
Base Population	113,000
Number of Households	60,000
Population Change Rate (%)	-2, -1.5, -1, -0.5, 0, 0.5, 1, 1.5, 2
Pipeline Renewal Strategy	1. Regular Renewal 2. Controlling Network Average Condition at 40, 45, and 50 3. Controlling Average Break Frequency at 20, and 25
Initial Water Price ($/gallon)	0.005
Water Price Hike Rate (%)	0, 4, 8
Capital Improvement Fund ($ Million)	0, 10, 15, 20, 25, 30

Two sets of simulation experiments were conducted to evaluate the long-term resilience of the water distribution infrastructure system. First, the impacts of decision and physical infrastructure attributes were examined in order to explore the significant attributes and their critical threshold value that could lead to the occurrence of tipping point behaviors. To this end, time-series results related to the network average condition were visualized to investigate the existence of non-linear increase or decline trends in the simulated long-term performance measure. Accordingly, the threshold values related to decision and infrastructure attributes (e.g., level of capital improvement funding and desired network average condition), at which the steady state of infrastructure performance is disrupted were examined as tipping points. The results related to regime shifts and tipping points were used to evaluate the long-term resilience of the case study network under different adaptation strategy scenarios (i.e., funding allocation and renewal strategies). Second, the impacts of population changes and funding fluctuations on the network performance parameters and its ultimate effects on network service reliability over the analysis period (i.e., 100 years) were investigated. To this end, the mean values of the infrastructure service reliability were plotted against different values of population change rate and capital improvement funding level to visually examine the occurrence of shifts in the service reliability performance of the infrastructure system. Accordingly, the occurrence of regime shifts and tipping points were detected and used to analyze the sensitivity of long-term infrastructure system performance to external stressors.

## Results

**Fig 7:**
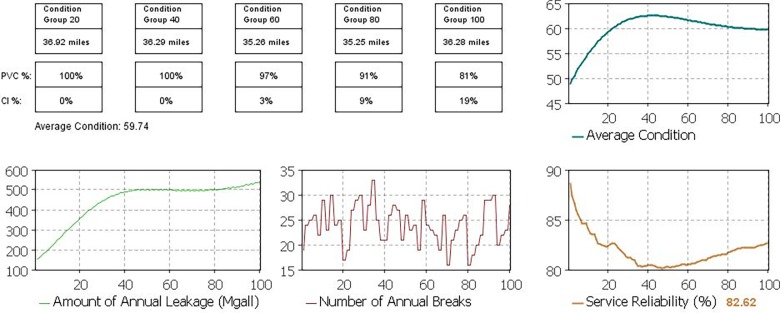
Simulation model output dashboard: Visualized long-term performance regimes.

The MAS model of water distribution infrastructure system was used to extract, animate and visualize the long-term regimes of different performance and condition measures in the case study water distribution network. For instance, the simulation model was run for a scenario of 1% population growth, 4% water price hike, and regular renewal strategy. Fig 7 shows a screenshot of the graphical output dashboard of the simulation model under this scenario. As can be seen in Fig 7, this dashboard displays the 100-year regimes of the network average condition (age), the network leakage, the network annual breaks, and the system service reliability. Also, age-material distribution of the pipes of the network in each year is observable in this dashboard.

These visualized long-term performance regimes of the water distribution infrastructure are utilized to detect the occurrence of regime shifts, visually (as discussed before, a visual investigation of system performance parameter values under different scenarios could be used to identify long-term performance regime shifts); and accordingly, the long-term resilience of the system is evaluated. Thanks to the analysis results, two sets of theoretical constructs related to complex systems approach to infrastructure resilience were examined: (i) the performance regime of infrastructure is shaped by its internal dynamics; and (ii) the effective performance of infrastructure is sensitive to external chronic stressors.

### Internal dynamics and performance regime

The simulation model was used to examine how infrastructure dynamics shape performance regimes. To this end, the effect of renewal strategies (as an element affecting infrastructure dynamics) on the performance regime of the network was evaluated. Two renewal strategies (i.e., condition and break control) were considered. For each renewal strategy, different required performance targets and capital improvement funding levels were assessed. In total, 25 scenarios were simulated, where each scenario represents a unique state of infrastructure dynamics in the case study. In this analysis, resilience is determined based on detection of regime shifts in the stable state of performance in the system. Different performance measures could be used for assessing possible regime shifts. In this set of analysis, the network average condition was selected among the network performance measures for two reasons: first, the network average condition collectively represents the physical state of the pipes in the network; second, most of the network performance measures such as leakage, breakage, and service reliability, either directly or indirectly, are affected by the network average condition. Accordingly, resilience of the system is analyzed based on regime shifts in the long-term average condition of the network.

**Fig 8:**
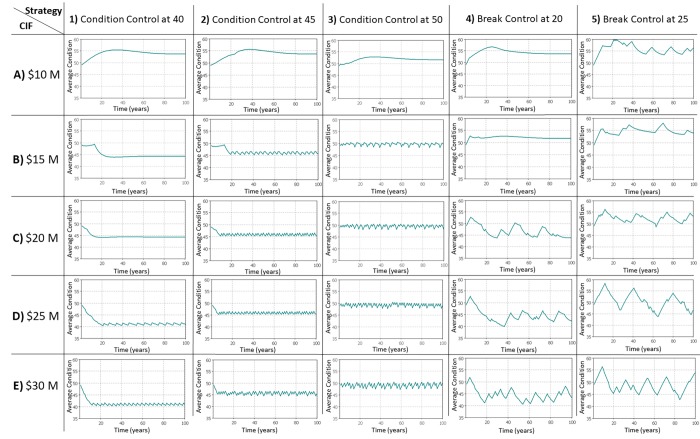
Modeling regimes of network average condition under various scenarios.

Fig 8 shows network average conditions plotted over a 100-year horizon under 1% population growth rate for all the scenarios. In Fig 8, each column represents a certain renewal strategy and each row represents a certain level of capital improvement fund (*CIF*). As can see in Fig 8, the state of infrastructure dynamics in each scenario leads to a certain regime in the performance of the network. For example, under scenarios A1-A4, the behavior (regime) of the network performance follows a similar trend; however, under scenario A5, the dynamics of the system changes (because of change in renewal strategy target), and hence the infrastructure performance changes.

This change in the performance regime of infrastructure is a significant phenomenon and can be characterized as a critical point or tipping point. Critical or tipping points represent values related to the internal dynamics of infrastructure or external stressor, in which a small change in the parameter value leads to a significant change in the performance of infrastructure. For example, Fig 8 shows that under a fix capital improvement funding (e.g., $20 Million), a change in the renewal strategy from condition control to break control will lead to a shift in the performance regime of the network (compare scenarios C1-C3 with C4-C5). Under each renewal strategy, there is a certain value of capital improvement funding that leads the infrastructure system to a resilient behavior (i.e., the network meets the desired target with a stable regime). This value, accordingly, is specified as a tipping or critical point. For instance, under the renewal strategies of controlling average condition at 40, 45, and 50, any capital improvement funding below $25 million, $20 million, and $15 million, respectively, wouldn’t lead the system to a resilient behavior (compare D1 with C1, C2 with B2, and B3 with A3). These results show that internal dynamics shape the performance regime of infrastructure and how the changes in these internal dynamics affect the long-term performance regimes and cause the regime shifts.

### Impact of chronic stressors

**Fig 9:**
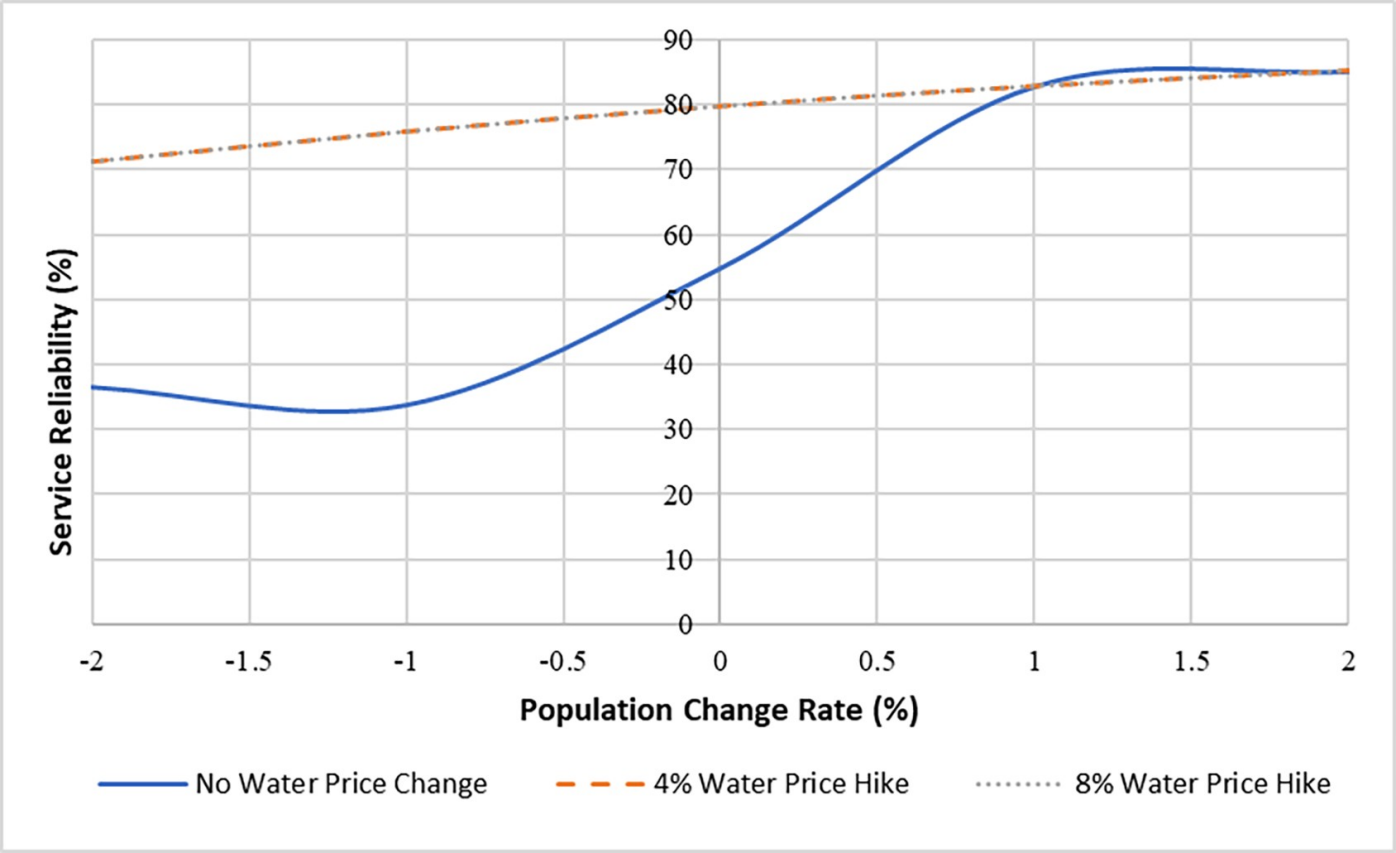
Impact of population change (chronic stressor) on system service reliability.

**Fig 10:**
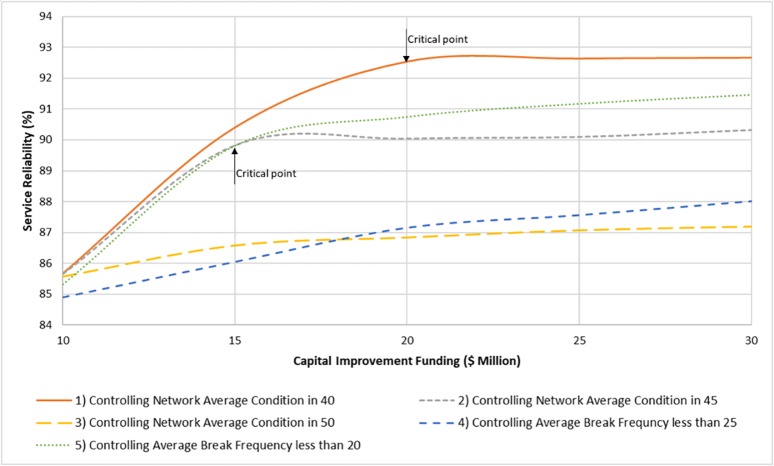
Impact of funding fluctuations on system service reliability.

The simulation model was also used to examine the impact of external chronic stressors on the performance regime of the case study infrastructure. To this end, the effect of population changes and funding fluctuations were considered. Various scenarios of population change rates and levels of capital improvement fund were defined; and then for each scenario, one thousand runs of Monte-Carlo experiments were implemented to determine the mean value of the water distribution infrastructure’s service reliability. The service reliablity indicator measures how reliable the network is in successfully delivering the demanded water to users. Figs 9 and 10 show the long-term service reliability of the network over 100 years (i.e., *t* = 100 in Eq 7) under the defined scenarios. Fig 9 shows that, under regular renewal strategy, without any changes in the water price, the service reliability of system drops significantly when the system faces a decline in the population of the service area. However, if the water price grows 4% or greater annually, the sensitivity of the network performance to population change decreases. Based on the results, implementing a plan of 4% water price hike annually, will lead this water distribution infrastructure system to a resilient behavior against the population decline over the 100-year horizon. However, a 4% annual price hike might be greater than the inflation rate or the average rate of household income increase, which makes the implementation of this plan impossible [55].

Since for two other renewal strategies the extent of renewal processes prominently depends on the capital improvement fund, the impact of this external stressor (funding fluctuations) on the system effective performance was examined under different renewal scenarios. Fig 10 shows the sensitivity of five different scenarios of renewal strategy to the level of capital improvement fund. It is seen that, renewal scenario of 3 and 4 have a roughly steady-state trend within different levels of the capital improvement fund. However, for three other renewal scenarios, there are two different phases over the trend of service reliability, which indicates a critical point for capital funding level. Critical point means the threshold level of funding, around which the service reliability of system significantly changes from the previous phase. As shown in Fig 10, for the renewal scenarios 1 and 2, $20 Million and $15 Million are the critical points of capital improvement fund, respectively. Hence, it seems for the case study water distribution network, if strategically implemented, renewing the pipes at a rate to keep the network average condition at 40 will lead the infrastructure system towards a more resilient behavior over the long-term horizon. These example results show the sensitivity of infrastructure system performance to external stressors and to what extent they affect the effective performance indicator of infrastructure system.

## Concluding remarks

This study was conducted with the objective of capturing internal dynamic behaviors which influence the long-term resilience of civil infrastructure systems in the presence of external stressors. The proposed framework characterizes the object of study (a water distribution network) as a complex system and offers a multi-agent simulation (MAS) model to quantify its components, dynamic processes, and external stressors acting on it. The output of the model depicts the performance regime of the system over an extended horizon which enables the detection of regime shifts to evaluate the long-term resilience.

Different performance regimes were observed in response to changes in the renewal decision-making process of the administrative agency, which is an expected outcome owing to the fact that availability of funding is a function of revenue, which in turn depends on the demand generated by the user population. The model also identified tipping points in the performance regime when specific renewal strategy targets were adopted by the administrative agency. For instance, when a renewal strategy of average condition controlling at 45 was adopted in conjunction with a capital improvement fund of $15 Million, the infrastructure satisfied the target by exhibiting long-term performance. Any decrease to the fund however, caused a regime shift if the agency continued to maintain the same strategy target. These results show that the performance regime of an infrastructure system is shaped by its internal dynamics, which reinforces the premise that changes in internal dynamics would lead to regime shifts in long-term behavior of the system. The model also adequately captured the long-term response of infrastructure when subjected to external stressors. For instance, a positive correlation between decline in user population and the long-term service reliability of the infrastructure was observed. The model showed that a price hike of 4% annually would maintain the service reliability of the case study water distribution infrastructure system in a stable state, despite changes to its user population.

## Contribution and significance

The contributions of this study are threefold: theoretical, computational, and practical contributions. From theoretical perspective, this study proposed a complex system-based framework for infrastructure resilience assessment through a better understanding of internal dynamics and tipping point behaviors. Accordingly, it proves that (i) the performance regimes of infrastructure are shaped by their internal dynamics and (ii) chronic stressors affect the effective performance of infrastructure system. In terms of computational contribution, this study developed a MAS model of a water distribution network that adequately captures and quantifies the dynamic behaviors and performance regimes of the infrastructure system in the presence of external stressors. Practically speaking, through the quantifying the impacts of external stressors, internal dynamics and performance regime shifts, researchers can predict the resilience of civil infrastructure systems with greater accuracy. This in turn would help decision-makers formulate policies (e.g., renewal strategy) that enhance the sustainability and resilience of these systems.

## Future studies

The simulation model presented in this study doesn’t include all the dynamic mechanisms affecting the long-term resilience of a coupled human-infrastructure system. Depending on the objective of a study, additional dynamics can be captured and modeled using the proposed framework. For example, in the analysis shown in this paper, the influence of user behaviors was not within the study objectives. Hence, in this model, the influence of consumer actors was modeled exogenously with a prescribed rate of population growth and water demand. Future studies can examine the dynamics of consumer behaviors in evaluating the long-term resilience of infrastructure systems by evaluating individual consumer’s response to water price [55] and other incentives (such as rebate) [36].

## Supporting information

S1 File Source code of agent classes. (DOCX)

S1 Fig Input interface of the simulation model. (TIF)

S2 Fig Output dashboard of the simulation model. (TIF)
