# Supplementary material for: Resilience as an emergent property of human-infrastructure dynamics: A multi-agent simulation model for characterizing regime shifts and tipping point behaviors in infrastructure systems
Source: PLoS One. 2018 Nov 21;13(11):e0207674. doi: 10.1371/journal.pone.0207674 (PMC6248985; doi:10.1371/journal.pone.0207674)
Supplement: S1 File — (DOCX) [file pone.0207674.s001.docx]

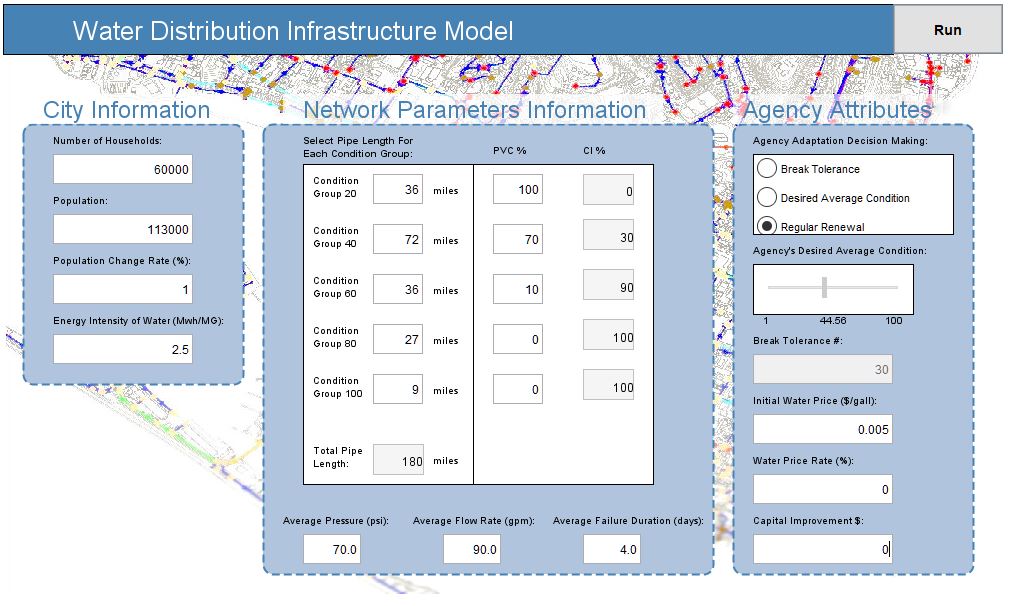


**S1 Fig.** **Input Interface of the Simulation Model**


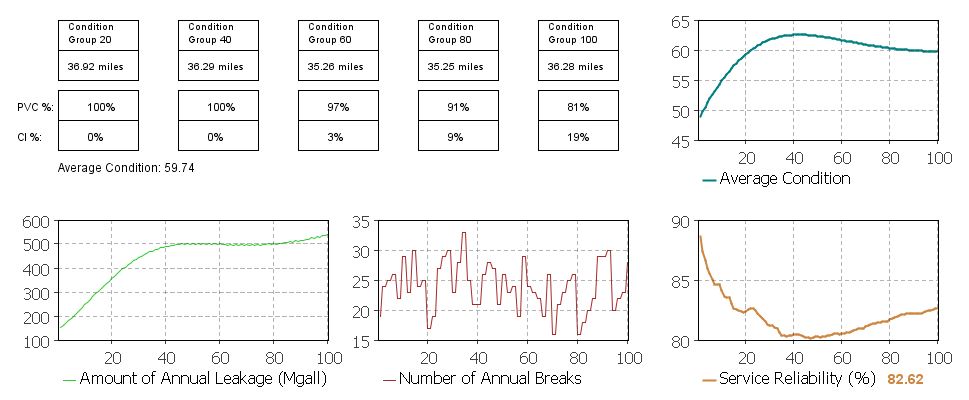


**S2 Fig.** **Output Dashboard of the Simulation Model**

The simulation model has been uploaded in an open repository (AnyLogic Cloud), where it can be implemented online. Please use the link below to access to the model:

<https://cloud.anylogic.com/model/68cb421d-129f-4531-9fc4-a78e3bba12ef?mode=SETTINGS>

**Source Code**

The source code of the AnyLogic model was extracted and provided as following:

package waterinfrastructure;

public class Agency extends Agent

{

// Parameters

public

double TotalPipeLength;

/**

* Returns default value for parameter <code>TotalPipeLength</code>.

* <i>This method should not be called by user</i>

*/

@AnyLogicInternalCodegenAPI

public double _TotalPipeLength_DefaultValue_xjal() {

final Agency self = this;

return 0.0;

}

public void set_TotalPipeLength( double TotalPipeLength ) {

if (TotalPipeLength == this.TotalPipeLength) {

return;

}

double _oldValue_xjal = this.TotalPipeLength;

this.TotalPipeLength = TotalPipeLength;

onChange_TotalPipeLength_xjal( _oldValue_xjal );

onChange();

}

/**

* Calls "On change" action for parameter TotalPipeLength.<br>

* Note that 'oldValue' in that action will be unavailable if this method is called by user

* (current parameter value will be passed as 'oldValue').<br>

* Please call <code>set_TotalPipeLength()</code> method instead.

*/

protected void onChange_TotalPipeLength() {

onChange_TotalPipeLength_xjal( TotalPipeLength );

}

@AnyLogicInternalCodegenAPI

protected void onChange_TotalPipeLength_xjal( double oldValue ) {

}

/**

* Value may be changed later

*/

public

double UnitPriceCapEx_$_Mile;

/**

* Returns default value for parameter <code>UnitPriceCapEx_$_Mile</code>.

* <i>This method should not be called by user</i>

*/

@AnyLogicInternalCodegenAPI

public double _UnitPriceCapEx_$_Mile_DefaultValue_xjal() {

final Agency self = this;

return

1200000

;

}

public void set_UnitPriceCapEx_$_Mile( double UnitPriceCapEx_$_Mile ) {

if (UnitPriceCapEx_$_Mile == this.UnitPriceCapEx_$_Mile) {

return;

}

double _oldValue_xjal = this.UnitPriceCapEx_$_Mile;

this.UnitPriceCapEx_$_Mile = UnitPriceCapEx_$_Mile;

onChange_UnitPriceCapEx_$_Mile_xjal( _oldValue_xjal );

onChange();

}

/**

* Calls "On change" action for parameter UnitPriceCapEx_$_Mile.<br>

* Note that 'oldValue' in that action will be unavailable if this method is called by user

* (current parameter value will be passed as 'oldValue').<br>

* Please call <code>set_UnitPriceCapEx_$_Mile()</code> method instead.

*/

protected void onChange_UnitPriceCapEx_$_Mile() {

onChange_UnitPriceCapEx_$_Mile_xjal( UnitPriceCapEx_$_Mile );

}

@AnyLogicInternalCodegenAPI

protected void onChange_UnitPriceCapEx_$_Mile_xjal( double oldValue ) {

}

public

double InputUserFees;

/**

* Returns default value for parameter <code>InputUserFees</code>.

* <i>This method should not be called by user</i>

*/

@AnyLogicInternalCodegenAPI

public double _InputUserFees_DefaultValue_xjal() {

final Agency self = this;

return 0.0;

}

public void set_InputUserFees( double InputUserFees ) {

if (InputUserFees == this.InputUserFees) {

return;

}

double _oldValue_xjal = this.InputUserFees;

this.InputUserFees = InputUserFees;

onChange_InputUserFees_xjal( _oldValue_xjal );

onChange();

}

/**

* Calls "On change" action for parameter InputUserFees.<br>

* Note that 'oldValue' in that action will be unavailable if this method is called by user

* (current parameter value will be passed as 'oldValue').<br>

* Please call <code>set_InputUserFees()</code> method instead.

*/

protected void onChange_InputUserFees() {

onChange_InputUserFees_xjal( InputUserFees );

}

@AnyLogicInternalCodegenAPI

protected void onChange_InputUserFees_xjal( double oldValue ) {

}

public

double CapitalImprovement;

/**

* Returns default value for parameter <code>CapitalImprovement</code>.

* <i>This method should not be called by user</i>

*/

@AnyLogicInternalCodegenAPI

public double _CapitalImprovement_DefaultValue_xjal() {

final Agency self = this;

return 0.0;

}

public void set_CapitalImprovement( double CapitalImprovement ) {

if (CapitalImprovement == this.CapitalImprovement) {

return;

}

double _oldValue_xjal = this.CapitalImprovement;

this.CapitalImprovement = CapitalImprovement;

onChange_CapitalImprovement_xjal( _oldValue_xjal );

onChange();

}

/**

* Calls "On change" action for parameter CapitalImprovement.<br>

* Note that 'oldValue' in that action will be unavailable if this method is called by user

* (current parameter value will be passed as 'oldValue').<br>

* Please call <code>set_CapitalImprovement()</code> method instead.

*/

protected void onChange_CapitalImprovement() {

onChange_CapitalImprovement_xjal( CapitalImprovement );

}

@AnyLogicInternalCodegenAPI

protected void onChange_CapitalImprovement_xjal( double oldValue ) {

}

@Override

public void setParametersToDefaultValues() {

super.setParametersToDefaultValues();

TotalPipeLength = _TotalPipeLength_DefaultValue_xjal();

UnitPriceCapEx_$_Mile = _UnitPriceCapEx_$_Mile_DefaultValue_xjal();

InputUserFees = _InputUserFees_DefaultValue_xjal();

CapitalImprovement = _CapitalImprovement_DefaultValue_xjal();

}

@Override

public boolean setParameter(String _name_xjal, Object _value_xjal, boolean _callOnChange_xjal) {

switch ( _name_xjal ) {

case "TotalPipeLength":

if ( _callOnChange_xjal ) {

set_TotalPipeLength( ((Number) _value_xjal).doubleValue() );

} else {

TotalPipeLength = ((Number) _value_xjal).doubleValue();

}

return true;

case "UnitPriceCapEx_$_Mile":

if ( _callOnChange_xjal ) {

set_UnitPriceCapEx_$_Mile( ((Number) _value_xjal).doubleValue() );

} else {

UnitPriceCapEx_$_Mile = ((Number) _value_xjal).doubleValue();

}

return true;

case "InputUserFees":

if ( _callOnChange_xjal ) {

set_InputUserFees( ((Number) _value_xjal).doubleValue() );

} else {

InputUserFees = ((Number) _value_xjal).doubleValue();

}

return true;

case "CapitalImprovement":

if ( _callOnChange_xjal ) {

set_CapitalImprovement( ((Number) _value_xjal).doubleValue() );

} else {

CapitalImprovement = ((Number) _value_xjal).doubleValue();

}

return true;

default:

return super.setParameter( _name_xjal, _value_xjal, _callOnChange_xjal );

}

}

@Override

public <T> T getParameter(String _name_xjal) {

Object _result_xjal;

switch ( _name_xjal ) {

case "TotalPipeLength": _result_xjal = TotalPipeLength; break;

case "UnitPriceCapEx_$_Mile": _result_xjal = UnitPriceCapEx_$_Mile; break;

case "InputUserFees": _result_xjal = InputUserFees; break;

case "CapitalImprovement": _result_xjal = CapitalImprovement; break;

default: _result_xjal = super.getParameter( _name_xjal ); break;

}

return (T) _result_xjal;

}

@AnyLogicInternalCodegenAPI

private static String[] _parameterNames_xjal;

@Override

public String[] getParameterNames() {

String[] result = _parameterNames_xjal;

if (result == null) {

List<String> list = new ArrayList<>( Arrays.asList( super.getParameterNames() ) );

list.add( "TotalPipeLength" );

list.add( "UnitPriceCapEx_$_Mile" );

list.add( "InputUserFees" );

list.add( "CapitalImprovement" );

result = list.toArray( new String[ list.size() ] );

_parameterNames_xjal = result;

}

return result;

}

// Plain Variables

/**

* dollar per gallon

*/

public

double

UserFees;

public

double

TotalExToDate;

public

double

UserFeeHike;

public

double

UserFeeDecline;

public

double

UnitPriceOpEx_$_Mile_Year;

public

double

YearBudget;

// Dynamic (Flow/Auxiliary/Stock) Variables

public double AverageCondition;

public double RequiredCapEx;

public double Revenue;

public double AvailableCapEx;

public double AnnualDemand;

public double AgencyWaterSupply;

public double AgencyWaterDemand;

@AnyLogicInternalCodegenAPI

public void assignInitialConditions_xjal() {

super.assignInitialConditions_xjal();

_assign_AnnualDemand_Formula_xjal();

_assign_Revenue_Formula_xjal();

_assign_AverageCondition_Formula_xjal();

_assign_RequiredCapEx_Formula_xjal();

_assign_AvailableCapEx_Formula_xjal();

_assign_AgencyWaterSupply_Formula_xjal();

_assign_AgencyWaterDemand_Formula_xjal();

}

@AnyLogicInternalCodegenAPI

public void setupInitialConditions_xjal(Class<?> callerClass) {

if (callerClass != Agency.class) {

return;

}

if (getInitialAlgebraicFlatEquationsCount_xjal() > 0) {

SDIntegrationManager integrationManagerForInitialConditions = new SDIntegrationManager( 0, getInitialAlgebraicFlatEquationsCount_xjal(), getInitialFormulaFlatEquationsCount_xjal() );

integrationManagerForInitialConditions.doStep( this, 0, 0.1, true );

} else {

assignInitialConditions_xjal();

}

}

@AnyLogicInternalCodegenAPI

public void _assign_AnnualDemand_Formula_xjal() {

AnnualDemand =

main.user.getAnnualDemand()

;

}

@AnyLogicInternalCodegenAPI

public void _assign_Revenue_Formula_xjal() {

Revenue =

main.user.WaterPrice() * AnnualDemand

;

}

@AnyLogicInternalCodegenAPI

public void _assign_AverageCondition_Formula_xjal() {

AverageCondition =

main.pipe.AverageCondition()

;

}

@AnyLogicInternalCodegenAPI

public void _assign_RequiredCapEx_Formula_xjal() {

RequiredCapEx =

0.05 * main.pipe.CG100() * UnitPriceCapEx_$_Mile

;

}

@AnyLogicInternalCodegenAPI

public void _assign_AvailableCapEx_Formula_xjal() {

AvailableCapEx =

Revenue - OpEx()

;

}

@AnyLogicInternalCodegenAPI

public void _assign_AgencyWaterSupply_Formula_xjal() {

AgencyWaterSupply =

AnnualDemand - main.pipe.AnnualTotalWaterLoss * 1000000

;

}

@AnyLogicInternalCodegenAPI

public void _assign_AgencyWaterDemand_Formula_xjal() {

AgencyWaterDemand =

AnnualDemand

;

}

@AnyLogicInternalCodegenAPI

public void formulasExecute_xjal() {

super.formulasExecute_xjal();

_assign_AnnualDemand_Formula_xjal();

_assign_Revenue_Formula_xjal();

_assign_AverageCondition_Formula_xjal();

_assign_RequiredCapEx_Formula_xjal();

_assign_AvailableCapEx_Formula_xjal();

_assign_AgencyWaterSupply_Formula_xjal();

_assign_AgencyWaterDemand_Formula_xjal();

}

@AnyLogicInternalCodegenAPI

protected SDIntegrationManager integrationManager_xjal = null;

@AnyLogicInternalCodegenAPI

public SDIntegrationManager getIntegrationManager_xjal() {

if (integrationManager_xjal == null) {

integrationManager_xjal = new SDIntegrationManager( getDifferentialFlatEquationsCount_xjal(), getRuntimeAlgebraicFlatEquationsCount_xjal(), getRuntimeFormulaFlatEquationsCount_xjal() );

}

return integrationManager_xjal;

}

@Override

@AnyLogicInternalCodegenAPI

public int getRuntimeFormulaFlatEquationsCount_xjal() {

return super.getRuntimeFormulaFlatEquationsCount_xjal() + 7;

}

@Override

@AnyLogicInternalCodegenAPI

public int getInitialFormulaFlatEquationsCount_xjal() {

return super.getInitialFormulaFlatEquationsCount_xjal() + 7;

}

@AnyLogicInternalCodegenAPI

private static Map<String, IElementDescriptor> elementDesciptors_xjal = null;

@AnyLogicInternalCodegenAPI

@Override

public Map<String, IElementDescriptor> getElementDesciptors() {

if (elementDesciptors_xjal == null) {

elementDesciptors_xjal = createElementDescriptors(super.getElementDesciptors(), Agency.class);

}

return elementDesciptors_xjal;

}

@AnyLogicCustomProposalPriority(type = AnyLogicCustomProposalPriority.Type.STATIC_ELEMENT)

public static final Scale scale = new Scale( 10.0 );

@Override

public Scale getScale() {

return scale;

}

// Events

public EventTimeout YearlyEvent = new EventTimeout(this);

@AnyLogicInternalCodegenAPI

public EventTimeout _autoCreatedDS_xjal = new EventTimeout(this);

@Override

@AnyLogicInternalCodegenAPI

public String getNameOf( EventTimeout _e ) {

if( _e == YearlyEvent ) return "YearlyEvent";

if( _e == _autoCreatedDS_xjal ) return "Auto-created DataSets auto update event";

return super.getNameOf( _e );

}

@Override

@AnyLogicInternalCodegenAPI

public EventTimeout.Mode getModeOf( EventTimeout _e ) {

if ( _e == YearlyEvent ) return EVENT_TIMEOUT_MODE_CYCLIC;

if ( _e == _autoCreatedDS_xjal ) return EVENT_TIMEOUT_MODE_CYCLIC;

return super.getModeOf( _e );

}

@Override

@AnyLogicInternalCodegenAPI

public double getFirstOccurrenceTime( EventTimeout _e ) {

double _t;

if ( _e == YearlyEvent ) {

_t =

0

;

_t = toModelTime( _t, YEAR );

return _t;

}

if ( _e == _autoCreatedDS_xjal ) {

_t =

0

;

_t = toModelTime( _t, YEAR );

return _t;

}

return super.getFirstOccurrenceTime( _e );

}

@Override

@AnyLogicInternalCodegenAPI

public double evaluateTimeoutOf( EventTimeout _e ) {

double _t;

if( _e == YearlyEvent) {

_t =

1

;

_t = toModelTime( _t, YEAR );

return _t;

}

if( _e == _autoCreatedDS_xjal) {

_t =

1

;

_t = toModelTime( _t, YEAR );

return _t;

}

return super.evaluateTimeoutOf( _e );

}

@Override

@AnyLogicInternalCodegenAPI

public void executeActionOf( EventTimeout _e ) {

if ( _e == YearlyEvent ) {

return;

}

if ( _e == _autoCreatedDS_xjal ) {

_ds_AverageCondition.update();

_ds_RequiredCapEx.update();

_ds_Revenue.update();

_ds_AvailableCapEx.update();

_ds_AnnualDemand.update();

_ds_AgencyWaterSupply.update();

_ds_AgencyWaterDemand.update();

return;

}

super.executeActionOf( _e );

}

// Functions

double

ConditionMultiplierOpEx( double AverageCondition ) {

return 1.4877*exp(0.0449*AverageCondition);

}

double

OpEx( ) {

return UnitPriceOpEx_$_Mile_Year*TotalPipeLength*(1+ConditionMultiplierOpEx(AverageCondition)/100);

}

HashMap<Integer, Double>

renewalRS( ) {

double renewal_length = 0;

HashMap<Integer, Double> hmap = new HashMap<Integer, Double>();

if(AvailableCapEx >= RequiredCapEx){

renewal_length = 0.05*main.pipe.CG100(); //renewal is only 5% of CG100

UserFeeDecline = (AvailableCapEx - RequiredCapEx)/AnnualDemand;

UserFees = UserFees - UserFeeDecline;

//System.out.println("Decline - "+UserFees);

}

else if(AvailableCapEx < RequiredCapEx){

if(AvailableCapEx > 0){

renewal_length = AvailableCapEx/UnitPriceCapEx_$_Mile;

}

UserFeeHike = (RequiredCapEx - AvailableCapEx)/AnnualDemand;

UserFees = UserFees + UserFeeHike;

//System.out.println("Hike - "+UserFees);

}

else{

UserFeeDecline=0;

UserFeeHike=0;

//user fee stays the same

}

if(UserFees < 0){

UserFees = 0;

}

hmap.put(80, 0.0);

hmap.put(100, renewal_length);

return hmap;

}

HashMap<Integer, Double>

renewalRA( ) {

//double renewal_length_80 = 0;

double average_breakage = main.pipe.AverageFiveYearBreakage();

//HashMap<Integer, Double> hmap = new HashMap<Integer, Double>();

HashMap<Integer, Double> hmap = renewalRS();

if(average_breakage > main.BreakToleranceChoice){

//double p_cg80 = main.pipe.CalculateL80PercentageRA(renewalRS().get(100), (YearBudget)/UnitPriceCapEx_$_Mile);

hmap = main.pipe.CalculateL80PercentageRA(hmap, (YearBudget)/UnitPriceCapEx_$_Mile);

//renewal_length_80 = main.pipe.CG80() - main.pipe.CG80()*p_cg80;

}

//hmap.put(100, renewalRS().get(100));

//hmap.put(80, renewal_length_80);

return hmap;

}

HashMap<Integer, Double>

renewal( ) {

HashMap<Integer, Double> hmap = new HashMap<Integer, Double>();

switch(main.RiskAttitudeChoice){

case 0: //RA

hmap = renewalRA();

break;

case 1: //RN

hmap = renewalRN();

break;

case 2: //RS

hmap = renewalRS();

break;

}

return hmap;

}

HashMap<Integer, Double>

renewalRN( ) {

double average_breakage = main.pipe.AverageFiveYearBreakage();

//HashMap<Integer, Double> hmap = new HashMap<Integer, Double>();

HashMap<Integer, Double> hmap = renewalRS();

if(AverageCondition > main.DesiredAverageConditionChoice){

//double p_cg80 = main.pipe.CalculateL80PercentageRA(renewalRS().get(100), (YearBudget)/UnitPriceCapEx_$_Mile);

hmap = main.pipe.CalculateL80PercentageRN(hmap, (YearBudget)/UnitPriceCapEx_$_Mile);

//renewal_length_80 = main.pipe.CG80() - main.pipe.CG80()*p_cg80;

}

//hmap.put(100, renewalRS().get(100));

//hmap.put(80, renewal_length_80);

return hmap;

}

/**

* Auto-created data set(s) for AverageCondition

*/

@AnyLogicInternalCodegenAPI

public DataSet _ds_AverageCondition = new DataSet( 100, new DataUpdater_xjal() {

double _lastUpdateTime = Double.NaN;

@Override

public void update( DataSet _d ) {

if ( time() == _lastUpdateTime ) { return; }

_d.add( time(), Agency.this.AverageCondition );

_lastUpdateTime = time();

}

} );

/**

* Auto-created data set(s) for RequiredCapEx

*/

@AnyLogicInternalCodegenAPI

public DataSet _ds_RequiredCapEx = new DataSet( 100, new DataUpdater_xjal() {

double _lastUpdateTime = Double.NaN;

@Override

public void update( DataSet _d ) {

if ( time() == _lastUpdateTime ) { return; }

_d.add( time(), Agency.this.RequiredCapEx );

_lastUpdateTime = time();

}

} );

/**

* Auto-created data set(s) for Revenue

*/

@AnyLogicInternalCodegenAPI

public DataSet _ds_Revenue = new DataSet( 100, new DataUpdater_xjal() {

double _lastUpdateTime = Double.NaN;

@Override

public void update( DataSet _d ) {

if ( time() == _lastUpdateTime ) { return; }

_d.add( time(), Agency.this.Revenue );

_lastUpdateTime = time();

}

} );

/**

* Auto-created data set(s) for AvailableCapEx

*/

@AnyLogicInternalCodegenAPI

public DataSet _ds_AvailableCapEx = new DataSet( 100, new DataUpdater_xjal() {

double _lastUpdateTime = Double.NaN;

@Override

public void update( DataSet _d ) {

if ( time() == _lastUpdateTime ) { return; }

_d.add( time(), Agency.this.AvailableCapEx );

_lastUpdateTime = time();

}

} );

/**

* Auto-created data set(s) for AnnualDemand

*/

@AnyLogicInternalCodegenAPI

public DataSet _ds_AnnualDemand = new DataSet( 100, new DataUpdater_xjal() {

double _lastUpdateTime = Double.NaN;

@Override

public void update( DataSet _d ) {

if ( time() == _lastUpdateTime ) { return; }

_d.add( time(), Agency.this.AnnualDemand );

_lastUpdateTime = time();

}

} );

/**

* Auto-created data set(s) for AgencyWaterSupply

*/

@AnyLogicInternalCodegenAPI

public DataSet _ds_AgencyWaterSupply = new DataSet( 100, new DataUpdater_xjal() {

double _lastUpdateTime = Double.NaN;

@Override

public void update( DataSet _d ) {

if ( time() == _lastUpdateTime ) { return; }

_d.add( time(), Agency.this.AgencyWaterSupply );

_lastUpdateTime = time();

}

} );

/**

* Auto-created data set(s) for AgencyWaterDemand

*/

@AnyLogicInternalCodegenAPI

public DataSet _ds_AgencyWaterDemand = new DataSet( 100, new DataUpdater_xjal() {

double _lastUpdateTime = Double.NaN;

@Override

public void update( DataSet _d ) {

if ( time() == _lastUpdateTime ) { return; }

_d.add( time(), Agency.this.AgencyWaterDemand );

_lastUpdateTime = time();

}

} );

// View areas

public ViewArea _origin_VA = new ViewArea( this, "[Origin]", 0, 0, 1000.0, 590.0 );

@Override

@AnyLogicInternalCodegenAPI

public int getViewAreas(Map<String, ViewArea> _output) {

if ( _output != null ) {

_output.put( "_origin_VA", this._origin_VA );

}

return 1 + super.getViewAreas( _output );

}

@AnyLogicInternalCodegenAPI

protected static final Font _text2_Font = new Font("SansSerif", 0, 10 );

@AnyLogicInternalCodegenAPI

protected static final int _image = 1;

@AnyLogicInternalCodegenAPI

protected static final int _text2 = 2;

/** Internal constant, shouldn't be accessed by user */

@AnyLogicInternalCodegenAPI

protected static final int _SHAPE_NEXT_ID_xjal = 3;

/**

* Top-level presentation group id

*/

@AnyLogicInternalCodegenAPI

protected static final int _presentation = 0;

@AnyLogicInternalCodegenAPI

public boolean isPublicPresentationDefined() {

return true;

}

@AnyLogicInternalCodegenAPI

public boolean isEmbeddedAgentPresentationVisible( Agent _a ) {

return super.isEmbeddedAgentPresentationVisible( _a );

}

/**

* Top-level icon group id

*/

@AnyLogicInternalCodegenAPI

protected static final int _icon = -1;

protected ShapeImage image;

/**

* <i>This method should not be called by user</i>

*/

@AnyLogicInternalCodegenAPI

private void _text2_SetDynamicParams_xjal( ShapeText shape ) {

shape.setText(

OpEx()

);

}

protected ShapeText text2;

@AnyLogicInternalCodegenAPI

private void _createPersistentElementsBP0_xjal() {

image = new ShapeImage(

Agency.this, SHAPE_DRAW_2D3D, false, 160.0, -260.0, 0.0, 0.0,

390.0, 240.0, "/waterinfrastructure/",

new String[]{"pic.png",} );

text2 = new ShapeText(

SHAPE_DRAW_2D, true,50.0, 260.0, 0.0, 0.0,

dodgerBlue,"text",

_text2_Font, ALIGNMENT_LEFT ) {

@Override

public void updateDynamicProperties(boolean publicOnly) {

_text2_SetDynamicParams_xjal( this );

super.updateDynamicProperties(publicOnly);

}

};

}

@AnyLogicInternalCodegenAPI

private void _createPersistentElementsAP0_xjal() {

}

// Static initialization of persistent elements

{

_createPersistentElementsBP0_xjal();

}

protected ShapeTopLevelPresentationGroup presentation;

protected ShapeGroup icon;

@Override

@AnyLogicInternalCodegenAPI

public Object getPersistentShape( int _shape ) {

switch (_shape) {

case _presentation: return presentation;

case _icon: return icon;

case _image: return image;

case _text2: return text2;

default: return super.getPersistentShape( _shape );

}

}

@Override

@AnyLogicInternalCodegenAPI

public String getNameOfShape_xjal( Object _shape ) {

try {

if ( _shape == null ) return null;

String _name_xjal;

_name_xjal = checkNameOfShape_xjal( _shape, presentation, "presentation" ); if (_name_xjal != null) return _name_xjal;

_name_xjal = checkNameOfShape_xjal( _shape, icon, "icon" ); if (_name_xjal != null) return _name_xjal;

_name_xjal = checkNameOfShape_xjal( _shape, image, "image" ); if (_name_xjal != null) return _name_xjal;

_name_xjal = checkNameOfShape_xjal( _shape, text2, "text2" ); if (_name_xjal != null) return _name_xjal;

} catch (Exception e) {

return null;

}

return super.getNameOfShape_xjal( _shape );

}

@AnyLogicInternalCodegenAPI

private void drawModelElements_Events_xjal(Panel _panel, Graphics2D _g, boolean _publicOnly, boolean _isSuperClass ) {

if (!_publicOnly) {

drawEvent( _panel, _g, -90, 160, 10, 0, "YearlyEvent", YearlyEvent );

}

}

@AnyLogicInternalCodegenAPI

private void drawModelElements_Parameters_xjal(Panel _panel, Graphics2D _g, boolean _publicOnly, boolean _isSuperClass ) {

if (!_publicOnly) {

drawParameter( _panel, _g, 160, 20, 10, 0, "TotalPipeLength", TotalPipeLength, 0 );

}

if (!_publicOnly) {

drawParameter( _panel, _g, 460, 120, 10, 0, "UnitPriceCapEx_$_Mile", UnitPriceCapEx_$_Mile, 0 );

}

if (!_publicOnly) {

drawParameter( _panel, _g, 50, 310, 10, 0, "InputUserFees", InputUserFees, 0 );

}

if (!_publicOnly) {

drawParameter( _panel, _g, -130, -20, 10, 0, "CapitalImprovement", CapitalImprovement, 0 );

}

}

@AnyLogicInternalCodegenAPI

private void drawModelElements_PlainVariables_xjal(Panel _panel, Graphics2D _g, boolean _publicOnly, boolean _isSuperClass ) {

if (!_publicOnly) {

drawPlainVariable( _panel, _g, 220, 230, 10, 0, "UserFees", UserFees, false );

}

if (!_publicOnly) {

drawPlainVariable( _panel, _g, 570, 250, 10, 0, "TotalExToDate", TotalExToDate, false );

}

if (!_publicOnly) {

drawPlainVariable( _panel, _g, 420, 300, 10, 0, "UserFeeHike", UserFeeHike, false );

}

if (!_publicOnly) {

drawPlainVariable( _panel, _g, 420, 340, 10, 0, "UserFeeDecline", UserFeeDecline, false );

}

if (!_publicOnly) {

drawPlainVariable( _panel, _g, 160, 60, 10, 0, "UnitPriceOpEx_$_Mile_Year", UnitPriceOpEx_$_Mile_Year, false );

}

if (!_publicOnly) {

drawPlainVariable( _panel, _g, -90, 120, 10, 0, "YearBudget", YearBudget, false );

}

}

@AnyLogicInternalCodegenAPI

private void drawModelElements_AuxVariables_xjal(Panel _panel, Graphics2D _g, boolean _publicOnly, boolean _isSuperClass ) {

if (!_publicOnly) {

drawAuxiliaryVariable( _panel, _g, 460, 60, 20, 0, "AverageCondition", AverageCondition, null, 0 );

}

if (!_publicOnly) {

drawAuxiliaryVariable( _panel, _g, 460, 30, 10, 0, "RequiredCapEx", RequiredCapEx, null, 0 );

}

if (!_publicOnly) {

drawAuxiliaryVariable( _panel, _g, 310, 220, 20, 0, "Revenue", Revenue, null, 0 );

}

if (!_publicOnly) {

drawAuxiliaryVariable( _panel, _g, 460, 190, 0, -20, "AvailableCapEx", AvailableCapEx, null, 0 );

}

if (!_publicOnly) {

drawAuxiliaryVariable( _panel, _g, 200, 140, 15, 0, "AnnualDemand", AnnualDemand, null, 0 );

}

if (!_publicOnly) {

drawAuxiliaryVariable( _panel, _g, 180, 310, 15, 0, "AgencyWaterSupply", AgencyWaterSupply, null, 0 );

}

if (!_publicOnly) {

drawAuxiliaryVariable( _panel, _g, 180, 350, 15, 0, "AgencyWaterDemand", AgencyWaterDemand, null, 0 );

}

}

@AnyLogicInternalCodegenAPI

private static void createLinkArcs_xjal() {

_arc_PD_1467923390605_xjal = new Arc2D.Double(255.47082389845556, 182.35411664617462, 312.50000000000017, 312.50000000000017, 127.31735229492188, -49.81413269042969, Arc2D.OPEN);

_arc_PD_1467925297405_xjal = new Arc2D.Double(439.99999999999994, 14.375000000000078, 121.25000000000001, 121.25000000000001, 219.41138458251953, -73.14295196533203, Arc2D.OPEN);

_arc_PD_1467995769472_xjal = new Arc2D.Double(67.24936250130138, 139.79774879477463, 251.24999999999991, 251.24999999999991, 82.6427001953125, -54.60259246826172, Arc2D.OPEN);

_arc_PD_1469198599678_xjal = new Arc2D.Double(-175.06420070154684, 11.646852475300335, 386.24999999999943, 386.24999999999943, 16.92580795288086, -45.49094772338867, Arc2D.OPEN);

_arc_PD_1469198622332_xjal = new Arc2D.Double(-365.0422214702673, -68.54568058731104, 576.2499999999997, 576.2499999999997, 14.243587493896484, -38.17460250854492, Arc2D.OPEN);

}

@AnyLogicInternalCodegenAPI

protected static Arc2D.Double _arc_PD_1467923390605_xjal;

@AnyLogicInternalCodegenAPI

protected static Arc2D.Double _arc_PD_1467925297405_xjal;

@AnyLogicInternalCodegenAPI

protected static Arc2D.Double _arc_PD_1467995769472_xjal;

@AnyLogicInternalCodegenAPI

protected static Arc2D.Double _arc_PD_1469198599678_xjal;

@AnyLogicInternalCodegenAPI

protected static Arc2D.Double _arc_PD_1469198622332_xjal;

static {

createLinkArcs_xjal();

}

@AnyLogicInternalCodegenAPI

private void drawModelElements_Links_xjal(Panel _panel, Graphics2D _g, boolean _publicOnly, boolean _isSuperClass ) {

if (!_publicOnly) {

drawLink( _panel, _g, _arc_PD_1467923390605_xjal, null, 1, null, 0.95f, 0 );

}

if (!_publicOnly) {

drawLink( _panel, _g, _arc_PD_1467925297405_xjal, null, 1, null, 0.95f, 0 );

}

if (!_publicOnly) {

drawLink( _panel, _g, _arc_PD_1467995769472_xjal, null, 1, null, 0.95f, 0 );

}

if (!_publicOnly) {

drawLink( _panel, _g, _arc_PD_1469198599678_xjal, null, 1, null, 0.95f, 0 );

}

if (!_publicOnly) {

drawLink( _panel, _g, _arc_PD_1469198622332_xjal, null, 1, null, 0.95f, 0 );

}

}

@AnyLogicInternalCodegenAPI

private void drawModelElements_Functions_xjal(Panel _panel, Graphics2D _g, boolean _publicOnly, boolean _isSuperClass ) {

if (!_publicOnly) {

drawFunction( _panel, _g, 50, 210, 10, 0, "ConditionMultiplierOpEx");

}

if (!_publicOnly) {

drawFunction( _panel, _g, 50, 250, 10, 0, "OpEx");

}

if (!_publicOnly) {

drawFunction( _panel, _g, -90, 280, 10, 0, "renewalRS");

}

if (!_publicOnly) {

drawFunction( _panel, _g, -90, 200, 10, 0, "renewalRA");

}

if (!_publicOnly) {

drawFunction( _panel, _g, -90, 320, 10, 0, "renewal");

}

if (!_publicOnly) {

drawFunction( _panel, _g, -90, 240, 10, 0, "renewalRN");

}

}

@AnyLogicInternalCodegenAPI

private void drawModelElements_AgentLinks_xjal(Panel _panel, Graphics2D _g, boolean _publicOnly, boolean _isSuperClass ) {

if (_publicOnly) { return; }

drawLinkToContainer( _panel, _g, 50, -100, 10, 0, "main", main );

drawLinkToAgent( _panel, _g, 50, -50, 15, 0, "connections", true, connections );

}

@Override

@AnyLogicInternalCodegenAPI

public void drawModelElements( Panel _panel, Graphics2D _g, boolean _publicOnly, boolean _isSuperClass ) {

super.drawModelElements( _panel, _g, _publicOnly, true );

drawModelElements_Events_xjal( _panel, _g, _publicOnly, _isSuperClass );

drawModelElements_Parameters_xjal( _panel, _g, _publicOnly, _isSuperClass );

drawModelElements_PlainVariables_xjal( _panel, _g, _publicOnly, _isSuperClass );

drawModelElements_AuxVariables_xjal( _panel, _g, _publicOnly, _isSuperClass );

drawModelElements_Links_xjal( _panel, _g, _publicOnly, _isSuperClass );

drawModelElements_Functions_xjal( _panel, _g, _publicOnly, _isSuperClass );

drawModelElements_AgentLinks_xjal( _panel, _g, _publicOnly, _isSuperClass );

}

@AnyLogicInternalCodegenAPI

private boolean onClickModelAt_AgentLinks_xjal( Panel _panel, double _x, double _y, int _clickCount, boolean _publicOnly, boolean _isSuperClass ) {

if ( modelElementContains(_x, _y, 50, -100) ) {

if ( _clickCount == 2 ) {

_panel.browseAgent_xjal( 50, -100, this, "main" );

} else {

_panel.addInspect( 50, -100, this, "main" );

}

return true;

}

if ( modelElementContains(_x, _y, 50, -50) ) {

_panel.addInspect_xjal( 50, -50, this, "connections", Panel.INSPECT_CONNECTIONS_xjal );

return true;

}

return false;

}

@AnyLogicInternalCodegenAPI

private boolean onClickModelAt_Parameters_xjal( Panel _panel, double _x, double _y, int _clickCount, boolean _publicOnly, boolean _isSuperClass ) {

if( !_publicOnly && modelElementContains(_x, _y, 160, 20) ) {

_panel.addInspect( 160, 20, this, "TotalPipeLength" );

return true;

}

if( !_publicOnly && modelElementContains(_x, _y, 460, 120) ) {

_panel.addInspect( 460, 120, this, "UnitPriceCapEx_$_Mile" );

return true;

}

if( !_publicOnly && modelElementContains(_x, _y, 50, 310) ) {

_panel.addInspect( 50, 310, this, "InputUserFees" );

return true;

}

if( !_publicOnly && modelElementContains(_x, _y, -130, -20) ) {

_panel.addInspect( -130, -20, this, "CapitalImprovement" );

return true;

}

return false;

}

@AnyLogicInternalCodegenAPI

private boolean onClickModelAt_PlainVariables_xjal( Panel _panel, double _x, double _y, int _clickCount, boolean _publicOnly, boolean _isSuperClass ) {

if( !_publicOnly && modelElementContains(_x, _y, 220, 230) ) {

_panel.addInspect( 220, 230, this, "UserFees" );

return true;

}

if( !_publicOnly && modelElementContains(_x, _y, 570, 250) ) {

_panel.addInspect( 570, 250, this, "TotalExToDate" );

return true;

}

if( !_publicOnly && modelElementContains(_x, _y, 420, 300) ) {

_panel.addInspect( 420, 300, this, "UserFeeHike" );

return true;

}

if( !_publicOnly && modelElementContains(_x, _y, 420, 340) ) {

_panel.addInspect( 420, 340, this, "UserFeeDecline" );

return true;

}

if( !_publicOnly && modelElementContains(_x, _y, 160, 60) ) {

_panel.addInspect( 160, 60, this, "UnitPriceOpEx_$_Mile_Year" );

return true;

}

if( !_publicOnly && modelElementContains(_x, _y, -90, 120) ) {

_panel.addInspect( -90, 120, this, "YearBudget" );

return true;

}

return false;

}

@AnyLogicInternalCodegenAPI

private boolean onClickModelAt_AuxVariables_xjal( Panel _panel, double _x, double _y, int _clickCount, boolean _publicOnly, boolean _isSuperClass ) {

if( !_publicOnly && modelElementContains(_x, _y, 460, 60) ) {

_panel.addInspect_xjal( 460, 60, this, "AverageCondition", Panel.INSPECT_READ_ONLY_xjal );

return true;

}

if( !_publicOnly && modelElementContains(_x, _y, 460, 30) ) {

_panel.addInspect_xjal( 460, 30, this, "RequiredCapEx", Panel.INSPECT_READ_ONLY_xjal );

return true;

}

if( !_publicOnly && modelElementContains(_x, _y, 310, 220) ) {

_panel.addInspect_xjal( 310, 220, this, "Revenue", Panel.INSPECT_READ_ONLY_xjal );

return true;

}

if( !_publicOnly && modelElementContains(_x, _y, 460, 190) ) {

_panel.addInspect_xjal( 460, 190, this, "AvailableCapEx", Panel.INSPECT_READ_ONLY_xjal );

return true;

}

if( !_publicOnly && modelElementContains(_x, _y, 200, 140) ) {

_panel.addInspect_xjal( 200, 140, this, "AnnualDemand", Panel.INSPECT_READ_ONLY_xjal );

return true;

}

if( !_publicOnly && modelElementContains(_x, _y, 180, 310) ) {

_panel.addInspect_xjal( 180, 310, this, "AgencyWaterSupply", Panel.INSPECT_READ_ONLY_xjal );

return true;

}

if( !_publicOnly && modelElementContains(_x, _y, 180, 350) ) {

_panel.addInspect_xjal( 180, 350, this, "AgencyWaterDemand", Panel.INSPECT_READ_ONLY_xjal );

return true;

}

return false;

}

@AnyLogicInternalCodegenAPI

private boolean onClickModelAt_Events_xjal( Panel _panel, double _x, double _y, int _clickCount, boolean _publicOnly, boolean _isSuperClass ) {

if( !_publicOnly && modelElementContains(_x, _y, -90, 160) ) {

_panel.addInspect( -90, 160, this, "YearlyEvent" );

return true;

}

return false;

}

@Override

@AnyLogicInternalCodegenAPI

public boolean onClickModelAt( Panel _panel, double _x, double _y, int _clickCount, boolean _publicOnly, boolean _isSuperClass ) {

if ( onClickModelAt_AgentLinks_xjal( _panel, _x, _y, _clickCount, _publicOnly, _isSuperClass ) ) { return true; }

if ( onClickModelAt_Parameters_xjal( _panel, _x, _y, _clickCount, _publicOnly, _isSuperClass ) ) { return true; }

if ( onClickModelAt_PlainVariables_xjal( _panel, _x, _y, _clickCount, _publicOnly, _isSuperClass ) ) { return true; }

if ( onClickModelAt_AuxVariables_xjal( _panel, _x, _y, _clickCount, _publicOnly, _isSuperClass ) ) { return true; }

if ( onClickModelAt_Events_xjal( _panel, _x, _y, _clickCount, _publicOnly, _isSuperClass ) ) { return true; }

return super.onClickModelAt( _panel, _x, _y, _clickCount, _publicOnly, true );

}

/**

* Constructor

*/

public Agency( Engine engine, Agent owner, AgentList<? extends Agency> ownerPopulation ) {

super( engine, owner, ownerPopulation );

instantiateBaseStructureThis_xjal();

}

@AnyLogicInternalCodegenAPI

public void onOwnerChanged_xjal() {

super.onOwnerChanged_xjal();

setupReferences_xjal();

}

@AnyLogicInternalCodegenAPI

public void instantiateBaseStructure_xjal() {

super.instantiateBaseStructure_xjal();

instantiateBaseStructureThis_xjal();

}

@AnyLogicInternalCodegenAPI

private void instantiateBaseStructureThis_xjal() {

setupReferences_xjal();

// Registering in Engine continuous part

getEngine().registerAgentWithEquations( this );

}

@AnyLogicInternalCodegenAPI

private void setupReferences_xjal() {

main = get_Main();

}

/**

* Simple constructor. Please add created agent to some population by calling goToPopulation() function

*/

public Agency() {

}

/**

* Simple constructor. Please add created agent to some population by calling goToPopulation() function

*/

public Agency( double TotalPipeLength, double UnitPriceCapEx_$_Mile, double InputUserFees, double CapitalImprovement ) {

markParametersAreSet();

this.TotalPipeLength = TotalPipeLength;

this.UnitPriceCapEx_$_Mile = UnitPriceCapEx_$_Mile;

this.InputUserFees = InputUserFees;

this.CapitalImprovement = CapitalImprovement;

}

@Override

@AnyLogicInternalCodegenAPI

public void doCreate() {

super.doCreate();

// Assigning initial values for plain variables

setupPlainVariables_Agency_xjal();

// Dynamic initialization of persistent elements

_createPersistentElementsAP0_xjal();

presentation = new ShapeTopLevelPresentationGroup( Agency.this, true, 0, 0, 0, 0 , image, text2 );

icon = new ShapeGroup( Agency.this, true, 0, 0, 0 );

// Port connectors with non-replicated objects

// Creating replicated embedded objects

setupInitialConditions_xjal( Agency.class );

}

@AnyLogicInternalCodegenAPI

public void setupExt_xjal(AgentExtension _ext) {

// Agent properties setup

if ( _ext instanceof ExtAgentWithSpatialMetrics && _ext instanceof ExtWithSpaceType ) {

double _value;

_value =

10

;

((ExtAgentWithSpatialMetrics) _ext).setSpeed( _value, MPS );

}

}

@Override

@AnyLogicInternalCodegenAPI

public void doStart() {

super.doStart();

YearlyEvent.start();

_autoCreatedDS_xjal.start();

}

/**

* Assigning initial values for plain variables<br>

* <em>This method isn't designed to be called by user and may be removed in future releases.</em>

*/

@AnyLogicInternalCodegenAPI

public void setupPlainVariables_xjal() {

setupPlainVariables_Agency_xjal();

}

/**

* Assigning initial values for plain variables<br>

* <em>This method isn't designed to be called by user and may be removed in future releases.</em>

*/

@AnyLogicInternalCodegenAPI

private void setupPlainVariables_Agency_xjal() {

UserFees =

InputUserFees

;

TotalExToDate =

0

;

UserFeeHike =

0

;

UserFeeDecline =

0

;

UnitPriceOpEx_$_Mile_Year =

60000

;

YearBudget =

CapitalImprovement/5

;

}

// User API -----------------------------------------------------

public Main get_Main() {

{

Agent owner = getOwner();

if ( owner instanceof Main ) return (Main) owner;

}

return null;

}

/**

* Read-only variable. <em>Shouldn't be modified by user.</em>

*/

@AnyLogicCustomSerialization(AnyLogicCustomSerializationMode.REFERENCE)

public transient waterinfrastructure.Main main;

@AnyLogicInternalCodegenAPI

static LinkToAgentAnimationSettings _connections_commonAnimationSettings_xjal = new LinkToAgentAnimationSettingsImpl( false, black, 1.0, LINE_STYLE_SOLID, ARROW_NONE, 0.0 );

public LinkToAgentCollection<Agent, Agent> connections = new LinkToAgentStandardImpl<Agent, Agent>(this, _connections_commonAnimationSettings_xjal);

@Override

public LinkToAgentCollection<? extends Agent, ? extends Agent> getLinkToAgentStandard_xjal() {

return connections;

}

@AnyLogicInternalCodegenAPI

public void drawLinksToAgents(boolean _underAgents_xjal, LinkToAgentAnimator _animator_xjal) {

super.drawLinksToAgents(_underAgents_xjal, _animator_xjal);

if ( _underAgents_xjal ) {

_animator_xjal.drawLink( this, connections, true, true );

}

}

public AgentList<? extends Agency> getPopulation() {

return (AgentList<? extends Agency>) super.getPopulation();

}

public List<? extends Agency> agentsInRange( double distance ) {

return (List<? extends Agency>) super.agentsInRange( distance );

}

@Override

@AnyLogicInternalCodegenAPI

public boolean isLoggingToDB(EventOriginator _e) {

if ( _e == _autoCreatedDS_xjal ) return false;

return super.isLoggingToDB( _e );

}

@AnyLogicInternalCodegenAPI

public void onDestroy() {

YearlyEvent.onDestroy();

_autoCreatedDS_xjal.onDestroy();

// Unregistering in Engine continuous part

getEngine().unregisterAgentWithEquations( this );

_ds_AverageCondition.destroyUpdater_xjal();

_ds_RequiredCapEx.destroyUpdater_xjal();

_ds_Revenue.destroyUpdater_xjal();

_ds_AvailableCapEx.destroyUpdater_xjal();

_ds_AnnualDemand.destroyUpdater_xjal();

_ds_AgencyWaterSupply.destroyUpdater_xjal();

_ds_AgencyWaterDemand.destroyUpdater_xjal();

super.onDestroy();

}

}

public class Pipe extends Agent

{

// Parameters

public

double RehabFraction;

/**

* Returns default value for parameter <code>RehabFraction</code>.

* <i>This method should not be called by user</i>

*/

@AnyLogicInternalCodegenAPI

public double _RehabFraction_DefaultValue_xjal() {

final Pipe self = this;

return 0.0;

}

public void set_RehabFraction( double RehabFraction ) {

if (RehabFraction == this.RehabFraction) {

return;

}

double _oldValue_xjal = this.RehabFraction;

this.RehabFraction = RehabFraction;

onChange_RehabFraction_xjal( _oldValue_xjal );

onChange();

}

/**

* Calls "On change" action for parameter RehabFraction.<br>

* Note that 'oldValue' in that action will be unavailable if this method is called by user

* (current parameter value will be passed as 'oldValue').<br>

* Please call <code>set_RehabFraction()</code> method instead.

*/

protected void onChange_RehabFraction() {

onChange_RehabFraction_xjal( RehabFraction );

}

@AnyLogicInternalCodegenAPI

protected void onChange_RehabFraction_xjal( double oldValue ) {

}

public

double TotalLengthPipeMile;

/**

* Returns default value for parameter <code>TotalLengthPipeMile</code>.

* <i>This method should not be called by user</i>

*/

@AnyLogicInternalCodegenAPI

public double _TotalLengthPipeMile_DefaultValue_xjal() {

final Pipe self = this;

return 0.0;

}

public void set_TotalLengthPipeMile( double TotalLengthPipeMile ) {

if (TotalLengthPipeMile == this.TotalLengthPipeMile) {

return;

}

double _oldValue_xjal = this.TotalLengthPipeMile;

this.TotalLengthPipeMile = TotalLengthPipeMile;

onChange_TotalLengthPipeMile_xjal( _oldValue_xjal );

onChange();

}

/**

* Calls "On change" action for parameter TotalLengthPipeMile.<br>

* Note that 'oldValue' in that action will be unavailable if this method is called by user

* (current parameter value will be passed as 'oldValue').<br>

* Please call <code>set_TotalLengthPipeMile()</code> method instead.

*/

protected void onChange_TotalLengthPipeMile() {

onChange_TotalLengthPipeMile_xjal( TotalLengthPipeMile );

}

@AnyLogicInternalCodegenAPI

protected void onChange_TotalLengthPipeMile_xjal( double oldValue ) {

}

@Override

public void setParametersToDefaultValues() {

super.setParametersToDefaultValues();

RehabFraction = _RehabFraction_DefaultValue_xjal();

TotalLengthPipeMile = _TotalLengthPipeMile_DefaultValue_xjal();

}

@Override

public boolean setParameter(String _name_xjal, Object _value_xjal, boolean _callOnChange_xjal) {

switch ( _name_xjal ) {

case "RehabFraction":

if ( _callOnChange_xjal ) {

set_RehabFraction( ((Number) _value_xjal).doubleValue() );

} else {

RehabFraction = ((Number) _value_xjal).doubleValue();

}

return true;

case "TotalLengthPipeMile":

if ( _callOnChange_xjal ) {

set_TotalLengthPipeMile( ((Number) _value_xjal).doubleValue() );

} else {

TotalLengthPipeMile = ((Number) _value_xjal).doubleValue();

}

return true;

default:

return super.setParameter( _name_xjal, _value_xjal, _callOnChange_xjal );

}

}

@Override

public <T> T getParameter(String _name_xjal) {

Object _result_xjal;

switch ( _name_xjal ) {

case "RehabFraction": _result_xjal = RehabFraction; break;

case "TotalLengthPipeMile": _result_xjal = TotalLengthPipeMile; break;

default: _result_xjal = super.getParameter( _name_xjal ); break;

}

return (T) _result_xjal;

}

@AnyLogicInternalCodegenAPI

private static String[] _parameterNames_xjal;

@Override

public String[] getParameterNames() {

String[] result = _parameterNames_xjal;

if (result == null) {

List<String> list = new ArrayList<>( Arrays.asList( super.getParameterNames() ) );

list.add( "RehabFraction" );

list.add( "TotalLengthPipeMile" );

result = list.toArray( new String[ list.size() ] );

_parameterNames_xjal = result;

}

return result;

}

// Plain Variables

public

double

AnnualNetworkBreakage;

public

double

AnnualWaterLoss_Breakage_MG;

/**

* Later may change based on literature review

*/

public

double

LeakAmount_g_km_year;

public

double

TotalLeakAmount;

public

int

YearCounter;

// Collection Variables

/**

* Stock

*/

public

ArrayList <

Double > ConditionGroups = new ArrayList<Double>();

public

ArrayList <

Integer > ConditionGroupsYears = new ArrayList<Integer>();

public

ArrayList <

Double > CGLambdas = new ArrayList<Double>();

public

ArrayList <

Double > CG_PVC = new ArrayList<Double>();

public

ArrayList <

Double > CG_CI = new ArrayList<Double>();

public

ArrayList <

Double > Lambda_Break_PVC = new ArrayList<Double>();

public

ArrayList <

Double > Lambda_Break_CI = new ArrayList<Double>();

public

ArrayList <

Double > Leak_PVC = new ArrayList<Double>();

public

ArrayList <

Double > Leak_CI = new ArrayList<Double>();

public

ArrayList <

Double > FiveYearBreakSummary = new ArrayList<Double>();

// Dynamic (Flow/Auxiliary/Stock) Variables

public double LeakageAmount;

public double AnnualDemand;

public double ABreaks;

public double TotalPVC;

public double TotalCI;

public double AnnualTotalWaterLoss;

public double AnnualEnergyLoss_Mwh;

@AnyLogicInternalCodegenAPI

public void assignInitialConditions_xjal() {

super.assignInitialConditions_xjal();

_assign_AnnualDemand_Formula_xjal();

_assign_LeakageAmount_Formula_xjal();

_assign_AnnualTotalWaterLoss_Formula_xjal();

_assign_ABreaks_Formula_xjal();

_assign_TotalPVC_Formula_xjal();

_assign_TotalCI_Formula_xjal();

_assign_AnnualEnergyLoss_Mwh_Formula_xjal();

}

@AnyLogicInternalCodegenAPI

public void setupInitialConditions_xjal(Class<?> callerClass) {

if (callerClass != Pipe.class) {

return;

}

if (getInitialAlgebraicFlatEquationsCount_xjal() > 0) {

SDIntegrationManager integrationManagerForInitialConditions = new SDIntegrationManager( 0, getInitialAlgebraicFlatEquationsCount_xjal(), getInitialFormulaFlatEquationsCount_xjal() );

integrationManagerForInitialConditions.doStep( this, 0, 0.1, true );

} else {

assignInitialConditions_xjal();

}

}

@AnyLogicInternalCodegenAPI

public void _assign_AnnualDemand_Formula_xjal() {

AnnualDemand =

main.user.getAnnualDemand()

;

}

@AnyLogicInternalCodegenAPI

public void _assign_LeakageAmount_Formula_xjal() {

LeakageAmount =

(TotalLeakagePercentage() / 100) * AnnualDemand

;

}

@AnyLogicInternalCodegenAPI

public void _assign_AnnualTotalWaterLoss_Formula_xjal() {

AnnualTotalWaterLoss =

(LeakageAmount / 1000000) + AnnualWaterLoss_Breakage_MG

;

}

@AnyLogicInternalCodegenAPI

public void _assign_ABreaks_Formula_xjal() {

ABreaks =

AnnualBreaks(AverageCondition())

;

}

@AnyLogicInternalCodegenAPI

public void _assign_TotalPVC_Formula_xjal() {

TotalPVC =

TotalPVCPercentage()

;

}

@AnyLogicInternalCodegenAPI

public void _assign_TotalCI_Formula_xjal() {

TotalCI =

TotalCIPercentage()

;

}

@AnyLogicInternalCodegenAPI

public void _assign_AnnualEnergyLoss_Mwh_Formula_xjal() {

AnnualEnergyLoss_Mwh =

main.EnergyIntensityWater * 365 * AnnualTotalWaterLoss

;

}

@AnyLogicInternalCodegenAPI

public void formulasExecute_xjal() {

super.formulasExecute_xjal();

_assign_AnnualDemand_Formula_xjal();

_assign_LeakageAmount_Formula_xjal();

_assign_AnnualTotalWaterLoss_Formula_xjal();

_assign_ABreaks_Formula_xjal();

_assign_TotalPVC_Formula_xjal();

_assign_TotalCI_Formula_xjal();

_assign_AnnualEnergyLoss_Mwh_Formula_xjal();

}

@AnyLogicInternalCodegenAPI

protected SDIntegrationManager integrationManager_xjal = null;

@AnyLogicInternalCodegenAPI

public SDIntegrationManager getIntegrationManager_xjal() {

if (integrationManager_xjal == null) {

integrationManager_xjal = new SDIntegrationManager( getDifferentialFlatEquationsCount_xjal(), getRuntimeAlgebraicFlatEquationsCount_xjal(), getRuntimeFormulaFlatEquationsCount_xjal() );

}

return integrationManager_xjal;

}

@Override

@AnyLogicInternalCodegenAPI

public int getRuntimeFormulaFlatEquationsCount_xjal() {

return super.getRuntimeFormulaFlatEquationsCount_xjal() + 7;

}

@Override

@AnyLogicInternalCodegenAPI

public int getInitialFormulaFlatEquationsCount_xjal() {

return super.getInitialFormulaFlatEquationsCount_xjal() + 7;

}

@AnyLogicInternalCodegenAPI

private static Map<String, IElementDescriptor> elementDesciptors_xjal = null;

@AnyLogicInternalCodegenAPI

@Override

public Map<String, IElementDescriptor> getElementDesciptors() {

if (elementDesciptors_xjal == null) {

elementDesciptors_xjal = createElementDescriptors(super.getElementDesciptors(), Pipe.class);

}

return elementDesciptors_xjal;

}

@AnyLogicCustomProposalPriority(type = AnyLogicCustomProposalPriority.Type.STATIC_ELEMENT)

public static final Scale scale = new Scale( 10.0 );

@Override

public Scale getScale() {

return scale;

}

// Events

public EventTimeout Deterioration = new EventTimeout(this);

public EventTimeout Leakage = new EventTimeout(this);

public EventTimeout Breakage = new EventTimeout(this);

public EventTimeout BreakageFiveYearEvent = new EventTimeout(this);

@AnyLogicInternalCodegenAPI

public EventTimeout _autoCreatedDS_xjal = new EventTimeout(this);

@Override

@AnyLogicInternalCodegenAPI

public String getNameOf( EventTimeout _e ) {

if( _e == Deterioration ) return "Deterioration";

if( _e == Leakage ) return "Leakage";

if( _e == Breakage ) return "Breakage";

if( _e == BreakageFiveYearEvent ) return "BreakageFiveYearEvent";

if( _e == _autoCreatedDS_xjal ) return "Auto-created DataSets auto update event";

return super.getNameOf( _e );

}

@Override

@AnyLogicInternalCodegenAPI

public EventTimeout.Mode getModeOf( EventTimeout _e ) {

if ( _e == Deterioration ) return EVENT_TIMEOUT_MODE_CYCLIC;

if ( _e == Leakage ) return EVENT_TIMEOUT_MODE_CYCLIC;

if ( _e == Breakage ) return EVENT_TIMEOUT_MODE_CYCLIC;

if ( _e == BreakageFiveYearEvent ) return EVENT_TIMEOUT_MODE_CYCLIC;

if ( _e == _autoCreatedDS_xjal ) return EVENT_TIMEOUT_MODE_CYCLIC;

return super.getModeOf( _e );

}

@Override

@AnyLogicInternalCodegenAPI

public double getFirstOccurrenceTime( EventTimeout _e ) {

double _t;

if ( _e == Deterioration ) { return getEngine().dateToTime( toDate( 2017, JANUARY, 2, 10, 0, 0 ) ); }

if ( _e == Leakage ) {

_t =

1

;

_t = toModelTime( _t, YEAR );

return _t;

}

if ( _e == Breakage ) {

_t =

0

;

_t = toModelTime( _t, YEAR );

return _t;

}

if ( _e == BreakageFiveYearEvent ) {

_t =

0

;

_t = toModelTime( _t, YEAR );

return _t;

}

if ( _e == _autoCreatedDS_xjal ) {

_t =

0

;

_t = toModelTime( _t, YEAR );

return _t;

}

return super.getFirstOccurrenceTime( _e );

}

@Override

@AnyLogicInternalCodegenAPI

public double evaluateTimeoutOf( EventTimeout _e ) {

double _t;

if( _e == Deterioration) {

_t =

1

;

_t = toTimeoutInCalendar( YEAR, _t );

return _t;

}

if( _e == Leakage) {

_t =

1

;

_t = toModelTime( _t, YEAR );

return _t;

}

if( _e == Breakage) {

_t =

1

;

_t = toModelTime( _t, YEAR );

return _t;

}

if( _e == BreakageFiveYearEvent) {

_t =

1

;

_t = toModelTime( _t, YEAR );

return _t;

}

if( _e == _autoCreatedDS_xjal) {

_t =

1

;

_t = toModelTime( _t, YEAR );

return _t;

}

return super.evaluateTimeoutOf( _e );

}

@Override

@AnyLogicInternalCodegenAPI

public void executeActionOf( EventTimeout _e ) {

if ( _e == Deterioration ) {

EventTimeout self = _e;

HashMap<Integer,Double> hmap = main.agency.renewal();

double renewal_length100 = hmap.get(100);

double renewal_length80 = hmap.get(80);

material_deterioration(renewal_length100, renewal_length80);

//Deterioration function

for(int i = ConditionGroups.size()-1; i >= 1 ; i--){

double temp = ConditionGroups.get(i);

ConditionGroups.set(i, ConditionGroups.get(i) + ConditionGroups.get(i-1)/20.0);

if(i != ConditionGroups.size()-1){

ConditionGroups.set(i, ConditionGroups.get(i) - temp/20);

}

}

ConditionGroups.set(0, ConditionGroups.get(0) - ConditionGroups.get(0)/20.0);

/*Do renewal for CG 100*/

ConditionGroups.set(0, ConditionGroups.get(0) + renewal_length100);

ConditionGroups.set(4, ConditionGroups.get(4) - renewal_length100);

/*Do renewal for CG 80*/

ConditionGroups.set(0, ConditionGroups.get(0) + renewal_length80);

ConditionGroups.set(3, ConditionGroups.get(3) - renewal_length80);

;

return;

}

if ( _e == Leakage ) {

EventTimeout self = _e;

TotalLeakAmount = 0;

for(int i = 0; i < CGLambdas.size(); i++){

double CGLambda = CGLambdas.get(i);

double GroupLength = ConditionGroups.get(i);

int leakage = poisson(CGLambda);

if(leakage == 1){

//leakage happened

TotalLeakAmount = TotalLeakAmount + GroupLength*LeakAmount_g_km_year;

}

}

;

return;

}

if ( _e == Breakage ) {

EventTimeout self = _e;

int total_breakage = 0;

int index = 0;

for(double ConditionGroupLength : ConditionGroups){

//ConditionGroupLength = floor(ConditionGroupLength);

double lambda_pvc = Lambda_Break_PVC.get(index);

double lambda_ci = Lambda_Break_CI.get(index);

double pvc_length = floor(ConditionGroupLength*(CG_PVC.get(index)/100));

double ci_length = floor(ConditionGroupLength*(CG_CI.get(index)/100));

//for PVC length

while(pvc_length > 0){

//System.out.println("pvc_length: "+pvc_length);

if(poisson(lambda_pvc) == 1){

total_breakage = total_breakage + 1;

}

pvc_length = pvc_length-1;

}

//for ci length

while(ci_length > 0){

//System.out.println("ci_length: "+ci_length);

if(poisson(lambda_ci) == 1){

total_breakage = total_breakage + 1;

}

ci_length = ci_length-1;

}

index = index + 1;

}

//System.out.println("total_breakage: "+total_breakage);

AnnualNetworkBreakage = total_breakage;

double annual_loss = total_breakage*(main.AverageFlowRate*60*24*main.AverageFailureDuration*Math.pow((main.AveragePressure/70),0.5)/1000000);

AnnualWaterLoss_Breakage_MG = total_breakage*annual_loss;

;

return;

}

if ( _e == BreakageFiveYearEvent ) {

EventTimeout self = _e;

YearCounter = YearCounter + 1;

FiveYearBreakSummary.add(AnnualNetworkBreakage);

if(FiveYearBreakSummary.size() > 5){

FiveYearBreakSummary.remove(0);

}

;

return;

}

if ( _e == _autoCreatedDS_xjal ) {

_ds_LeakageAmount.update();

_ds_AnnualDemand.update();

_ds_ABreaks.update();

_ds_TotalPVC.update();

_ds_TotalCI.update();

_ds_AnnualTotalWaterLoss.update();

_ds_AnnualEnergyLoss_Mwh.update();

return;

}

super.executeActionOf( _e );

}

// Functions

/**

* Function for printing decimal numbers

*/

String

printf( double myvalue ) {

DecimalFormat df = new DecimalFormat("#");

df.setMaximumFractionDigits(2);

return df.format(myvalue);

}

double

TotalLengthPipes( ) {

double sum = 0;

int sum_test = 0;

for(double CG : ConditionGroups){

sum += CG;

sum_test = sum_test + (int)CG;

}

//System.out.println("Total pipe length: "+sum);

//System.out.println("Total pipe length-test: "+sum_test);

return sum;

}

double

AverageCondition( ) {

double avg_condition = 0.0;

for(int i = 0; i< ConditionGroups.size(); i++){

avg_condition += ConditionGroups.get(i)*ConditionGroupsYears.get(i);

}

avg_condition = avg_condition/TotalLengthPipeMile;

return avg_condition;

}

double

CG100( ) {

return ConditionGroups.get(4);

}

double

round( double value, int places ) {

if (places < 0) throw new IllegalArgumentException();

long factor = (long) Math.pow(10, places);

value = value * factor;

long tmp = Math.round(value);

return (double) tmp / factor;

}

void RenewCG100( double renewalLength ) {

double pvc_20 = CG_PVC.get(0)/100;

double cg_20 = ConditionGroups.get(0);

double cg_100 = ConditionGroups.get(4);

double ci_20 = CG_CI.get(0)/100;

CG_PVC.set(0, (renewalLength + pvc_20*cg_20-pvc_20*(cg_20/20))/(cg_20-cg_20/20+renewalLength) * 100);

CG_CI.set(0, (ci_20*cg_20-ci_20*(cg_20/20))/(cg_20-cg_20/20+renewalLength) * 100);

//CG_PVC(4, );

//CG_CI(4, );

ConditionGroups.set(0,ConditionGroups.get(0) + renewalLength);

ConditionGroups.set(4, ConditionGroups.get(4) - renewalLength);

}

double

TotalLeakagePercentage( ) {

double g = (TotalPVCPercentage()*.07 + TotalCIPercentage()*.078)/100;

return 0.00075 * exp(AverageCondition()*g)*TotalLengthPipeMile;

}

double

AnnualBreaks( double AverageCondition ) {

double g = (TotalPVCPercentage()*.07 + TotalCIPercentage()*.078)/100;

return 0.01 * exp(AverageCondition*g)*TotalLengthPipeMile;

}

double

TotalPVCPercentage( ) {

double sum=0;

for(int i = 0; i < ConditionGroups.size(); i++){

sum = sum + ConditionGroups.get(i)*CG_PVC.get(i);

}

return sum/TotalLengthPipeMile;

}

double

TotalCIPercentage( ) {

double sum=0;

for(int i = 0; i < ConditionGroups.size(); i++){

sum = sum + ConditionGroups.get(i)*CG_CI.get(i);

}

return sum/TotalLengthPipeMile;

}

/**

* Function for printing decimal numbers

*/

String

printf_int( double myvalue ) {

DecimalFormat df = new DecimalFormat("#");

df.setMaximumFractionDigits(0);

return df.format(myvalue);

}

void material_deterioration( double renewal100, double renewal80 ) {

//System.out.println(getYear()+") renewal100 length: "+renewal100+" renewal80 length: "+renewal80);

/** Deterioration of group 20 **/

double pvc20_percentage = CG_PVC.get(0)/100;

double ci20_percentage = CG_CI.get(0)/100;

double total20 = ConditionGroups.get(0);

double l20pvc = pvc20_percentage*total20-0.05*pvc20_percentage*total20;

double l20ci = ci20_percentage*total20-0.05*ci20_percentage*total20;

double l20pvc_plus_renew = l20pvc + renewal100 + renewal80; //renewal80 added

CG_PVC.set(0, (l20pvc_plus_renew/(l20pvc_plus_renew+l20ci))*100);

CG_CI.set(0, (l20ci/(l20pvc_plus_renew+l20ci))*100);

/** Change in group 40 **/

double pvc40_percentage = CG_PVC.get(1)/100;

double ci40_percentage = CG_CI.get(1)/100;

double total40 = ConditionGroups.get(1);

double l40pvc = pvc40_percentage*total40-0.05*pvc40_percentage*total40 + 0.05*pvc20_percentage*total20;

double l40ci = ci40_percentage*total40-0.05*ci40_percentage*total40 + 0.05*ci20_percentage*total20;

CG_PVC.set(1, (l40pvc/(l40pvc+l40ci))*100);

CG_CI.set(1, (l40ci/(l40pvc+l40ci))*100);

/** Change in group 60 **/

double pvc60_percentage = CG_PVC.get(2)/100;

double ci60_percentage = CG_CI.get(2)/100;

double total60 = ConditionGroups.get(2);

double l60pvc = pvc60_percentage*total60-0.05*pvc60_percentage*total60 + 0.05*pvc40_percentage*total40;

double l60ci = ci60_percentage*total60-0.05*ci60_percentage*total60 + 0.05*ci40_percentage*total40;

CG_PVC.set(2, (l60pvc/(l60pvc+l60ci))*100);

CG_CI.set(2, (l60ci/(l60pvc+l60ci))*100);

/** Change in group 80 **/

double pvc80_percentage = CG_PVC.get(3)/100;

double ci80_percentage = CG_CI.get(3)/100;

double total80 = ConditionGroups.get(3);

double l80pvc = pvc80_percentage*total80-0.05*pvc80_percentage*total80 + 0.05*pvc60_percentage*total60;

double l80ci = ci80_percentage*total80-0.05*ci80_percentage*total80 + 0.05*ci60_percentage*total60;

//renewal80 added

l80pvc = l80pvc - pvc80_percentage*renewal80;

l80ci = l80ci - ci80_percentage*renewal80;

CG_PVC.set(3, (l80pvc/(l80pvc+l80ci))*100);

CG_CI.set(3, (l80ci/(l80pvc+l80ci))*100);

/** Change in group 100 **/

double pvc100_percentage = CG_PVC.get(4)/100;

double ci100_percentage = CG_CI.get(4)/100;

double total100 = ConditionGroups.get(4);

double l100pvc = pvc100_percentage*total100-pvc100_percentage*renewal100 + 0.05*pvc80_percentage*total80;

double l100ci = ci100_percentage*total100-ci100_percentage*renewal100 + 0.05*ci80_percentage*total80;

CG_PVC.set(4, (l100pvc/(l100pvc+l100ci))*100);

CG_CI.set(4, (l100ci/(l100pvc+l100ci))*100);

}

HashMap<Integer, Double>

CalculateL80PercentageRA( HashMap<Integer, Double> hmap, double budgetRenewalLimit ) {

//from 0.1 to 1 +0.1

double percentage = hmap.get(100)/ConditionGroups.get(4);

ArrayList<Double> condition_groups = new ArrayList<>(5);

double cg80 = ConditionGroups.get(3);

double cg100 = ConditionGroups.get(4);

condition_groups.add(ConditionGroups.get(0));

condition_groups.add(ConditionGroups.get(1));

condition_groups.add(ConditionGroups.get(2));

condition_groups.add(cg80);

condition_groups.add(ConditionGroups.get(4) - hmap.get(100));

double totalBreakage = CalculateBreakage(condition_groups);

while(totalBreakage > main.BreakToleranceChoice && percentage < 1

&& cg100*percentage < budgetRenewalLimit){

percentage = percentage + 0.1; //increase percentage

condition_groups.set(4, cg100 - cg100*percentage);

totalBreakage = CalculateBreakage(condition_groups);

}

if(percentage > 1){

percentage = 1;

}

hmap.put(100, cg100*percentage);

System.out.println("100 percentage: "+percentage*100);

budgetRenewalLimit = budgetRenewalLimit - cg100*percentage;

percentage = 0;

//if the total breakage tolerance has not been satisfied yet and there is still budget

//renew group condition 80

while(totalBreakage > main.BreakToleranceChoice && percentage < 1

&& cg80*percentage < budgetRenewalLimit){

percentage = percentage + 0.1; //increase percentage

condition_groups.set(3, cg80 - cg80*percentage);

totalBreakage = CalculateBreakage(condition_groups);

}

if(percentage > 1){

percentage = 1;

}

hmap.put(80, cg80*percentage);

System.out.println("80 percentage: "+percentage*100);

System.out.println(getYear()+", "+hmap.toString());

return hmap;

}

double

CalculateBreakage( ArrayList<Double> condition_groups ) {

int total_breakage = 0;

int index = 0;

for(double ConditionGroupLength : condition_groups){

double lambda_pvc = Lambda_Break_PVC.get(index);

double lambda_ci = Lambda_Break_CI.get(index);

double pvc_length = floor(ConditionGroupLength*(CG_PVC.get(index)/100));

double ci_length = floor(ConditionGroupLength*(CG_CI.get(index)/100));

//for PVC length

while(pvc_length > 0){

//System.out.println("pvc_length: "+pvc_length);

if(poisson(lambda_pvc) == 1){

total_breakage = total_breakage + 1;

}

pvc_length = pvc_length-1;

}

//for ci length

while(ci_length > 0){

//System.out.println("ci_length: "+ci_length);

if(poisson(lambda_ci) == 1){

total_breakage = total_breakage + 1;

}

ci_length = ci_length-1;

}

index = index + 1;

}

double count = 5.0;

double sum = total_breakage;

for(int i = 1; i < count; i++){

sum = FiveYearBreakSummary.get(i) + sum;

}

return sum/count;

}

double

CG80( ) {

return ConditionGroups.get(3);

}

HashMap<Integer, Double>

CalculateL80PercentageRN( HashMap<Integer, Double> hmap, double budgetRenewalLimit ) {

//from 0.1 to 1 +0.1

//while(CalculateAverageCondition(condition_groups) >= main.DesiredAverageConditionChoice && percentage<= 1 && ConditionGroups.get(3)*percentage < budgetRenewalLimit){

double percentage = hmap.get(100)/ConditionGroups.get(4);

ArrayList<Double> condition_groups = new ArrayList<>(5);

double cg80 = ConditionGroups.get(3);

double cg100 = ConditionGroups.get(4);

condition_groups.add(ConditionGroups.get(0));

condition_groups.add(ConditionGroups.get(1));

condition_groups.add(ConditionGroups.get(2));

condition_groups.add(cg80);

condition_groups.add(ConditionGroups.get(4) - hmap.get(100));

double averageCondition = CalculateAverageCondition(condition_groups);

while(averageCondition > main.BreakToleranceChoice && percentage < 1

&& cg100*percentage < budgetRenewalLimit){

percentage = percentage + 0.1; //increase percentage

condition_groups.set(4, cg100 - cg100*percentage);

averageCondition = CalculateAverageCondition(condition_groups);

}

if(percentage > 1){

percentage = 1;

}

hmap.put(100, cg100*percentage);

System.out.println("100 percentage: "+percentage*100);

budgetRenewalLimit = budgetRenewalLimit - cg100*percentage;

percentage = 0;

//if the total breakage tolerance has not been satisfied yet and there is still budget

//renew group condition 80

while(averageCondition > main.BreakToleranceChoice && percentage < 1

&& cg80*percentage < budgetRenewalLimit){

percentage = percentage + 0.1; //increase percentage

condition_groups.set(3, cg80 - cg80*percentage);

averageCondition = CalculateAverageCondition(condition_groups);

}

if(percentage > 1){

percentage = 1;

}

hmap.put(80, cg80*percentage);

System.out.println("80 percentage: "+percentage*100);

System.out.println(getYear()+", "+hmap.toString());

return hmap;

}

double

CalculateAverageCondition( ArrayList<Double> condition_groups ) {

double avg_condition = 0.0;

for(int i = 0; i< condition_groups.size(); i++){

avg_condition += condition_groups.get(i)*ConditionGroupsYears.get(i);

}

avg_condition = avg_condition/TotalLengthPipeMile;

return avg_condition;

}

double

AverageFiveYearBreakage( ) {

double count = 0.0;

double sum = 0.0;

if(FiveYearBreakSummary.size() == 5){

for(int i = 0; i < 5; i++){

sum = FiveYearBreakSummary.get(i) + sum;

count= count + 1;

}

return sum/count;

}

else{

return 0.0;

}

}

/**

* Auto-created data set(s) for LeakageAmount

*/

@AnyLogicInternalCodegenAPI

public DataSet _ds_LeakageAmount = new DataSet( 100, new DataUpdater_xjal() {

double _lastUpdateTime = Double.NaN;

@Override

public void update( DataSet _d ) {

if ( time() == _lastUpdateTime ) { return; }

_d.add( time(), Pipe.this.LeakageAmount );

_lastUpdateTime = time();

}

} );

/**

* Auto-created data set(s) for AnnualDemand

*/

@AnyLogicInternalCodegenAPI

public DataSet _ds_AnnualDemand = new DataSet( 100, new DataUpdater_xjal() {

double _lastUpdateTime = Double.NaN;

@Override

public void update( DataSet _d ) {

if ( time() == _lastUpdateTime ) { return; }

_d.add( time(), Pipe.this.AnnualDemand );

_lastUpdateTime = time();

}

} );

/**

* Auto-created data set(s) for ABreaks

*/

@AnyLogicInternalCodegenAPI

public DataSet _ds_ABreaks = new DataSet( 100, new DataUpdater_xjal() {

double _lastUpdateTime = Double.NaN;

@Override

public void update( DataSet _d ) {

if ( time() == _lastUpdateTime ) { return; }

_d.add( time(), Pipe.this.ABreaks );

_lastUpdateTime = time();

}

} );

/**

* Auto-created data set(s) for TotalPVC

*/

@AnyLogicInternalCodegenAPI

public DataSet _ds_TotalPVC = new DataSet( 100, new DataUpdater_xjal() {

double _lastUpdateTime = Double.NaN;

@Override

public void update( DataSet _d ) {

if ( time() == _lastUpdateTime ) { return; }

_d.add( time(), Pipe.this.TotalPVC );

_lastUpdateTime = time();

}

} );

/**

* Auto-created data set(s) for TotalCI

*/

@AnyLogicInternalCodegenAPI

public DataSet _ds_TotalCI = new DataSet( 100, new DataUpdater_xjal() {

double _lastUpdateTime = Double.NaN;

@Override

public void update( DataSet _d ) {

if ( time() == _lastUpdateTime ) { return; }

_d.add( time(), Pipe.this.TotalCI );

_lastUpdateTime = time();

}

} );

/**

* Auto-created data set(s) for AnnualTotalWaterLoss

*/

@AnyLogicInternalCodegenAPI

public DataSet _ds_AnnualTotalWaterLoss = new DataSet( 100, new DataUpdater_xjal() {

double _lastUpdateTime = Double.NaN;

@Override

public void update( DataSet _d ) {

if ( time() == _lastUpdateTime ) { return; }

_d.add( time(), Pipe.this.AnnualTotalWaterLoss );

_lastUpdateTime = time();

}

} );

/**

* Auto-created data set(s) for AnnualEnergyLoss_Mwh

*/

@AnyLogicInternalCodegenAPI

public DataSet _ds_AnnualEnergyLoss_Mwh = new DataSet( 100, new DataUpdater_xjal() {

double _lastUpdateTime = Double.NaN;

@Override

public void update( DataSet _d ) {

if ( time() == _lastUpdateTime ) { return; }

_d.add( time(), Pipe.this.AnnualEnergyLoss_Mwh );

_lastUpdateTime = time();

}

} );

// View areas

public ViewArea _origin_VA = new ViewArea( this, "[Origin]", 0, 0, 1000.0, 590.0 );

@Override

@AnyLogicInternalCodegenAPI

public int getViewAreas(Map<String, ViewArea> _output) {

if ( _output != null ) {

_output.put( "_origin_VA", this._origin_VA );

}

return 1 + super.getViewAreas( _output );

}

@AnyLogicInternalCodegenAPI

protected static final Font _text_Font = new Font("SansSerif", 0, 10 );

@AnyLogicInternalCodegenAPI

protected static final Font _text1_Font = _text_Font;

@AnyLogicInternalCodegenAPI

protected static final Font _text2_Font = _text_Font;

@AnyLogicInternalCodegenAPI

protected static final Font _text3_Font = _text_Font;

@AnyLogicInternalCodegenAPI

protected static final Font _text4_Font = _text_Font;

@AnyLogicInternalCodegenAPI

protected static final Font _text5_Font = _text_Font;

@AnyLogicInternalCodegenAPI

protected static final Font _text6_Font = _text_Font;

@AnyLogicInternalCodegenAPI

protected static final Font _text7_Font = _text_Font;

@AnyLogicInternalCodegenAPI

protected static final Font _text8_Font = _text_Font;

@AnyLogicInternalCodegenAPI

protected static final Font _text9_Font = _text_Font;

@AnyLogicInternalCodegenAPI

protected static final Font _text11_Font = new Font("SansSerif", 0, 12 );

@AnyLogicInternalCodegenAPI

protected static final Font _text12_Font = new Font("SansSerif", 0, 11 );

@AnyLogicInternalCodegenAPI

protected static final Font _text10_Font = _text12_Font;

@AnyLogicInternalCodegenAPI

protected static final Font _text13_Font = _text12_Font;

@AnyLogicInternalCodegenAPI

protected static final Font _text14_Font = _text12_Font;

@AnyLogicInternalCodegenAPI

protected static final Font _text15_Font = _text12_Font;

@AnyLogicInternalCodegenAPI

protected static final Font _text16_Font = _text12_Font;

@AnyLogicInternalCodegenAPI

protected static final Font _text17_Font = _text12_Font;

@AnyLogicInternalCodegenAPI

protected static final Font _text18_Font = _text12_Font;

@AnyLogicInternalCodegenAPI

protected static final Font _text19_Font = _text12_Font;

@AnyLogicInternalCodegenAPI

protected static final Font _text20_Font = _text12_Font;

@AnyLogicInternalCodegenAPI

protected static final Font _text21_Font = _text12_Font;

@AnyLogicInternalCodegenAPI

protected static final Font _text22_Font = _text12_Font;

@AnyLogicInternalCodegenAPI

protected static final Font _text23_Font = _text12_Font;

@AnyLogicInternalCodegenAPI

protected static final int _rectangle = 1;

@AnyLogicInternalCodegenAPI

protected static final int _rectangle1 = 2;

@AnyLogicInternalCodegenAPI

protected static final int _text = 3;

@AnyLogicInternalCodegenAPI

protected static final int _text1 = 4;

@AnyLogicInternalCodegenAPI

protected static final int _text2 = 5;

@AnyLogicInternalCodegenAPI

protected static final int _text3 = 6;

@AnyLogicInternalCodegenAPI

protected static final int _rectangle2 = 7;

@AnyLogicInternalCodegenAPI

protected static final int _text4 = 8;

@AnyLogicInternalCodegenAPI

protected static final int _text5 = 9;

@AnyLogicInternalCodegenAPI

protected static final int _rectangle3 = 10;

@AnyLogicInternalCodegenAPI

protected static final int _text6 = 11;

@AnyLogicInternalCodegenAPI

protected static final int _text7 = 12;

@AnyLogicInternalCodegenAPI

protected static final int _rectangle4 = 13;

@AnyLogicInternalCodegenAPI

protected static final int _text8 = 14;

@AnyLogicInternalCodegenAPI

protected static final int _text9 = 15;

@AnyLogicInternalCodegenAPI

protected static final int _line = 16;

@AnyLogicInternalCodegenAPI

protected static final int _line2 = 17;

@AnyLogicInternalCodegenAPI

protected static final int _line3 = 18;

@AnyLogicInternalCodegenAPI

protected static final int _line4 = 19;

@AnyLogicInternalCodegenAPI

protected static final int _line5 = 20;

@AnyLogicInternalCodegenAPI

protected static final int _text11 = 21;

@AnyLogicInternalCodegenAPI

protected static final int _text12 = 22;

@AnyLogicInternalCodegenAPI

protected static final int _rectangle5 = 23;

@AnyLogicInternalCodegenAPI

protected static final int _text10 = 24;

@AnyLogicInternalCodegenAPI

protected static final int _line1 = 25;

@AnyLogicInternalCodegenAPI

protected static final int _rectangle6 = 26;

@AnyLogicInternalCodegenAPI

protected static final int _text13 = 27;

@AnyLogicInternalCodegenAPI

protected static final int _line6 = 28;

@AnyLogicInternalCodegenAPI

protected static final int _rectangle7 = 29;

@AnyLogicInternalCodegenAPI

protected static final int _text14 = 30;

@AnyLogicInternalCodegenAPI

protected static final int _line7 = 31;

@AnyLogicInternalCodegenAPI

protected static final int _rectangle8 = 32;

@AnyLogicInternalCodegenAPI

protected static final int _text15 = 33;

@AnyLogicInternalCodegenAPI

protected static final int _line8 = 34;

@AnyLogicInternalCodegenAPI

protected static final int _rectangle9 = 35;

@AnyLogicInternalCodegenAPI

protected static final int _text16 = 36;

@AnyLogicInternalCodegenAPI

protected static final int _line9 = 37;

@AnyLogicInternalCodegenAPI

protected static final int _text17 = 38;

@AnyLogicInternalCodegenAPI

protected static final int _text18 = 39;

@AnyLogicInternalCodegenAPI

protected static final int _text19 = 40;

@AnyLogicInternalCodegenAPI

protected static final int _text20 = 41;

@AnyLogicInternalCodegenAPI

protected static final int _text21 = 42;

@AnyLogicInternalCodegenAPI

protected static final int _text22 = 43;

@AnyLogicInternalCodegenAPI

protected static final int _text23 = 44;

/** Internal constant, shouldn't be accessed by user */

@AnyLogicInternalCodegenAPI

protected static final int _SHAPE_NEXT_ID_xjal = 45;

/**

* Top-level presentation group id

*/

@AnyLogicInternalCodegenAPI

protected static final int _presentation = 0;

@AnyLogicInternalCodegenAPI

public boolean isPublicPresentationDefined() {

return true;

}

@AnyLogicInternalCodegenAPI

public boolean isEmbeddedAgentPresentationVisible( Agent _a ) {

return super.isEmbeddedAgentPresentationVisible( _a );

}

/**

* Top-level icon group id

*/

@AnyLogicInternalCodegenAPI

protected static final int _icon = -1;

protected ShapeRectangle rectangle;

protected ShapeRectangle rectangle1;

protected ShapeText text;

/**

* <i>This method should not be called by user</i>

*/

@AnyLogicInternalCodegenAPI

private void _text1_SetDynamicParams_xjal( ShapeText shape ) {

shape.setText(

printf(ConditionGroups.get(0))+" miles"

);

}

protected ShapeText text1;

protected ShapeText text2;

/**

* <i>This method should not be called by user</i>

*/

@AnyLogicInternalCodegenAPI

private void _text3_SetDynamicParams_xjal( ShapeText shape ) {

shape.setText(

printf(ConditionGroups.get(1))+" miles"

);

}

protected ShapeText text3;

protected ShapeRectangle rectangle2;

protected ShapeText text4;

/**

* <i>This method should not be called by user</i>

*/

@AnyLogicInternalCodegenAPI

private void _text5_SetDynamicParams_xjal( ShapeText shape ) {

shape.setText(

printf(ConditionGroups.get(2))+" miles"

);

}

protected ShapeText text5;

protected ShapeRectangle rectangle3;

protected ShapeText text6;

/**

* <i>This method should not be called by user</i>

*/

@AnyLogicInternalCodegenAPI

private void _text7_SetDynamicParams_xjal( ShapeText shape ) {

shape.setText(

printf(ConditionGroups.get(3))+" miles"

);

}

protected ShapeText text7;

protected ShapeRectangle rectangle4;

protected ShapeText text8;

/**

* <i>This method should not be called by user</i>

*/

@AnyLogicInternalCodegenAPI

private void _text9_SetDynamicParams_xjal( ShapeText shape ) {

shape.setText(

printf(ConditionGroups.get(4))+" miles"

);

}

protected ShapeText text9;

protected ShapeLine line;

protected ShapeLine line2;

protected ShapeLine line3;

protected ShapeLine line4;

protected ShapeLine line5;

/**

* <i>This method should not be called by user</i>

*/

@AnyLogicInternalCodegenAPI

private void _text11_SetDynamicParams_xjal( ShapeText shape ) {

shape.setText(

"Average Condition: "+printf(AverageCondition())

);

}

protected ShapeText text11;

/**

* <i>This method should not be called by user</i>

*/

@AnyLogicInternalCodegenAPI

private void _text12_SetDynamicParams_xjal( ShapeText shape ) {

shape.setText(

"Renewal: "+printf(CG100())

);

}

protected ShapeText text12;

protected ShapeRectangle rectangle5;

/**

* <i>This method should not be called by user</i>

*/

@AnyLogicInternalCodegenAPI

private void _text10_SetDynamicParams_xjal( ShapeText shape ) {

shape.setText(

printf_int(CG_PVC.get(0))+"%"

);

}

protected ShapeText text10;

protected ShapeLine line1;

protected ShapeRectangle rectangle6;

/**

* <i>This method should not be called by user</i>

*/

@AnyLogicInternalCodegenAPI

private void _text13_SetDynamicParams_xjal( ShapeText shape ) {

shape.setText(

printf_int(CG_PVC.get(1))+"%"

);

}

protected ShapeText text13;

protected ShapeLine line6;

protected ShapeRectangle rectangle7;

/**

* <i>This method should not be called by user</i>

*/

@AnyLogicInternalCodegenAPI

private void _text14_SetDynamicParams_xjal( ShapeText shape ) {

shape.setText(

printf_int(CG_PVC.get(2))+"%"

);

}

protected ShapeText text14;

protected ShapeLine line7;

protected ShapeRectangle rectangle8;

/**

* <i>This method should not be called by user</i>

*/

@AnyLogicInternalCodegenAPI

private void _text15_SetDynamicParams_xjal( ShapeText shape ) {

shape.setText(

printf_int(CG_PVC.get(3))+"%"

);

}

protected ShapeText text15;

protected ShapeLine line8;

protected ShapeRectangle rectangle9;

/**

* <i>This method should not be called by user</i>

*/

@AnyLogicInternalCodegenAPI

private void _text16_SetDynamicParams_xjal( ShapeText shape ) {

shape.setText(

printf_int(CG_PVC.get(4))+"%"

);

}

protected ShapeText text16;

protected ShapeLine line9;

protected ShapeText text17;

/**

* <i>This method should not be called by user</i>

*/

@AnyLogicInternalCodegenAPI

private void _text18_SetDynamicParams_xjal( ShapeText shape ) {

shape.setText(

printf_int(CG_CI.get(0))+"%"

);

}

protected ShapeText text18;

/**

* <i>This method should not be called by user</i>

*/

@AnyLogicInternalCodegenAPI

private void _text19_SetDynamicParams_xjal( ShapeText shape ) {

shape.setText(

printf_int(CG_CI.get(1))+"%"

);

}

protected ShapeText text19;

/**

* <i>This method should not be called by user</i>

*/

@AnyLogicInternalCodegenAPI

private void _text20_SetDynamicParams_xjal( ShapeText shape ) {

shape.setText(

printf_int(CG_CI.get(2))+"%"

);

}

protected ShapeText text20;

/**

* <i>This method should not be called by user</i>

*/

@AnyLogicInternalCodegenAPI

private void _text21_SetDynamicParams_xjal( ShapeText shape ) {

shape.setText(

printf_int(CG_CI.get(3))+"%"

);

}

protected ShapeText text21;

/**

* <i>This method should not be called by user</i>

*/

@AnyLogicInternalCodegenAPI

private void _text22_SetDynamicParams_xjal( ShapeText shape ) {

shape.setText(

printf_int(CG_CI.get(4))+"%"

);

}

protected ShapeText text22;

protected ShapeText text23;

@AnyLogicInternalCodegenAPI

private void _createPersistentElementsBP0_xjal() {

rectangle = new ShapeRectangle(

SHAPE_DRAW_2D, true,60.0, 140.0, 0.0, 0.0,

black, white,

80.0, 60.0, 10.0, 1.0, LINE_STYLE_SOLID );

rectangle1 = new ShapeRectangle(

SHAPE_DRAW_2D, true,180.0, 140.0, 0.0, 0.0,

black, white,

80.0, 60.0, 10.0, 1.0, LINE_STYLE_SOLID );

text = new ShapeText(

SHAPE_DRAW_2D, true,70.0, 140.0, 0.0, 0.0,

black,"Condition\r\nGroup 20",

_text_Font, ALIGNMENT_LEFT );

text1 = new ShapeText(

SHAPE_DRAW_2D, true,70.0, 180.0, 0.0, 0.0,

black,"",

_text1_Font, ALIGNMENT_LEFT ) {

@Override

public void updateDynamicProperties(boolean publicOnly) {

_text1_SetDynamicParams_xjal( this );

super.updateDynamicProperties(publicOnly);

}

};

text2 = new ShapeText(

SHAPE_DRAW_2D, true,190.0, 140.0, 0.0, 0.0,

black,"Condition\r\nGroup 40",

_text2_Font, ALIGNMENT_LEFT );

text3 = new ShapeText(

SHAPE_DRAW_2D, true,190.0, 180.0, 0.0, 0.0,

black,"",

_text3_Font, ALIGNMENT_LEFT ) {

@Override

public void updateDynamicProperties(boolean publicOnly) {

_text3_SetDynamicParams_xjal( this );

super.updateDynamicProperties(publicOnly);

}

};

rectangle2 = new ShapeRectangle(

SHAPE_DRAW_2D, true,300.0, 140.0, 0.0, 0.0,

black, white,

80.0, 60.0, 10.0, 1.0, LINE_STYLE_SOLID );

text4 = new ShapeText(

SHAPE_DRAW_2D, true,310.0, 140.0, 0.0, 0.0,

black,"Condition\r\nGroup 60",

_text4_Font, ALIGNMENT_LEFT );

text5 = new ShapeText(

SHAPE_DRAW_2D, true,310.0, 180.0, 0.0, 0.0,

black,"",

_text5_Font, ALIGNMENT_LEFT ) {

@Override

public void updateDynamicProperties(boolean publicOnly) {

_text5_SetDynamicParams_xjal( this );

super.updateDynamicProperties(publicOnly);

}

};

rectangle3 = new ShapeRectangle(

SHAPE_DRAW_2D, true,420.0, 140.0, 0.0, 0.0,

black, white,

80.0, 60.0, 10.0, 1.0, LINE_STYLE_SOLID );

text6 = new ShapeText(

SHAPE_DRAW_2D, true,430.0, 140.0, 0.0, 0.0,

black,"Condition\r\nGroup 80",

_text6_Font, ALIGNMENT_LEFT );

text7 = new ShapeText(

SHAPE_DRAW_2D, true,430.0, 180.0, 0.0, 0.0,

black,"",

_text7_Font, ALIGNMENT_LEFT ) {

@Override

public void updateDynamicProperties(boolean publicOnly) {

_text7_SetDynamicParams_xjal( this );

super.updateDynamicProperties(publicOnly);

}

};

rectangle4 = new ShapeRectangle(

SHAPE_DRAW_2D, true,540.0, 140.0, 0.0, 0.0,

black, white,

80.0, 60.0, 10.0, 1.0, LINE_STYLE_SOLID );

text8 = new ShapeText(

SHAPE_DRAW_2D, true,550.0, 140.0, 0.0, 0.0,

black,"Condition\r\nGroup 100",

_text8_Font, ALIGNMENT_LEFT );

text9 = new ShapeText(

SHAPE_DRAW_2D, true,550.0, 180.0, 0.0, 0.0,

black,"",

_text9_Font, ALIGNMENT_LEFT ) {

@Override

public void updateDynamicProperties(boolean publicOnly) {

_text9_SetDynamicParams_xjal( this );

super.updateDynamicProperties(publicOnly);

}

};

line = new ShapeLine(

SHAPE_DRAW_2D, true, 60.0, 170.0, 0.0, black,

80.0, 0.0, 0.0, 1.0, 10.0, LINE_STYLE_SOLID );

line2 = new ShapeLine(

SHAPE_DRAW_2D, true, 180.0, 170.0, 0.0, black,

80.0, 0.0, 0.0, 1.0, 10.0, LINE_STYLE_SOLID );

line3 = new ShapeLine(

SHAPE_DRAW_2D, true, 300.0, 170.0, 0.0, black,

80.0, 0.0, 0.0, 1.0, 10.0, LINE_STYLE_SOLID );

line4 = new ShapeLine(

SHAPE_DRAW_2D, true, 420.0, 170.0, 0.0, black,

80.0, 0.0, 0.0, 1.0, 10.0, LINE_STYLE_SOLID );

line5 = new ShapeLine(

SHAPE_DRAW_2D, true, 540.0, 170.0, 0.0, black,

80.0, 0.0, 0.0, 1.0, 10.0, LINE_STYLE_SOLID );

text11 = new ShapeText(

SHAPE_DRAW_2D, true,60.0, 280.0, 0.0, 0.0,

black,"text",

_text11_Font, ALIGNMENT_LEFT ) {

@Override

public void updateDynamicProperties(boolean publicOnly) {

_text11_SetDynamicParams_xjal( this );

super.updateDynamicProperties(publicOnly);

}

};

text12 = new ShapeText(

SHAPE_DRAW_2D, true,-150.0, 150.0, 0.0, 0.0,

dodgerBlue,"text",

_text12_Font, ALIGNMENT_LEFT ) {

@Override

public void updateDynamicProperties(boolean publicOnly) {

_text12_SetDynamicParams_xjal( this );

super.updateDynamicProperties(publicOnly);

}

};

rectangle5 = new ShapeRectangle(

SHAPE_DRAW_2D, true,60.0, 210.0, 0.0, 0.0,

black, white,

80.0, 60.0, 10.0, 1.0, LINE_STYLE_SOLID );

text10 = new ShapeText(

SHAPE_DRAW_2D, true,90.0, 220.0, 0.0, 0.0,

black,"text",

_text10_Font, ALIGNMENT_LEFT ) {

@Override

public void updateDynamicProperties(boolean publicOnly) {

_text10_SetDynamicParams_xjal( this );

super.updateDynamicProperties(publicOnly);

}

};

line1 = new ShapeLine(

SHAPE_DRAW_2D, true, 60.0, 240.0, 0.0, black,

80.0, 0.0, 0.0, 1.0, 10.0, LINE_STYLE_SOLID );

rectangle6 = new ShapeRectangle(

SHAPE_DRAW_2D, true,180.0, 210.0, 0.0, 0.0,

black, white,

80.0, 60.0, 10.0, 1.0, LINE_STYLE_SOLID );

text13 = new ShapeText(

SHAPE_DRAW_2D, true,210.0, 220.0, 0.0, 0.0,

black,"text",

_text13_Font, ALIGNMENT_LEFT ) {

@Override

public void updateDynamicProperties(boolean publicOnly) {

_text13_SetDynamicParams_xjal( this );

super.updateDynamicProperties(publicOnly);

}

};

line6 = new ShapeLine(

SHAPE_DRAW_2D, true, 180.0, 240.0, 0.0, black,

80.0, 0.0, 0.0, 1.0, 10.0, LINE_STYLE_SOLID );

rectangle7 = new ShapeRectangle(

SHAPE_DRAW_2D, true,300.0, 210.0, 0.0, 0.0,

black, white,

80.0, 60.0, 10.0, 1.0, LINE_STYLE_SOLID );

text14 = new ShapeText(

SHAPE_DRAW_2D, true,330.0, 220.0, 0.0, 0.0,

black,"text",

_text14_Font, ALIGNMENT_LEFT ) {

@Override

public void updateDynamicProperties(boolean publicOnly) {

_text14_SetDynamicParams_xjal( this );

super.updateDynamicProperties(publicOnly);

}

};

line7 = new ShapeLine(

SHAPE_DRAW_2D, true, 300.0, 240.0, 0.0, black,

80.0, 0.0, 0.0, 1.0, 10.0, LINE_STYLE_SOLID );

rectangle8 = new ShapeRectangle(

SHAPE_DRAW_2D, true,420.0, 210.0, 0.0, 0.0,

black, white,

80.0, 60.0, 10.0, 1.0, LINE_STYLE_SOLID );

text15 = new ShapeText(

SHAPE_DRAW_2D, true,450.0, 220.0, 0.0, 0.0,

black,"text",

_text15_Font, ALIGNMENT_LEFT ) {

@Override

public void updateDynamicProperties(boolean publicOnly) {

_text15_SetDynamicParams_xjal( this );

super.updateDynamicProperties(publicOnly);

}

};

line8 = new ShapeLine(

SHAPE_DRAW_2D, true, 420.0, 240.0, 0.0, black,

80.0, 0.0, 0.0, 1.0, 10.0, LINE_STYLE_SOLID );

rectangle9 = new ShapeRectangle(

SHAPE_DRAW_2D, true,540.0, 210.0, 0.0, 0.0,

black, white,

80.0, 60.0, 10.0, 1.0, LINE_STYLE_SOLID );

text16 = new ShapeText(

SHAPE_DRAW_2D, true,570.0, 220.0, 0.0, 0.0,

black,"text",

_text16_Font, ALIGNMENT_LEFT ) {

@Override

public void updateDynamicProperties(boolean publicOnly) {

_text16_SetDynamicParams_xjal( this );

super.updateDynamicProperties(publicOnly);

}

};

line9 = new ShapeLine(

SHAPE_DRAW_2D, true, 540.0, 240.0, 0.0, black,

80.0, 0.0, 0.0, 1.0, 10.0, LINE_STYLE_SOLID );

text17 = new ShapeText(

SHAPE_DRAW_2D, true,20.0, 220.0, 0.0, 0.0,

black,"PVC %:",

_text17_Font, ALIGNMENT_LEFT );

text18 = new ShapeText(

SHAPE_DRAW_2D, true,90.0, 250.0, 0.0, 0.0,

black,"text",

_text18_Font, ALIGNMENT_LEFT ) {

@Override

public void updateDynamicProperties(boolean publicOnly) {

_text18_SetDynamicParams_xjal( this );

super.updateDynamicProperties(publicOnly);

}

};

text19 = new ShapeText(

SHAPE_DRAW_2D, true,210.0, 250.0, 0.0, 0.0,

black,"text",

_text19_Font, ALIGNMENT_LEFT ) {

@Override

public void updateDynamicProperties(boolean publicOnly) {

_text19_SetDynamicParams_xjal( this );

super.updateDynamicProperties(publicOnly);

}

};

text20 = new ShapeText(

SHAPE_DRAW_2D, true,330.0, 250.0, 0.0, 0.0,

black,"text",

_text20_Font, ALIGNMENT_LEFT ) {

@Override

public void updateDynamicProperties(boolean publicOnly) {

_text20_SetDynamicParams_xjal( this );

super.updateDynamicProperties(publicOnly);

}

};

text21 = new ShapeText(

SHAPE_DRAW_2D, true,450.0, 250.0, 0.0, 0.0,

black,"text",

_text21_Font, ALIGNMENT_LEFT ) {

@Override

public void updateDynamicProperties(boolean publicOnly) {

_text21_SetDynamicParams_xjal( this );

super.updateDynamicProperties(publicOnly);

}

};

text22 = new ShapeText(

SHAPE_DRAW_2D, true,570.0, 250.0, 0.0, 0.0,

black,"text",

_text22_Font, ALIGNMENT_LEFT ) {

@Override

public void updateDynamicProperties(boolean publicOnly) {

_text22_SetDynamicParams_xjal( this );

super.updateDynamicProperties(publicOnly);

}

};

text23 = new ShapeText(

SHAPE_DRAW_2D, true,20.0, 250.0, 0.0, 0.0,

black,"CI %:",

_text23_Font, ALIGNMENT_LEFT );

}

@AnyLogicInternalCodegenAPI

private void _createPersistentElementsAP0_xjal() {

}

// Static initialization of persistent elements

{

_createPersistentElementsBP0_xjal();

}

protected ShapeTopLevelPresentationGroup presentation;

protected ShapeGroup icon;

@Override

@AnyLogicInternalCodegenAPI

public Object getPersistentShape( int _shape ) {

switch (_shape) {

case _presentation: return presentation;

case _icon: return icon;

case _rectangle: return rectangle;

case _rectangle1: return rectangle1;

case _text: return text;

case _text1: return text1;

case _text2: return text2;

case _text3: return text3;

case _rectangle2: return rectangle2;

case _text4: return text4;

case _text5: return text5;

case _rectangle3: return rectangle3;

case _text6: return text6;

case _text7: return text7;

case _rectangle4: return rectangle4;

case _text8: return text8;

case _text9: return text9;

case _line: return line;

case _line2: return line2;

case _line3: return line3;

case _line4: return line4;

case _line5: return line5;

case _text11: return text11;

case _text12: return text12;

case _rectangle5: return rectangle5;

case _text10: return text10;

case _line1: return line1;

case _rectangle6: return rectangle6;

case _text13: return text13;

case _line6: return line6;

case _rectangle7: return rectangle7;

case _text14: return text14;

case _line7: return line7;

case _rectangle8: return rectangle8;

case _text15: return text15;

case _line8: return line8;

case _rectangle9: return rectangle9;

case _text16: return text16;

case _line9: return line9;

case _text17: return text17;

case _text18: return text18;

case _text19: return text19;

case _text20: return text20;

case _text21: return text21;

case _text22: return text22;

case _text23: return text23;

default: return super.getPersistentShape( _shape );

}

}

@Override

@AnyLogicInternalCodegenAPI

public String getNameOfShape_xjal( Object _shape ) {

try {

if ( _shape == null ) return null;

String _name_xjal;

_name_xjal = checkNameOfShape_xjal( _shape, presentation, "presentation" ); if (_name_xjal != null) return _name_xjal;

_name_xjal = checkNameOfShape_xjal( _shape, icon, "icon" ); if (_name_xjal != null) return _name_xjal;

_name_xjal = checkNameOfShape_xjal( _shape, rectangle, "rectangle" ); if (_name_xjal != null) return _name_xjal;

_name_xjal = checkNameOfShape_xjal( _shape, rectangle1, "rectangle1" ); if (_name_xjal != null) return _name_xjal;

_name_xjal = checkNameOfShape_xjal( _shape, text, "text" ); if (_name_xjal != null) return _name_xjal;

_name_xjal = checkNameOfShape_xjal( _shape, text1, "text1" ); if (_name_xjal != null) return _name_xjal;

_name_xjal = checkNameOfShape_xjal( _shape, text2, "text2" ); if (_name_xjal != null) return _name_xjal;

_name_xjal = checkNameOfShape_xjal( _shape, text3, "text3" ); if (_name_xjal != null) return _name_xjal;

_name_xjal = checkNameOfShape_xjal( _shape, rectangle2, "rectangle2" ); if (_name_xjal != null) return _name_xjal;

_name_xjal = checkNameOfShape_xjal( _shape, text4, "text4" ); if (_name_xjal != null) return _name_xjal;

_name_xjal = checkNameOfShape_xjal( _shape, text5, "text5" ); if (_name_xjal != null) return _name_xjal;

_name_xjal = checkNameOfShape_xjal( _shape, rectangle3, "rectangle3" ); if (_name_xjal != null) return _name_xjal;

_name_xjal = checkNameOfShape_xjal( _shape, text6, "text6" ); if (_name_xjal != null) return _name_xjal;

_name_xjal = checkNameOfShape_xjal( _shape, text7, "text7" ); if (_name_xjal != null) return _name_xjal;

_name_xjal = checkNameOfShape_xjal( _shape, rectangle4, "rectangle4" ); if (_name_xjal != null) return _name_xjal;

_name_xjal = checkNameOfShape_xjal( _shape, text8, "text8" ); if (_name_xjal != null) return _name_xjal;

_name_xjal = checkNameOfShape_xjal( _shape, text9, "text9" ); if (_name_xjal != null) return _name_xjal;

_name_xjal = checkNameOfShape_xjal( _shape, line, "line" ); if (_name_xjal != null) return _name_xjal;

_name_xjal = checkNameOfShape_xjal( _shape, line2, "line2" ); if (_name_xjal != null) return _name_xjal;

_name_xjal = checkNameOfShape_xjal( _shape, line3, "line3" ); if (_name_xjal != null) return _name_xjal;

_name_xjal = checkNameOfShape_xjal( _shape, line4, "line4" ); if (_name_xjal != null) return _name_xjal;

_name_xjal = checkNameOfShape_xjal( _shape, line5, "line5" ); if (_name_xjal != null) return _name_xjal;

_name_xjal = checkNameOfShape_xjal( _shape, text11, "text11" ); if (_name_xjal != null) return _name_xjal;

_name_xjal = checkNameOfShape_xjal( _shape, text12, "text12" ); if (_name_xjal != null) return _name_xjal;

_name_xjal = checkNameOfShape_xjal( _shape, rectangle5, "rectangle5" ); if (_name_xjal != null) return _name_xjal;

_name_xjal = checkNameOfShape_xjal( _shape, text10, "text10" ); if (_name_xjal != null) return _name_xjal;

_name_xjal = checkNameOfShape_xjal( _shape, line1, "line1" ); if (_name_xjal != null) return _name_xjal;

_name_xjal = checkNameOfShape_xjal( _shape, rectangle6, "rectangle6" ); if (_name_xjal != null) return _name_xjal;

_name_xjal = checkNameOfShape_xjal( _shape, text13, "text13" ); if (_name_xjal != null) return _name_xjal;

_name_xjal = checkNameOfShape_xjal( _shape, line6, "line6" ); if (_name_xjal != null) return _name_xjal;

_name_xjal = checkNameOfShape_xjal( _shape, rectangle7, "rectangle7" ); if (_name_xjal != null) return _name_xjal;

_name_xjal = checkNameOfShape_xjal( _shape, text14, "text14" ); if (_name_xjal != null) return _name_xjal;

_name_xjal = checkNameOfShape_xjal( _shape, line7, "line7" ); if (_name_xjal != null) return _name_xjal;

_name_xjal = checkNameOfShape_xjal( _shape, rectangle8, "rectangle8" ); if (_name_xjal != null) return _name_xjal;

_name_xjal = checkNameOfShape_xjal( _shape, text15, "text15" ); if (_name_xjal != null) return _name_xjal;

_name_xjal = checkNameOfShape_xjal( _shape, line8, "line8" ); if (_name_xjal != null) return _name_xjal;

_name_xjal = checkNameOfShape_xjal( _shape, rectangle9, "rectangle9" ); if (_name_xjal != null) return _name_xjal;

_name_xjal = checkNameOfShape_xjal( _shape, text16, "text16" ); if (_name_xjal != null) return _name_xjal;

_name_xjal = checkNameOfShape_xjal( _shape, line9, "line9" ); if (_name_xjal != null) return _name_xjal;

_name_xjal = checkNameOfShape_xjal( _shape, text17, "text17" ); if (_name_xjal != null) return _name_xjal;

_name_xjal = checkNameOfShape_xjal( _shape, text18, "text18" ); if (_name_xjal != null) return _name_xjal;

_name_xjal = checkNameOfShape_xjal( _shape, text19, "text19" ); if (_name_xjal != null) return _name_xjal;

_name_xjal = checkNameOfShape_xjal( _shape, text20, "text20" ); if (_name_xjal != null) return _name_xjal;

_name_xjal = checkNameOfShape_xjal( _shape, text21, "text21" ); if (_name_xjal != null) return _name_xjal;

_name_xjal = checkNameOfShape_xjal( _shape, text22, "text22" ); if (_name_xjal != null) return _name_xjal;

_name_xjal = checkNameOfShape_xjal( _shape, text23, "text23" ); if (_name_xjal != null) return _name_xjal;

} catch (Exception e) {

return null;

}

return super.getNameOfShape_xjal( _shape );

}

@AnyLogicInternalCodegenAPI

private void drawModelElements_Events_xjal(Panel _panel, Graphics2D _g, boolean _publicOnly, boolean _isSuperClass ) {

if (!_publicOnly) {

drawEvent( _panel, _g, 240, 20, 10, 0, "Deterioration", Deterioration );

}

if (!_publicOnly) {

drawEvent( _panel, _g, 220, -20, 10, 0, "Leakage", Leakage );

}

if (!_publicOnly) {

drawEvent( _panel, _g, 400, -90, 10, 0, "Breakage", Breakage );

}

if (!_publicOnly) {

drawEvent( _panel, _g, 710, 70, 10, 0, "BreakageFiveYearEvent", BreakageFiveYearEvent );

}

}

@AnyLogicInternalCodegenAPI

private void drawModelElements_Parameters_xjal(Panel _panel, Graphics2D _g, boolean _publicOnly, boolean _isSuperClass ) {

if (!_publicOnly) {

drawParameter( _panel, _g, -140, 20, 10, 0, "RehabFraction", RehabFraction, 0 );

}

if (!_publicOnly) {

drawParameter( _panel, _g, -150, 310, 10, 0, "TotalLengthPipeMile", TotalLengthPipeMile, 0 );

}

}

@AnyLogicInternalCodegenAPI

private void drawModelElements_PlainVariables_xjal(Panel _panel, Graphics2D _g, boolean _publicOnly, boolean _isSuperClass ) {

if (!_publicOnly) {

drawPlainVariable( _panel, _g, 290, 370, 15, 0, "AnnualNetworkBreakage", AnnualNetworkBreakage, false );

}

if (!_publicOnly) {

drawPlainVariable( _panel, _g, 40, 370, 10, 0, "AnnualWaterLoss_Breakage_MG", AnnualWaterLoss_Breakage_MG, false );

}

if (!_publicOnly) {

drawPlainVariable( _panel, _g, 220, -60, 10, 0, "LeakAmount_g_km_year", LeakAmount_g_km_year, false );

}

if (!_publicOnly) {

drawPlainVariable( _panel, _g, 220, -90, 10, 0, "TotalLeakAmount", TotalLeakAmount, false );

}

if (!_publicOnly) {

drawPlainVariable( _panel, _g, 710, 150, 10, 0, "YearCounter", YearCounter, false );

}

}

@AnyLogicInternalCodegenAPI

private void drawModelElements_CollectionVariables_xjal(Panel _panel, Graphics2D _g, boolean _publicOnly, boolean _isSuperClass ) {

if (!_publicOnly) {

drawCollection( _panel, _g, 70, 60, 10, 0, "ConditionGroups", ConditionGroups );

}

if (!_publicOnly) {

drawCollection( _panel, _g, 70, 20, 10, 0, "ConditionGroupsYears", ConditionGroupsYears );

}

if (!_publicOnly) {

drawCollection( _panel, _g, 220, 60, 10, 0, "CGLambdas", CGLambdas );

}

if (!_publicOnly) {

drawCollection( _panel, _g, 360, 20, 10, 0, "CG_PVC", CG_PVC );

}

if (!_publicOnly) {

drawCollection( _panel, _g, 360, 60, 10, 0, "CG_CI", CG_CI );

}

if (!_publicOnly) {

drawCollection( _panel, _g, 450, 20, 10, 0, "Lambda_Break_PVC", Lambda_Break_PVC );

}

if (!_publicOnly) {

drawCollection( _panel, _g, 450, 60, 10, 0, "Lambda_Break_CI", Lambda_Break_CI );

}

if (!_publicOnly) {

drawCollection( _panel, _g, 610, 20, 10, 0, "Leak_PVC", Leak_PVC );

}

if (!_publicOnly) {

drawCollection( _panel, _g, 610, 60, 10, 0, "Leak_CI", Leak_CI );

}

if (!_publicOnly) {

drawCollection( _panel, _g, 710, 20, 10, 0, "FiveYearBreakSummary", FiveYearBreakSummary );

}

}

@AnyLogicInternalCodegenAPI

private void drawModelElements_AuxVariables_xjal(Panel _panel, Graphics2D _g, boolean _publicOnly, boolean _isSuperClass ) {

if (!_publicOnly) {

drawAuxiliaryVariable( _panel, _g, -150, 400, 15, 0, "LeakageAmount", LeakageAmount, null, 0 );

}

if (!_publicOnly) {

drawAuxiliaryVariable( _panel, _g, -150, 430, 15, 0, "AnnualDemand", AnnualDemand, null, 0 );

}

if (!_publicOnly) {

drawAuxiliaryVariable( _panel, _g, 40, 520, 20, 0, "ABreaks", ABreaks, null, 0 );

}

if (!_publicOnly) {

drawAuxiliaryVariable( _panel, _g, 40, 550, 20, 0, "TotalPVC", TotalPVC, null, 0 );

}

if (!_publicOnly) {

drawAuxiliaryVariable( _panel, _g, 40, 580, 20, 0, "TotalCI", TotalCI, null, 0 );

}

if (!_publicOnly) {

drawAuxiliaryVariable( _panel, _g, 20, 410, 20, 0, "AnnualTotalWaterLoss", AnnualTotalWaterLoss, null, 0 );

}

if (!_publicOnly) {

drawAuxiliaryVariable( _panel, _g, 20, 450, 20, 0, "AnnualEnergyLoss_Mwh", AnnualEnergyLoss_Mwh, null, 0 );

}

}

@AnyLogicInternalCodegenAPI

private static void createLinkArcs_xjal() {

_arc_PD_1468534237245_xjal = new Arc2D.Double(-170.00000000000014, 399.375, 31.249999999999996, 31.249999999999996, 252.78368377685547, -122.98571014404297, Arc2D.OPEN);

_arc_PD_1469045550131_xjal = new Arc2D.Double(-266.3061459469395, 384.7044862732221, 382.5000000000003, 382.5000000000003, 110.37396240234375, -45.68304443359375, Arc2D.OPEN);

_arc_PD_1469045581859_xjal = new Arc2D.Double(5.263932997746387, 366.3819666432382, 44.999999999999986, 44.999999999999986, 31.37639617919922, -103.02214813232422, Arc2D.OPEN);

_arc_PD_1469046331960_xjal = new Arc2D.Double(4.618527782440651E-14, 410.0, 40.0, 40.0, 63.9942512512207, -110.53089141845703, Arc2D.OPEN);

}

@AnyLogicInternalCodegenAPI

protected static Arc2D.Double _arc_PD_1468534237245_xjal;

@AnyLogicInternalCodegenAPI

protected static Arc2D.Double _arc_PD_1469045550131_xjal;

@AnyLogicInternalCodegenAPI

protected static Arc2D.Double _arc_PD_1469045581859_xjal;

@AnyLogicInternalCodegenAPI

protected static Arc2D.Double _arc_PD_1469046331960_xjal;

static {

createLinkArcs_xjal();

}

@AnyLogicInternalCodegenAPI

private void drawModelElements_Links_xjal(Panel _panel, Graphics2D _g, boolean _publicOnly, boolean _isSuperClass ) {

if (!_publicOnly) {

drawLink( _panel, _g, _arc_PD_1468534237245_xjal, null, 1, null, 0.95f, 0 );

}

if (!_publicOnly) {

drawLink( _panel, _g, _arc_PD_1469045550131_xjal, null, 1, null, 0.95f, 0 );

}

if (!_publicOnly) {

drawLink( _panel, _g, _arc_PD_1469045581859_xjal, null, 1, null, 0.95f, 0 );

}

if (!_publicOnly) {

drawLink( _panel, _g, _arc_PD_1469046331960_xjal, null, 1, null, 0.95f, 0 );

}

}

@AnyLogicInternalCodegenAPI

private void drawModelElements_Functions_xjal(Panel _panel, Graphics2D _g, boolean _publicOnly, boolean _isSuperClass ) {

if (!_publicOnly) {

drawFunction( _panel, _g, -140, -30, 10, 0, "printf");

}

if (!_publicOnly) {

drawFunction( _panel, _g, -140, 60, 10, 0, "TotalLengthPipes");

}

if (!_publicOnly) {

drawFunction( _panel, _g, -140, 100, 10, 0, "AverageCondition");

}

if (!_publicOnly) {

drawFunction( _panel, _g, -140, -60, 10, 0, "round");

}

if (!_publicOnly) {

drawFunction( _panel, _g, -140, 240, 10, 0, "RenewCG100");

}

if (!_publicOnly) {

drawFunction( _panel, _g, -150, 360, 10, 0, "TotalLeakagePercentage");

}

if (!_publicOnly) {

drawFunction( _panel, _g, -150, 520, 10, 0, "AnnualBreaks");

}

if (!_publicOnly) {

drawFunction( _panel, _g, -150, 550, 10, 0, "TotalPVCPercentage");

}

if (!_publicOnly) {

drawFunction( _panel, _g, -150, 580, 10, 0, "TotalCIPercentage");

}

if (!_publicOnly) {

drawFunction( _panel, _g, -80, -30, 10, 0, "printf_int");

}

if (!_publicOnly) {

drawFunction( _panel, _g, 340, -20, 10, 0, "material_deterioration");

}

if (!_publicOnly) {

drawFunction( _panel, _g, 490, -90, 10, 0, "CalculateL80PercentageRA");

}

if (!_publicOnly) {

drawFunction( _panel, _g, 490, -60, 10, 0, "CalculateBreakage");

}

if (!_publicOnly) {

drawFunction( _panel, _g, 670, -90, 10, 0, "CalculateL80PercentageRN");

}

if (!_publicOnly) {

drawFunction( _panel, _g, 670, -60, 10, 0, "CalculateAverageCondition");

}

if (!_publicOnly) {

drawFunction( _panel, _g, 710, 110, 10, 0, "AverageFiveYearBreakage");

}

}

@AnyLogicInternalCodegenAPI

private void drawModelElements_AgentLinks_xjal(Panel _panel, Graphics2D _g, boolean _publicOnly, boolean _isSuperClass ) {

if (_publicOnly) { return; }

drawLinkToContainer( _panel, _g, 50, -100, 10, 0, "main", main );

drawLinkToAgent( _panel, _g, 50, -50, 15, 0, "connections", true, connections );

}

@Override

@AnyLogicInternalCodegenAPI

public void drawModelElements( Panel _panel, Graphics2D _g, boolean _publicOnly, boolean _isSuperClass ) {

super.drawModelElements( _panel, _g, _publicOnly, true );

drawModelElements_Events_xjal( _panel, _g, _publicOnly, _isSuperClass );

drawModelElements_Parameters_xjal( _panel, _g, _publicOnly, _isSuperClass );

drawModelElements_PlainVariables_xjal( _panel, _g, _publicOnly, _isSuperClass );

drawModelElements_CollectionVariables_xjal( _panel, _g, _publicOnly, _isSuperClass );

drawModelElements_AuxVariables_xjal( _panel, _g, _publicOnly, _isSuperClass );

drawModelElements_Links_xjal( _panel, _g, _publicOnly, _isSuperClass );

drawModelElements_Functions_xjal( _panel, _g, _publicOnly, _isSuperClass );

drawModelElements_AgentLinks_xjal( _panel, _g, _publicOnly, _isSuperClass );

}

@AnyLogicInternalCodegenAPI

private boolean onClickModelAt_AgentLinks_xjal( Panel _panel, double _x, double _y, int _clickCount, boolean _publicOnly, boolean _isSuperClass ) {

if ( modelElementContains(_x, _y, 50, -100) ) {

if ( _clickCount == 2 ) {

_panel.browseAgent_xjal( 50, -100, this, "main" );

} else {

_panel.addInspect( 50, -100, this, "main" );

}

return true;

}

if ( modelElementContains(_x, _y, 50, -50) ) {

_panel.addInspect_xjal( 50, -50, this, "connections", Panel.INSPECT_CONNECTIONS_xjal );

return true;

}

return false;

}

@AnyLogicInternalCodegenAPI

private boolean onClickModelAt_Parameters_xjal( Panel _panel, double _x, double _y, int _clickCount, boolean _publicOnly, boolean _isSuperClass ) {

if( !_publicOnly && modelElementContains(_x, _y, -140, 20) ) {

_panel.addInspect( -140, 20, this, "RehabFraction" );

return true;

}

if( !_publicOnly && modelElementContains(_x, _y, -150, 310) ) {

_panel.addInspect( -150, 310, this, "TotalLengthPipeMile" );

return true;

}

return false;

}

@AnyLogicInternalCodegenAPI

private boolean onClickModelAt_PlainVariables_xjal( Panel _panel, double _x, double _y, int _clickCount, boolean _publicOnly, boolean _isSuperClass ) {

if( !_publicOnly && modelElementContains(_x, _y, 290, 370) ) {

_panel.addInspect( 290, 370, this, "AnnualNetworkBreakage" );

return true;

}

if( !_publicOnly && modelElementContains(_x, _y, 40, 370) ) {

_panel.addInspect( 40, 370, this, "AnnualWaterLoss_Breakage_MG" );

return true;

}

if( !_publicOnly && modelElementContains(_x, _y, 220, -60) ) {

_panel.addInspect( 220, -60, this, "LeakAmount_g_km_year" );

return true;

}

if( !_publicOnly && modelElementContains(_x, _y, 220, -90) ) {

_panel.addInspect( 220, -90, this, "TotalLeakAmount" );

return true;

}

if( !_publicOnly && modelElementContains(_x, _y, 710, 150) ) {

_panel.addInspect( 710, 150, this, "YearCounter" );

return true;

}

return false;

}

@AnyLogicInternalCodegenAPI

private boolean onClickModelAt_AuxVariables_xjal( Panel _panel, double _x, double _y, int _clickCount, boolean _publicOnly, boolean _isSuperClass ) {

if( !_publicOnly && modelElementContains(_x, _y, -150, 400) ) {

_panel.addInspect_xjal( -150, 400, this, "LeakageAmount", Panel.INSPECT_READ_ONLY_xjal );

return true;

}

if( !_publicOnly && modelElementContains(_x, _y, -150, 430) ) {

_panel.addInspect_xjal( -150, 430, this, "AnnualDemand", Panel.INSPECT_READ_ONLY_xjal );

return true;

}

if( !_publicOnly && modelElementContains(_x, _y, 40, 520) ) {

_panel.addInspect_xjal( 40, 520, this, "ABreaks", Panel.INSPECT_READ_ONLY_xjal );

return true;

}

if( !_publicOnly && modelElementContains(_x, _y, 40, 550) ) {

_panel.addInspect_xjal( 40, 550, this, "TotalPVC", Panel.INSPECT_READ_ONLY_xjal );

return true;

}

if( !_publicOnly && modelElementContains(_x, _y, 40, 580) ) {

_panel.addInspect_xjal( 40, 580, this, "TotalCI", Panel.INSPECT_READ_ONLY_xjal );

return true;

}

if( !_publicOnly && modelElementContains(_x, _y, 20, 410) ) {

_panel.addInspect_xjal( 20, 410, this, "AnnualTotalWaterLoss", Panel.INSPECT_READ_ONLY_xjal );

return true;

}

if( !_publicOnly && modelElementContains(_x, _y, 20, 450) ) {

_panel.addInspect_xjal( 20, 450, this, "AnnualEnergyLoss_Mwh", Panel.INSPECT_READ_ONLY_xjal );

return true;

}

return false;

}

@AnyLogicInternalCodegenAPI

private boolean onClickModelAt_CollectionVariables_xjal( Panel _panel, double _x, double _y, int _clickCount, boolean _publicOnly, boolean _isSuperClass ) {

if( !_publicOnly && modelElementContains(_x, _y, 70, 60) ) {

_panel.addInspect( 70, 60, this, "ConditionGroups" );

return true;

}

if( !_publicOnly && modelElementContains(_x, _y, 70, 20) ) {

_panel.addInspect( 70, 20, this, "ConditionGroupsYears" );

return true;

}

if( !_publicOnly && modelElementContains(_x, _y, 220, 60) ) {

_panel.addInspect( 220, 60, this, "CGLambdas" );

return true;

}

if( !_publicOnly && modelElementContains(_x, _y, 360, 20) ) {

_panel.addInspect( 360, 20, this, "CG_PVC" );

return true;

}

if( !_publicOnly && modelElementContains(_x, _y, 360, 60) ) {

_panel.addInspect( 360, 60, this, "CG_CI" );

return true;

}

if( !_publicOnly && modelElementContains(_x, _y, 450, 20) ) {

_panel.addInspect( 450, 20, this, "Lambda_Break_PVC" );

return true;

}

if( !_publicOnly && modelElementContains(_x, _y, 450, 60) ) {

_panel.addInspect( 450, 60, this, "Lambda_Break_CI" );

return true;

}

if( !_publicOnly && modelElementContains(_x, _y, 610, 20) ) {

_panel.addInspect( 610, 20, this, "Leak_PVC" );

return true;

}

if( !_publicOnly && modelElementContains(_x, _y, 610, 60) ) {

_panel.addInspect( 610, 60, this, "Leak_CI" );

return true;

}

if( !_publicOnly && modelElementContains(_x, _y, 710, 20) ) {

_panel.addInspect( 710, 20, this, "FiveYearBreakSummary" );

return true;

}

return false;

}

@AnyLogicInternalCodegenAPI

private boolean onClickModelAt_Events_xjal( Panel _panel, double _x, double _y, int _clickCount, boolean _publicOnly, boolean _isSuperClass ) {

if( !_publicOnly && modelElementContains(_x, _y, 240, 20) ) {

_panel.addInspect( 240, 20, this, "Deterioration" );

return true;

}

if( !_publicOnly && modelElementContains(_x, _y, 220, -20) ) {

_panel.addInspect( 220, -20, this, "Leakage" );

return true;

}

if( !_publicOnly && modelElementContains(_x, _y, 400, -90) ) {

_panel.addInspect( 400, -90, this, "Breakage" );

return true;

}

if( !_publicOnly && modelElementContains(_x, _y, 710, 70) ) {

_panel.addInspect( 710, 70, this, "BreakageFiveYearEvent" );

return true;

}

return false;

}

@Override

@AnyLogicInternalCodegenAPI

public boolean onClickModelAt( Panel _panel, double _x, double _y, int _clickCount, boolean _publicOnly, boolean _isSuperClass ) {

if ( onClickModelAt_AgentLinks_xjal( _panel, _x, _y, _clickCount, _publicOnly, _isSuperClass ) ) { return true; }

if ( onClickModelAt_Parameters_xjal( _panel, _x, _y, _clickCount, _publicOnly, _isSuperClass ) ) { return true; }

if ( onClickModelAt_PlainVariables_xjal( _panel, _x, _y, _clickCount, _publicOnly, _isSuperClass ) ) { return true; }

if ( onClickModelAt_AuxVariables_xjal( _panel, _x, _y, _clickCount, _publicOnly, _isSuperClass ) ) { return true; }

if ( onClickModelAt_CollectionVariables_xjal( _panel, _x, _y, _clickCount, _publicOnly, _isSuperClass ) ) { return true; }

if ( onClickModelAt_Events_xjal( _panel, _x, _y, _clickCount, _publicOnly, _isSuperClass ) ) { return true; }

return super.onClickModelAt( _panel, _x, _y, _clickCount, _publicOnly, true );

}

/**

* Constructor

*/

public Pipe( Engine engine, Agent owner, AgentList<? extends Pipe> ownerPopulation ) {

super( engine, owner, ownerPopulation );

instantiateBaseStructureThis_xjal();

}

@AnyLogicInternalCodegenAPI

public void onOwnerChanged_xjal() {

super.onOwnerChanged_xjal();

setupReferences_xjal();

}

@AnyLogicInternalCodegenAPI

public void instantiateBaseStructure_xjal() {

super.instantiateBaseStructure_xjal();

instantiateBaseStructureThis_xjal();

}

@AnyLogicInternalCodegenAPI

private void instantiateBaseStructureThis_xjal() {

setupReferences_xjal();

// Registering in Engine continuous part

getEngine().registerAgentWithEquations( this );

}

@AnyLogicInternalCodegenAPI

private void setupReferences_xjal() {

main = get_Main();

}

/**

* Simple constructor. Please add created agent to some population by calling goToPopulation() function

*/

public Pipe() {

}

/**

* Simple constructor. Please add created agent to some population by calling goToPopulation() function

*/

public Pipe( double RehabFraction, double TotalLengthPipeMile ) {

markParametersAreSet();

this.RehabFraction = RehabFraction;

this.TotalLengthPipeMile = TotalLengthPipeMile;

}

@Override

@AnyLogicInternalCodegenAPI

public void doCreate() {

super.doCreate();

// Assigning initial values for plain variables

setupPlainVariables_Pipe_xjal();

// Dynamic initialization of persistent elements

_createPersistentElementsAP0_xjal();

presentation = new ShapeTopLevelPresentationGroup( Pipe.this, true, 0, 0, 0, 0 , text12 );

icon = new ShapeGroup( Pipe.this, true, 0, 0, 0 , rectangle, rectangle1, text, text1, text2, text3, rectangle2, text4, text5, rectangle3, text6, text7, rectangle4, text8, text9, line, line2, line3, line4, line5, text11, rectangle5, text10, line1, rectangle6, text13, line6, rectangle7, text14, line7, rectangle8, text15, line8, rectangle9, text16, line9, text17, text18, text19, text20, text21, text22, text23 );

addAll( ConditionGroups, new Double[]

{main.CG20LengthChoice,

main.CG40LengthChoice,

main.CG60LengthChoice,

main.CG80LengthChoice,

main.CG100LengthChoice}

);

addAll( ConditionGroupsYears, new Integer[]

{20,40,60,80,100}

);

addAll( CGLambdas,

selectValues(Double.class,

"SELECT lambda FROM condition_groups;"

)

);

addAll( CG_PVC, new Double[]

{main.CG20PVCChoice,

main.CG40PVCChoice,

main.CG60PVCChoice,

main.CG80PVCChoice,

main.CG100PVCChoice}

);

addAll( CG_CI, new Double[]

{100-main.CG20PVCChoice,

100-main.CG40PVCChoice,

100-main.CG60PVCChoice,

100-main.CG80PVCChoice,

100-main.CG100PVCChoice}

);

addAll( Lambda_Break_PVC,

selectValues(Double.class,

"SELECT lambda_break_factor_num_mile_year FROM breakandleakfactor WHERE " +

"material = ?;",

0.07

)

);

addAll( Lambda_Break_CI,

selectValues(Double.class,

"SELECT lambda_break_factor_num_mile_year FROM breakandleakfactor WHERE " +

"material = ?;",

0.078

)

);

addAll( Leak_PVC,

selectValues(Double.class,

"SELECT leak_factor FROM breakandleakfactor WHERE " +

"material = ?;",

0.07

)

);

addAll( Leak_CI,

selectValues(Double.class,

"SELECT leak_factor FROM breakandleakfactor WHERE " +

"material = ?;",

0.078

)

);

// Port connectors with non-replicated objects

// Creating replicated embedded objects

setupInitialConditions_xjal( Pipe.class );

}

@AnyLogicInternalCodegenAPI

public void setupExt_xjal(AgentExtension _ext) {

// Agent properties setup

if ( _ext instanceof ExtAgentWithSpatialMetrics && _ext instanceof ExtWithSpaceType ) {

double _value;

_value =

10

;

((ExtAgentWithSpatialMetrics) _ext).setSpeed( _value, MPS );

}

}

@Override

@AnyLogicInternalCodegenAPI

public void doStart() {

super.doStart();

Deterioration.start();

Leakage.start();

Breakage.start();

BreakageFiveYearEvent.start();

_autoCreatedDS_xjal.start();

}

/**

* Assigning initial values for plain variables<br>

* <em>This method isn't designed to be called by user and may be removed in future releases.</em>

*/

@AnyLogicInternalCodegenAPI

public void setupPlainVariables_xjal() {

setupPlainVariables_Pipe_xjal();

}

/**

* Assigning initial values for plain variables<br>

* <em>This method isn't designed to be called by user and may be removed in future releases.</em>

*/

@AnyLogicInternalCodegenAPI

private void setupPlainVariables_Pipe_xjal() {

LeakAmount_g_km_year =

10

;

TotalLeakAmount =

0

;

YearCounter =

0

;

}

// User API -----------------------------------------------------

public Main get_Main() {

{

Agent owner = getOwner();

if ( owner instanceof Main ) return (Main) owner;

}

return null;

}

/**

* Read-only variable. <em>Shouldn't be modified by user.</em>

*/

@AnyLogicCustomSerialization(AnyLogicCustomSerializationMode.REFERENCE)

public transient waterinfrastructure.Main main;

@AnyLogicInternalCodegenAPI

static LinkToAgentAnimationSettings _connections_commonAnimationSettings_xjal = new LinkToAgentAnimationSettingsImpl( false, black, 1.0, LINE_STYLE_SOLID, ARROW_NONE, 0.0 );

public LinkToAgentCollection<Agent, Agent> connections = new LinkToAgentStandardImpl<Agent, Agent>(this, _connections_commonAnimationSettings_xjal);

@Override

public LinkToAgentCollection<? extends Agent, ? extends Agent> getLinkToAgentStandard_xjal() {

return connections;

}

@AnyLogicInternalCodegenAPI

public void drawLinksToAgents(boolean _underAgents_xjal, LinkToAgentAnimator _animator_xjal) {

super.drawLinksToAgents(_underAgents_xjal, _animator_xjal);

if ( _underAgents_xjal ) {

_animator_xjal.drawLink( this, connections, true, true );

}

}

public AgentList<? extends Pipe> getPopulation() {

return (AgentList<? extends Pipe>) super.getPopulation();

}

public List<? extends Pipe> agentsInRange( double distance ) {

return (List<? extends Pipe>) super.agentsInRange( distance );

}

@Override

@AnyLogicInternalCodegenAPI

public boolean isLoggingToDB(EventOriginator _e) {

if ( _e == _autoCreatedDS_xjal ) return false;

return super.isLoggingToDB( _e );

}

@AnyLogicInternalCodegenAPI

public void onDestroy() {

Deterioration.onDestroy();

Leakage.onDestroy();

Breakage.onDestroy();

BreakageFiveYearEvent.onDestroy();

_autoCreatedDS_xjal.onDestroy();

// Unregistering in Engine continuous part

getEngine().unregisterAgentWithEquations( this );

_ds_LeakageAmount.destroyUpdater_xjal();

_ds_AnnualDemand.destroyUpdater_xjal();

_ds_ABreaks.destroyUpdater_xjal();

_ds_TotalPVC.destroyUpdater_xjal();

_ds_TotalCI.destroyUpdater_xjal();

_ds_AnnualTotalWaterLoss.destroyUpdater_xjal();

_ds_AnnualEnergyLoss_Mwh.destroyUpdater_xjal();

super.onDestroy();

}

}

public class User extends Agent

{

// Parameters

public

double BasePopulation;

/**

* Returns default value for parameter <code>BasePopulation</code>.

* <i>This method should not be called by user</i>

*/

@AnyLogicInternalCodegenAPI

public double _BasePopulation_DefaultValue_xjal() {

final User self = this;

return 0.0;

}

public void set_BasePopulation( double BasePopulation ) {

if (BasePopulation == this.BasePopulation) {

return;

}

double _oldValue_xjal = this.BasePopulation;

this.BasePopulation = BasePopulation;

onChange_BasePopulation_xjal( _oldValue_xjal );

onChange();

}

/**

* Calls "On change" action for parameter BasePopulation.<br>

* Note that 'oldValue' in that action will be unavailable if this method is called by user

* (current parameter value will be passed as 'oldValue').<br>

* Please call <code>set_BasePopulation()</code> method instead.

*/

protected void onChange_BasePopulation() {

onChange_BasePopulation_xjal( BasePopulation );

}

@AnyLogicInternalCodegenAPI

protected void onChange_BasePopulation_xjal( double oldValue ) {

}

public

int NumberHouseholds;

/**

* Returns default value for parameter <code>NumberHouseholds</code>.

* <i>This method should not be called by user</i>

*/

@AnyLogicInternalCodegenAPI

public int _NumberHouseholds_DefaultValue_xjal() {

final User self = this;

return 0;

}

public void set_NumberHouseholds( int NumberHouseholds ) {

if (NumberHouseholds == this.NumberHouseholds) {

return;

}

int _oldValue_xjal = this.NumberHouseholds;

this.NumberHouseholds = NumberHouseholds;

onChange_NumberHouseholds_xjal( _oldValue_xjal );

onChange();

}

/**

* Calls "On change" action for parameter NumberHouseholds.<br>

* Note that 'oldValue' in that action will be unavailable if this method is called by user

* (current parameter value will be passed as 'oldValue').<br>

* Please call <code>set_NumberHouseholds()</code> method instead.

*/

protected void onChange_NumberHouseholds() {

onChange_NumberHouseholds_xjal( NumberHouseholds );

}

@AnyLogicInternalCodegenAPI

protected void onChange_NumberHouseholds_xjal( int oldValue ) {

}

public

double pop_rate;

/**

* Returns default value for parameter <code>pop_rate</code>.

* <i>This method should not be called by user</i>

*/

@AnyLogicInternalCodegenAPI

public double _pop_rate_DefaultValue_xjal() {

final User self = this;

return 0.0;

}

public void set_pop_rate( double pop_rate ) {

if (pop_rate == this.pop_rate) {

return;

}

double _oldValue_xjal = this.pop_rate;

this.pop_rate = pop_rate;

onChange_pop_rate_xjal( _oldValue_xjal );

onChange();

}

/**

* Calls "On change" action for parameter pop_rate.<br>

* Note that 'oldValue' in that action will be unavailable if this method is called by user

* (current parameter value will be passed as 'oldValue').<br>

* Please call <code>set_pop_rate()</code> method instead.

*/

protected void onChange_pop_rate() {

onChange_pop_rate_xjal( pop_rate );

}

@AnyLogicInternalCodegenAPI

protected void onChange_pop_rate_xjal( double oldValue ) {

}

public

int t;

/**

* Returns default value for parameter <code>t</code>.

* <i>This method should not be called by user</i>

*/

@AnyLogicInternalCodegenAPI

public int _t_DefaultValue_xjal() {

final User self = this;

return 0;

}

public void set_t( int t ) {

if (t == this.t) {

return;

}

int _oldValue_xjal = this.t;

this.t = t;

onChange_t_xjal( _oldValue_xjal );

onChange();

}

/**

* Calls "On change" action for parameter t.<br>

* Note that 'oldValue' in that action will be unavailable if this method is called by user

* (current parameter value will be passed as 'oldValue').<br>

* Please call <code>set_t()</code> method instead.

*/

protected void onChange_t() {

onChange_t_xjal( t );

}

@AnyLogicInternalCodegenAPI

protected void onChange_t_xjal( int oldValue ) {

}

@Override

public void setParametersToDefaultValues() {

super.setParametersToDefaultValues();

BasePopulation = _BasePopulation_DefaultValue_xjal();

NumberHouseholds = _NumberHouseholds_DefaultValue_xjal();

pop_rate = _pop_rate_DefaultValue_xjal();

t = _t_DefaultValue_xjal();

}

@Override

public boolean setParameter(String _name_xjal, Object _value_xjal, boolean _callOnChange_xjal) {

switch ( _name_xjal ) {

case "BasePopulation":

if ( _callOnChange_xjal ) {

set_BasePopulation( ((Number) _value_xjal).doubleValue() );

} else {

BasePopulation = ((Number) _value_xjal).doubleValue();

}

return true;

case "NumberHouseholds":

if ( _callOnChange_xjal ) {

set_NumberHouseholds( ((Number) _value_xjal).intValue() );

} else {

NumberHouseholds = ((Number) _value_xjal).intValue();

}

return true;

case "pop_rate":

if ( _callOnChange_xjal ) {

set_pop_rate( ((Number) _value_xjal).doubleValue() );

} else {

pop_rate = ((Number) _value_xjal).doubleValue();

}

return true;

case "t":

if ( _callOnChange_xjal ) {

set_t( ((Number) _value_xjal).intValue() );

} else {

t = ((Number) _value_xjal).intValue();

}

return true;

default:

return super.setParameter( _name_xjal, _value_xjal, _callOnChange_xjal );

}

}

@Override

public <T> T getParameter(String _name_xjal) {

Object _result_xjal;

switch ( _name_xjal ) {

case "BasePopulation": _result_xjal = BasePopulation; break;

case "NumberHouseholds": _result_xjal = NumberHouseholds; break;

case "pop_rate": _result_xjal = pop_rate; break;

case "t": _result_xjal = t; break;

default: _result_xjal = super.getParameter( _name_xjal ); break;

}

return (T) _result_xjal;

}

@AnyLogicInternalCodegenAPI

private static String[] _parameterNames_xjal;

@Override

public String[] getParameterNames() {

String[] result = _parameterNames_xjal;

if (result == null) {

List<String> list = new ArrayList<>( Arrays.asList( super.getParameterNames() ) );

list.add( "BasePopulation" );

list.add( "NumberHouseholds" );

list.add( "pop_rate" );

list.add( "t" );

result = list.toArray( new String[ list.size() ] );

_parameterNames_xjal = result;

}

return result;

}

// Plain Variables

/**

* Initial value may be changed later

*/

public

double

DemandChange;

public

int

year;

public

double

Basepopulation;

@AnyLogicInternalCodegenAPI

private static Map<String, IElementDescriptor> elementDesciptors_xjal = null;

@AnyLogicInternalCodegenAPI

@Override

public Map<String, IElementDescriptor> getElementDesciptors() {

if (elementDesciptors_xjal == null) {

elementDesciptors_xjal = createElementDescriptors(super.getElementDesciptors(), User.class);

}

return elementDesciptors_xjal;

}

@AnyLogicCustomProposalPriority(type = AnyLogicCustomProposalPriority.Type.STATIC_ELEMENT)

public static final Scale scale = new Scale( 10.0 );

@Override

public Scale getScale() {

return scale;

}

// Events

public EventTimeout YearlyEvent = new EventTimeout(this);

@Override

@AnyLogicInternalCodegenAPI

public String getNameOf( EventTimeout _e ) {

if( _e == YearlyEvent ) return "YearlyEvent";

return super.getNameOf( _e );

}

@Override

@AnyLogicInternalCodegenAPI

public EventTimeout.Mode getModeOf( EventTimeout _e ) {

if ( _e == YearlyEvent ) return EVENT_TIMEOUT_MODE_CYCLIC;

return super.getModeOf( _e );

}

@Override

@AnyLogicInternalCodegenAPI

public double getFirstOccurrenceTime( EventTimeout _e ) {

double _t;

if ( _e == YearlyEvent ) {

_t =

0

;

_t = toModelTime( _t, YEAR );

return _t;

}

return super.getFirstOccurrenceTime( _e );

}

@Override

@AnyLogicInternalCodegenAPI

public double evaluateTimeoutOf( EventTimeout _e ) {

double _t;

if( _e == YearlyEvent) {

_t =

1

;

_t = toModelTime( _t, YEAR );

return _t;

}

return super.evaluateTimeoutOf( _e );

}

@Override

@AnyLogicInternalCodegenAPI

public void executeActionOf( EventTimeout _e ) {

if ( _e == YearlyEvent ) {

EventTimeout self = _e;

year = year + 1;

main.InitialPriceChoice = main.InitialPriceChoice + (main.InitialPriceChoice*(main.waterRateChoice/100));

System.out.println("year: "+year);

System.out.println("pop: "+population());

System.out.println("AnnualDemand: "+getAnnualDemand());

System.out.println("WaterPrice: "+main.InitialPriceChoice);

System.out.println("reveue: "+main.agency.Revenue);

System.out.println("---------------------------------------");

;

return;

}

super.executeActionOf( _e );

}

// Functions

double

getBasicDemand_gall_day( ) {

double alpha=87.4;

double beta=0.69;

if(NumberHouseholds != 0)

return alpha*Math.pow((population()/NumberHouseholds), beta) * NumberHouseholds;

else

return 0;

}

double

getAnnualDemand( ) {

return (getBasicDemand_gall_day()+DemandChange)*365;

//return 60*24*365*main.DemandChoice;

}

double

population( ) {

double pop = Basepopulation*exp(year*main.pop_rateChoice/100);

return pop;

}

double

WaterPrice( ) {

double WP = main.InitialPriceChoice*Math.pow((1+(main.waterRateChoice/100)), year);

return WP;

}

// View areas

public ViewArea _origin_VA = new ViewArea( this, "[Origin]", 0, 0, 1000.0, 590.0 );

@Override

@AnyLogicInternalCodegenAPI

public int getViewAreas(Map<String, ViewArea> _output) {

if ( _output != null ) {

_output.put( "_origin_VA", this._origin_VA );

}

return 1 + super.getViewAreas( _output );

}

/** Internal constant, shouldn't be accessed by user */

@AnyLogicInternalCodegenAPI

protected static final int _SHAPE_NEXT_ID_xjal = 1;

/**

* Top-level presentation group id

*/

@AnyLogicInternalCodegenAPI

protected static final int _presentation = 0;

@AnyLogicInternalCodegenAPI

public boolean isPublicPresentationDefined() {

return false;

}

@AnyLogicInternalCodegenAPI

public boolean isEmbeddedAgentPresentationVisible( Agent _a ) {

return super.isEmbeddedAgentPresentationVisible( _a );

}

/**

* Top-level icon group id

*/

@AnyLogicInternalCodegenAPI

protected static final int _icon = -1;

protected ShapeTopLevelPresentationGroup presentation;

protected ShapeGroup icon;

@Override

@AnyLogicInternalCodegenAPI

public Object getPersistentShape( int _shape ) {

switch (_shape) {

case _presentation: return presentation;

case _icon: return icon;

default: return super.getPersistentShape( _shape );

}

}

@Override

@AnyLogicInternalCodegenAPI

public String getNameOfShape_xjal( Object _shape ) {

try {

if ( _shape == null ) return null;

String _name_xjal;

_name_xjal = checkNameOfShape_xjal( _shape, presentation, "presentation" ); if (_name_xjal != null) return _name_xjal;

_name_xjal = checkNameOfShape_xjal( _shape, icon, "icon" ); if (_name_xjal != null) return _name_xjal;

} catch (Exception e) {

return null;

}

return super.getNameOfShape_xjal( _shape );

}

@AnyLogicInternalCodegenAPI

private void drawModelElements_Events_xjal(Panel _panel, Graphics2D _g, boolean _publicOnly, boolean _isSuperClass ) {

if (!_publicOnly) {

drawEvent( _panel, _g, 100, 120, 10, 0, "YearlyEvent", YearlyEvent );

}

}

@AnyLogicInternalCodegenAPI

private void drawModelElements_Parameters_xjal(Panel _panel, Graphics2D _g, boolean _publicOnly, boolean _isSuperClass ) {

if (!_publicOnly) {

drawParameter( _panel, _g, 30, 30, 10, 0, "BasePopulation", BasePopulation, 0 );

}

if (!_publicOnly) {

drawParameter( _panel, _g, 30, 90, 10, 0, "NumberHouseholds", NumberHouseholds, 0 );

}

if (!_publicOnly) {

drawParameter( _panel, _g, 30, 60, 10, 0, "pop_rate", pop_rate, 0 );

}

if (!_publicOnly) {

drawParameter( _panel, _g, 30, 120, 15, 5, "t", t, 0 );

}

}

@AnyLogicInternalCodegenAPI

private void drawModelElements_PlainVariables_xjal(Panel _panel, Graphics2D _g, boolean _publicOnly, boolean _isSuperClass ) {

if (!_publicOnly) {

drawPlainVariable( _panel, _g, 30, 300, 10, 0, "DemandChange", DemandChange, false );

}

if (!_publicOnly) {

drawPlainVariable( _panel, _g, 240, 110, 10, 0, "year", year, false );

}

if (!_publicOnly) {

drawPlainVariable( _panel, _g, 240, 60, 10, 0, "Basepopulation", Basepopulation, false );

}

}

@AnyLogicInternalCodegenAPI

private void drawModelElements_Functions_xjal(Panel _panel, Graphics2D _g, boolean _publicOnly, boolean _isSuperClass ) {

if (!_publicOnly) {

drawFunction( _panel, _g, 30, 180, 10, 0, "getBasicDemand_gall_day");

}

if (!_publicOnly) {

drawFunction( _panel, _g, 30, 220, 10, 0, "getAnnualDemand");

}

if (!_publicOnly) {

drawFunction( _panel, _g, 30, 150, 10, 0, "population");

}

if (!_publicOnly) {

drawFunction( _panel, _g, 240, 150, 10, 0, "WaterPrice");

}

}

@AnyLogicInternalCodegenAPI

private void drawModelElements_AgentLinks_xjal(Panel _panel, Graphics2D _g, boolean _publicOnly, boolean _isSuperClass ) {

if (_publicOnly) { return; }

drawLinkToContainer( _panel, _g, 50, -100, 10, 0, "main", main );

drawLinkToAgent( _panel, _g, 50, -50, 15, 0, "connections", true, connections );

}

@Override

@AnyLogicInternalCodegenAPI

public void drawModelElements( Panel _panel, Graphics2D _g, boolean _publicOnly, boolean _isSuperClass ) {

super.drawModelElements( _panel, _g, _publicOnly, true );

drawModelElements_Events_xjal( _panel, _g, _publicOnly, _isSuperClass );

drawModelElements_Parameters_xjal( _panel, _g, _publicOnly, _isSuperClass );

drawModelElements_PlainVariables_xjal( _panel, _g, _publicOnly, _isSuperClass );

drawModelElements_Functions_xjal( _panel, _g, _publicOnly, _isSuperClass );

drawModelElements_AgentLinks_xjal( _panel, _g, _publicOnly, _isSuperClass );

}

@AnyLogicInternalCodegenAPI

private boolean onClickModelAt_AgentLinks_xjal( Panel _panel, double _x, double _y, int _clickCount, boolean _publicOnly, boolean _isSuperClass ) {

if ( modelElementContains(_x, _y, 50, -100) ) {

if ( _clickCount == 2 ) {

_panel.browseAgent_xjal( 50, -100, this, "main" );

} else {

_panel.addInspect( 50, -100, this, "main" );

}

return true;

}

if ( modelElementContains(_x, _y, 50, -50) ) {

_panel.addInspect_xjal( 50, -50, this, "connections", Panel.INSPECT_CONNECTIONS_xjal );

return true;

}

return false;

}

@AnyLogicInternalCodegenAPI

private boolean onClickModelAt_Parameters_xjal( Panel _panel, double _x, double _y, int _clickCount, boolean _publicOnly, boolean _isSuperClass ) {

if( !_publicOnly && modelElementContains(_x, _y, 30, 30) ) {

_panel.addInspect( 30, 30, this, "BasePopulation" );

return true;

}

if( !_publicOnly && modelElementContains(_x, _y, 30, 90) ) {

_panel.addInspect( 30, 90, this, "NumberHouseholds" );

return true;

}

if( !_publicOnly && modelElementContains(_x, _y, 30, 60) ) {

_panel.addInspect( 30, 60, this, "pop_rate" );

return true;

}

if( !_publicOnly && modelElementContains(_x, _y, 30, 120) ) {

_panel.addInspect( 30, 120, this, "t" );

return true;

}

return false;

}

@AnyLogicInternalCodegenAPI

private boolean onClickModelAt_PlainVariables_xjal( Panel _panel, double _x, double _y, int _clickCount, boolean _publicOnly, boolean _isSuperClass ) {

if( !_publicOnly && modelElementContains(_x, _y, 30, 300) ) {

_panel.addInspect( 30, 300, this, "DemandChange" );

return true;

}

if( !_publicOnly && modelElementContains(_x, _y, 240, 110) ) {

_panel.addInspect( 240, 110, this, "year" );

return true;

}

if( !_publicOnly && modelElementContains(_x, _y, 240, 60) ) {

_panel.addInspect( 240, 60, this, "Basepopulation" );

return true;

}

return false;

}

@AnyLogicInternalCodegenAPI

private boolean onClickModelAt_Events_xjal( Panel _panel, double _x, double _y, int _clickCount, boolean _publicOnly, boolean _isSuperClass ) {

if( !_publicOnly && modelElementContains(_x, _y, 100, 120) ) {

_panel.addInspect( 100, 120, this, "YearlyEvent" );

return true;

}

return false;

}

@Override

@AnyLogicInternalCodegenAPI

public boolean onClickModelAt( Panel _panel, double _x, double _y, int _clickCount, boolean _publicOnly, boolean _isSuperClass ) {

if ( onClickModelAt_AgentLinks_xjal( _panel, _x, _y, _clickCount, _publicOnly, _isSuperClass ) ) { return true; }

if ( onClickModelAt_Parameters_xjal( _panel, _x, _y, _clickCount, _publicOnly, _isSuperClass ) ) { return true; }

if ( onClickModelAt_PlainVariables_xjal( _panel, _x, _y, _clickCount, _publicOnly, _isSuperClass ) ) { return true; }

if ( onClickModelAt_Events_xjal( _panel, _x, _y, _clickCount, _publicOnly, _isSuperClass ) ) { return true; }

return super.onClickModelAt( _panel, _x, _y, _clickCount, _publicOnly, true );

}

/**

* Constructor

*/

public User( Engine engine, Agent owner, AgentList<? extends User> ownerPopulation ) {

super( engine, owner, ownerPopulation );

instantiateBaseStructureThis_xjal();

}

@AnyLogicInternalCodegenAPI

public void onOwnerChanged_xjal() {

super.onOwnerChanged_xjal();

setupReferences_xjal();

}

@AnyLogicInternalCodegenAPI

public void instantiateBaseStructure_xjal() {

super.instantiateBaseStructure_xjal();

instantiateBaseStructureThis_xjal();

}

@AnyLogicInternalCodegenAPI

private void instantiateBaseStructureThis_xjal() {

setupReferences_xjal();

}

@AnyLogicInternalCodegenAPI

private void setupReferences_xjal() {

main = get_Main();

}

/**

* Simple constructor. Please add created agent to some population by calling goToPopulation() function

*/

public User() {

}

/**

* Simple constructor. Please add created agent to some population by calling goToPopulation() function

*/

public User( double BasePopulation, int NumberHouseholds, double pop_rate, int t ) {

markParametersAreSet();

this.BasePopulation = BasePopulation;

this.NumberHouseholds = NumberHouseholds;

this.pop_rate = pop_rate;

this.t = t;

}

@Override

@AnyLogicInternalCodegenAPI

public void doCreate() {

super.doCreate();

// Assigning initial values for plain variables

setupPlainVariables_User_xjal();

// Dynamic initialization of persistent elements

presentation = new ShapeTopLevelPresentationGroup( User.this, true, 0, 0, 0, 0 );

icon = new ShapeGroup( User.this, true, 0, 0, 0 );

// Port connectors with non-replicated objects

// Creating replicated embedded objects

setupInitialConditions_xjal( User.class );

}

@AnyLogicInternalCodegenAPI

public void setupExt_xjal(AgentExtension _ext) {

// Agent properties setup

if ( _ext instanceof ExtAgentWithSpatialMetrics && _ext instanceof ExtWithSpaceType ) {

double _value;

_value =

10

;

((ExtAgentWithSpatialMetrics) _ext).setSpeed( _value, MPS );

}

}

@Override

@AnyLogicInternalCodegenAPI

public void doStart() {

super.doStart();

YearlyEvent.start();

}

/**

* Assigning initial values for plain variables<br>

* <em>This method isn't designed to be called by user and may be removed in future releases.</em>

*/

@AnyLogicInternalCodegenAPI

public void setupPlainVariables_xjal() {

setupPlainVariables_User_xjal();

}

/**

* Assigning initial values for plain variables<br>

* <em>This method isn't designed to be called by user and may be removed in future releases.</em>

*/

@AnyLogicInternalCodegenAPI

private void setupPlainVariables_User_xjal() {

DemandChange =

0

;

year =

t

;

Basepopulation =

BasePopulation

;

}

// User API -----------------------------------------------------

public Main get_Main() {

{

Agent owner = getOwner();

if ( owner instanceof Main ) return (Main) owner;

}

return null;

}

/**

* Read-only variable. <em>Shouldn't be modified by user.</em>

*/

@AnyLogicCustomSerialization(AnyLogicCustomSerializationMode.REFERENCE)

public transient waterinfrastructure.Main main;

@AnyLogicInternalCodegenAPI

static LinkToAgentAnimationSettings _connections_commonAnimationSettings_xjal = new LinkToAgentAnimationSettingsImpl( false, black, 1.0, LINE_STYLE_SOLID, ARROW_NONE, 0.0 );

public LinkToAgentCollection<Agent, Agent> connections = new LinkToAgentStandardImpl<Agent, Agent>(this, _connections_commonAnimationSettings_xjal);

@Override

public LinkToAgentCollection<? extends Agent, ? extends Agent> getLinkToAgentStandard_xjal() {

return connections;

}

@AnyLogicInternalCodegenAPI

public void drawLinksToAgents(boolean _underAgents_xjal, LinkToAgentAnimator _animator_xjal) {

super.drawLinksToAgents(_underAgents_xjal, _animator_xjal);

if ( _underAgents_xjal ) {

_animator_xjal.drawLink( this, connections, true, true );

}

}

public AgentList<? extends User> getPopulation() {

return (AgentList<? extends User>) super.getPopulation();

}

public List<? extends User> agentsInRange( double distance ) {

return (List<? extends User>) super.agentsInRange( distance );

}

@AnyLogicInternalCodegenAPI

public void onDestroy() {

YearlyEvent.onDestroy();

super.onDestroy();

}

}

public class Main extends Agent

{

// Parameters

public

int PopulationChoice;

/**

* Returns default value for parameter <code>PopulationChoice</code>.

* <i>This method should not be called by user</i>

*/

@AnyLogicInternalCodegenAPI

public int _PopulationChoice_DefaultValue_xjal() {

final Main self = this;

return 0;

}

public void set_PopulationChoice( int PopulationChoice ) {

if (PopulationChoice == this.PopulationChoice) {

return;

}

int _oldValue_xjal = this.PopulationChoice;

this.PopulationChoice = PopulationChoice;

onChange_PopulationChoice_xjal( _oldValue_xjal );

onChange();

}

/**

* Calls "On change" action for parameter PopulationChoice.<br>

* Note that 'oldValue' in that action will be unavailable if this method is called by user

* (current parameter value will be passed as 'oldValue').<br>

* Please call <code>set_PopulationChoice()</code> method instead.

*/

protected void onChange_PopulationChoice() {

onChange_PopulationChoice_xjal( PopulationChoice );

}

@AnyLogicInternalCodegenAPI

protected void onChange_PopulationChoice_xjal( int oldValue ) {

int index;

{

User self = user;

double _value;

_value = PopulationChoice

;

user.set_BasePopulation( _value );

}

}

public

int NumberHouseholdsChoice;

/**

* Returns default value for parameter <code>NumberHouseholdsChoice</code>.

* <i>This method should not be called by user</i>

*/

@AnyLogicInternalCodegenAPI

public int _NumberHouseholdsChoice_DefaultValue_xjal() {

final Main self = this;

return 0;

}

public void set_NumberHouseholdsChoice( int NumberHouseholdsChoice ) {

if (NumberHouseholdsChoice == this.NumberHouseholdsChoice) {

return;

}

int _oldValue_xjal = this.NumberHouseholdsChoice;

this.NumberHouseholdsChoice = NumberHouseholdsChoice;

onChange_NumberHouseholdsChoice_xjal( _oldValue_xjal );

onChange();

}

/**

* Calls "On change" action for parameter NumberHouseholdsChoice.<br>

* Note that 'oldValue' in that action will be unavailable if this method is called by user

* (current parameter value will be passed as 'oldValue').<br>

* Please call <code>set_NumberHouseholdsChoice()</code> method instead.

*/

protected void onChange_NumberHouseholdsChoice() {

onChange_NumberHouseholdsChoice_xjal( NumberHouseholdsChoice );

}

@AnyLogicInternalCodegenAPI

protected void onChange_NumberHouseholdsChoice_xjal( int oldValue ) {

int index;

{

User self = user;

int _value;

_value = NumberHouseholdsChoice

;

user.set_NumberHouseholds( _value );

}

}

public

double InitialPriceChoice;

/**

* Returns default value for parameter <code>InitialPriceChoice</code>.

* <i>This method should not be called by user</i>

*/

@AnyLogicInternalCodegenAPI

public double _InitialPriceChoice_DefaultValue_xjal() {

final Main self = this;

return 0.0;

}

public void set_InitialPriceChoice( double InitialPriceChoice ) {

if (InitialPriceChoice == this.InitialPriceChoice) {

return;

}

double _oldValue_xjal = this.InitialPriceChoice;

this.InitialPriceChoice = InitialPriceChoice;

onChange_InitialPriceChoice_xjal( _oldValue_xjal );

onChange();

}

/**

* Calls "On change" action for parameter InitialPriceChoice.<br>

* Note that 'oldValue' in that action will be unavailable if this method is called by user

* (current parameter value will be passed as 'oldValue').<br>

* Please call <code>set_InitialPriceChoice()</code> method instead.

*/

protected void onChange_InitialPriceChoice() {

onChange_InitialPriceChoice_xjal( InitialPriceChoice );

}

@AnyLogicInternalCodegenAPI

protected void onChange_InitialPriceChoice_xjal( double oldValue ) {

int index;

{

Agency self = agency;

double _value;

_value = InitialPriceChoice

;

agency.set_InputUserFees( _value );

}

}

public

double TotalPipeLength;

/**

* Returns default value for parameter <code>TotalPipeLength</code>.

* <i>This method should not be called by user</i>

*/

@AnyLogicInternalCodegenAPI

public double _TotalPipeLength_DefaultValue_xjal() {

final Main self = this;

return 0.0;

}

public void set_TotalPipeLength( double TotalPipeLength ) {

if (TotalPipeLength == this.TotalPipeLength) {

return;

}

double _oldValue_xjal = this.TotalPipeLength;

this.TotalPipeLength = TotalPipeLength;

onChange_TotalPipeLength_xjal( _oldValue_xjal );

onChange();

}

/**

* Calls "On change" action for parameter TotalPipeLength.<br>

* Note that 'oldValue' in that action will be unavailable if this method is called by user

* (current parameter value will be passed as 'oldValue').<br>

* Please call <code>set_TotalPipeLength()</code> method instead.

*/

protected void onChange_TotalPipeLength() {

onChange_TotalPipeLength_xjal( TotalPipeLength );

}

@AnyLogicInternalCodegenAPI

protected void onChange_TotalPipeLength_xjal( double oldValue ) {

int index;

{

Pipe self = pipe;

double _value;

_value = TotalPipeLength

;

pipe.set_TotalLengthPipeMile( _value );

}

{

Agency self = agency;

double _value;

_value = TotalPipeLength

;

agency.set_TotalPipeLength( _value );

}

}

public

double CG20LengthChoice;

/**

* Returns default value for parameter <code>CG20LengthChoice</code>.

* <i>This method should not be called by user</i>

*/

@AnyLogicInternalCodegenAPI

public double _CG20LengthChoice_DefaultValue_xjal() {

final Main self = this;

return 0.0;

}

public void set_CG20LengthChoice( double CG20LengthChoice ) {

if (CG20LengthChoice == this.CG20LengthChoice) {

return;

}

double _oldValue_xjal = this.CG20LengthChoice;

this.CG20LengthChoice = CG20LengthChoice;

onChange_CG20LengthChoice_xjal( _oldValue_xjal );

onChange();

}

/**

* Calls "On change" action for parameter CG20LengthChoice.<br>

* Note that 'oldValue' in that action will be unavailable if this method is called by user

* (current parameter value will be passed as 'oldValue').<br>

* Please call <code>set_CG20LengthChoice()</code> method instead.

*/

protected void onChange_CG20LengthChoice() {

onChange_CG20LengthChoice_xjal( CG20LengthChoice );

}

@AnyLogicInternalCodegenAPI

protected void onChange_CG20LengthChoice_xjal( double oldValue ) {

}

public

double CG40LengthChoice;

/**

* Returns default value for parameter <code>CG40LengthChoice</code>.

* <i>This method should not be called by user</i>

*/

@AnyLogicInternalCodegenAPI

public double _CG40LengthChoice_DefaultValue_xjal() {

final Main self = this;

return 0.0;

}

public void set_CG40LengthChoice( double CG40LengthChoice ) {

if (CG40LengthChoice == this.CG40LengthChoice) {

return;

}

double _oldValue_xjal = this.CG40LengthChoice;

this.CG40LengthChoice = CG40LengthChoice;

onChange_CG40LengthChoice_xjal( _oldValue_xjal );

onChange();

}

/**

* Calls "On change" action for parameter CG40LengthChoice.<br>

* Note that 'oldValue' in that action will be unavailable if this method is called by user

* (current parameter value will be passed as 'oldValue').<br>

* Please call <code>set_CG40LengthChoice()</code> method instead.

*/

protected void onChange_CG40LengthChoice() {

onChange_CG40LengthChoice_xjal( CG40LengthChoice );

}

@AnyLogicInternalCodegenAPI

protected void onChange_CG40LengthChoice_xjal( double oldValue ) {

}

public

double CG60LengthChoice;

/**

* Returns default value for parameter <code>CG60LengthChoice</code>.

* <i>This method should not be called by user</i>

*/

@AnyLogicInternalCodegenAPI

public double _CG60LengthChoice_DefaultValue_xjal() {

final Main self = this;

return 0.0;

}

public void set_CG60LengthChoice( double CG60LengthChoice ) {

if (CG60LengthChoice == this.CG60LengthChoice) {

return;

}

double _oldValue_xjal = this.CG60LengthChoice;

this.CG60LengthChoice = CG60LengthChoice;

onChange_CG60LengthChoice_xjal( _oldValue_xjal );

onChange();

}

/**

* Calls "On change" action for parameter CG60LengthChoice.<br>

* Note that 'oldValue' in that action will be unavailable if this method is called by user

* (current parameter value will be passed as 'oldValue').<br>

* Please call <code>set_CG60LengthChoice()</code> method instead.

*/

protected void onChange_CG60LengthChoice() {

onChange_CG60LengthChoice_xjal( CG60LengthChoice );

}

@AnyLogicInternalCodegenAPI

protected void onChange_CG60LengthChoice_xjal( double oldValue ) {

}

public

double CG80LengthChoice;

/**

* Returns default value for parameter <code>CG80LengthChoice</code>.

* <i>This method should not be called by user</i>

*/

@AnyLogicInternalCodegenAPI

public double _CG80LengthChoice_DefaultValue_xjal() {

final Main self = this;

return 0.0;

}

public void set_CG80LengthChoice( double CG80LengthChoice ) {

if (CG80LengthChoice == this.CG80LengthChoice) {

return;

}

double _oldValue_xjal = this.CG80LengthChoice;

this.CG80LengthChoice = CG80LengthChoice;

onChange_CG80LengthChoice_xjal( _oldValue_xjal );

onChange();

}

/**

* Calls "On change" action for parameter CG80LengthChoice.<br>

* Note that 'oldValue' in that action will be unavailable if this method is called by user

* (current parameter value will be passed as 'oldValue').<br>

* Please call <code>set_CG80LengthChoice()</code> method instead.

*/

protected void onChange_CG80LengthChoice() {

onChange_CG80LengthChoice_xjal( CG80LengthChoice );

}

@AnyLogicInternalCodegenAPI

protected void onChange_CG80LengthChoice_xjal( double oldValue ) {

}

public

double CG100LengthChoice;

/**

* Returns default value for parameter <code>CG100LengthChoice</code>.

* <i>This method should not be called by user</i>

*/

@AnyLogicInternalCodegenAPI

public double _CG100LengthChoice_DefaultValue_xjal() {

final Main self = this;

return 0.0;

}

public void set_CG100LengthChoice( double CG100LengthChoice ) {

if (CG100LengthChoice == this.CG100LengthChoice) {

return;

}

double _oldValue_xjal = this.CG100LengthChoice;

this.CG100LengthChoice = CG100LengthChoice;

onChange_CG100LengthChoice_xjal( _oldValue_xjal );

onChange();

}

/**

* Calls "On change" action for parameter CG100LengthChoice.<br>

* Note that 'oldValue' in that action will be unavailable if this method is called by user

* (current parameter value will be passed as 'oldValue').<br>

* Please call <code>set_CG100LengthChoice()</code> method instead.

*/

protected void onChange_CG100LengthChoice() {

onChange_CG100LengthChoice_xjal( CG100LengthChoice );

}

@AnyLogicInternalCodegenAPI

protected void onChange_CG100LengthChoice_xjal( double oldValue ) {

}

public

double CG20PVCChoice;

/**

* Returns default value for parameter <code>CG20PVCChoice</code>.

* <i>This method should not be called by user</i>

*/

@AnyLogicInternalCodegenAPI

public double _CG20PVCChoice_DefaultValue_xjal() {

final Main self = this;

return 0.0;

}

public void set_CG20PVCChoice( double CG20PVCChoice ) {

if (CG20PVCChoice == this.CG20PVCChoice) {

return;

}

double _oldValue_xjal = this.CG20PVCChoice;

this.CG20PVCChoice = CG20PVCChoice;

onChange_CG20PVCChoice_xjal( _oldValue_xjal );

onChange();

}

/**

* Calls "On change" action for parameter CG20PVCChoice.<br>

* Note that 'oldValue' in that action will be unavailable if this method is called by user

* (current parameter value will be passed as 'oldValue').<br>

* Please call <code>set_CG20PVCChoice()</code> method instead.

*/

protected void onChange_CG20PVCChoice() {

onChange_CG20PVCChoice_xjal( CG20PVCChoice );

}

@AnyLogicInternalCodegenAPI

protected void onChange_CG20PVCChoice_xjal( double oldValue ) {

}

public

double CG40PVCChoice;

/**

* Returns default value for parameter <code>CG40PVCChoice</code>.

* <i>This method should not be called by user</i>

*/

@AnyLogicInternalCodegenAPI

public double _CG40PVCChoice_DefaultValue_xjal() {

final Main self = this;

return 0.0;

}

public void set_CG40PVCChoice( double CG40PVCChoice ) {

if (CG40PVCChoice == this.CG40PVCChoice) {

return;

}

double _oldValue_xjal = this.CG40PVCChoice;

this.CG40PVCChoice = CG40PVCChoice;

onChange_CG40PVCChoice_xjal( _oldValue_xjal );

onChange();

}

/**

* Calls "On change" action for parameter CG40PVCChoice.<br>

* Note that 'oldValue' in that action will be unavailable if this method is called by user

* (current parameter value will be passed as 'oldValue').<br>

* Please call <code>set_CG40PVCChoice()</code> method instead.

*/

protected void onChange_CG40PVCChoice() {

onChange_CG40PVCChoice_xjal( CG40PVCChoice );

}

@AnyLogicInternalCodegenAPI

protected void onChange_CG40PVCChoice_xjal( double oldValue ) {

}

public

double CG60PVCChoice;

/**

* Returns default value for parameter <code>CG60PVCChoice</code>.

* <i>This method should not be called by user</i>

*/

@AnyLogicInternalCodegenAPI

public double _CG60PVCChoice_DefaultValue_xjal() {

final Main self = this;

return 0.0;

}

public void set_CG60PVCChoice( double CG60PVCChoice ) {

if (CG60PVCChoice == this.CG60PVCChoice) {

return;

}

double _oldValue_xjal = this.CG60PVCChoice;

this.CG60PVCChoice = CG60PVCChoice;

onChange_CG60PVCChoice_xjal( _oldValue_xjal );

onChange();

}

/**

* Calls "On change" action for parameter CG60PVCChoice.<br>

* Note that 'oldValue' in that action will be unavailable if this method is called by user

* (current parameter value will be passed as 'oldValue').<br>

* Please call <code>set_CG60PVCChoice()</code> method instead.

*/

protected void onChange_CG60PVCChoice() {

onChange_CG60PVCChoice_xjal( CG60PVCChoice );

}

@AnyLogicInternalCodegenAPI

protected void onChange_CG60PVCChoice_xjal( double oldValue ) {

}

public

double CG80PVCChoice;

/**

* Returns default value for parameter <code>CG80PVCChoice</code>.

* <i>This method should not be called by user</i>

*/

@AnyLogicInternalCodegenAPI

public double _CG80PVCChoice_DefaultValue_xjal() {

final Main self = this;

return 0.0;

}

public void set_CG80PVCChoice( double CG80PVCChoice ) {

if (CG80PVCChoice == this.CG80PVCChoice) {

return;

}

double _oldValue_xjal = this.CG80PVCChoice;

this.CG80PVCChoice = CG80PVCChoice;

onChange_CG80PVCChoice_xjal( _oldValue_xjal );

onChange();

}

/**

* Calls "On change" action for parameter CG80PVCChoice.<br>

* Note that 'oldValue' in that action will be unavailable if this method is called by user

* (current parameter value will be passed as 'oldValue').<br>

* Please call <code>set_CG80PVCChoice()</code> method instead.

*/

protected void onChange_CG80PVCChoice() {

onChange_CG80PVCChoice_xjal( CG80PVCChoice );

}

@AnyLogicInternalCodegenAPI

protected void onChange_CG80PVCChoice_xjal( double oldValue ) {

}

public

double CG100PVCChoice;

/**

* Returns default value for parameter <code>CG100PVCChoice</code>.

* <i>This method should not be called by user</i>

*/

@AnyLogicInternalCodegenAPI

public double _CG100PVCChoice_DefaultValue_xjal() {

final Main self = this;

return 0.0;

}

public void set_CG100PVCChoice( double CG100PVCChoice ) {

if (CG100PVCChoice == this.CG100PVCChoice) {

return;

}

double _oldValue_xjal = this.CG100PVCChoice;

this.CG100PVCChoice = CG100PVCChoice;

onChange_CG100PVCChoice_xjal( _oldValue_xjal );

onChange();

}

/**

* Calls "On change" action for parameter CG100PVCChoice.<br>

* Note that 'oldValue' in that action will be unavailable if this method is called by user

* (current parameter value will be passed as 'oldValue').<br>

* Please call <code>set_CG100PVCChoice()</code> method instead.

*/

protected void onChange_CG100PVCChoice() {

onChange_CG100PVCChoice_xjal( CG100PVCChoice );

}

@AnyLogicInternalCodegenAPI

protected void onChange_CG100PVCChoice_xjal( double oldValue ) {

}

public

int RiskAttitudeChoice;

/**

* Returns default value for parameter <code>RiskAttitudeChoice</code>.

* <i>This method should not be called by user</i>

*/

@AnyLogicInternalCodegenAPI

public int _RiskAttitudeChoice_DefaultValue_xjal() {

final Main self = this;

return 0;

}

public void set_RiskAttitudeChoice( int RiskAttitudeChoice ) {

if (RiskAttitudeChoice == this.RiskAttitudeChoice) {

return;

}

int _oldValue_xjal = this.RiskAttitudeChoice;

this.RiskAttitudeChoice = RiskAttitudeChoice;

onChange_RiskAttitudeChoice_xjal( _oldValue_xjal );

onChange();

}

/**

* Calls "On change" action for parameter RiskAttitudeChoice.<br>

* Note that 'oldValue' in that action will be unavailable if this method is called by user

* (current parameter value will be passed as 'oldValue').<br>

* Please call <code>set_RiskAttitudeChoice()</code> method instead.

*/

protected void onChange_RiskAttitudeChoice() {

onChange_RiskAttitudeChoice_xjal( RiskAttitudeChoice );

}

@AnyLogicInternalCodegenAPI

protected void onChange_RiskAttitudeChoice_xjal( int oldValue ) {

}

public

double CapitalImprovementChoice;

/**

* Returns default value for parameter <code>CapitalImprovementChoice</code>.

* <i>This method should not be called by user</i>

*/

@AnyLogicInternalCodegenAPI

public double _CapitalImprovementChoice_DefaultValue_xjal() {

final Main self = this;

return 0.0;

}

public void set_CapitalImprovementChoice( double CapitalImprovementChoice ) {

if (CapitalImprovementChoice == this.CapitalImprovementChoice) {

return;

}

double _oldValue_xjal = this.CapitalImprovementChoice;

this.CapitalImprovementChoice = CapitalImprovementChoice;

onChange_CapitalImprovementChoice_xjal( _oldValue_xjal );

onChange();

}

/**

* Calls "On change" action for parameter CapitalImprovementChoice.<br>

* Note that 'oldValue' in that action will be unavailable if this method is called by user

* (current parameter value will be passed as 'oldValue').<br>

* Please call <code>set_CapitalImprovementChoice()</code> method instead.

*/

protected void onChange_CapitalImprovementChoice() {

onChange_CapitalImprovementChoice_xjal( CapitalImprovementChoice );

}

@AnyLogicInternalCodegenAPI

protected void onChange_CapitalImprovementChoice_xjal( double oldValue ) {

int index;

{

Agency self = agency;

double _value;

_value = CapitalImprovementChoice

;

agency.set_CapitalImprovement( _value );

}

}

public

double DesiredAverageConditionChoice;

/**

* Returns default value for parameter <code>DesiredAverageConditionChoice</code>.

* <i>This method should not be called by user</i>

*/

@AnyLogicInternalCodegenAPI

public double _DesiredAverageConditionChoice_DefaultValue_xjal() {

final Main self = this;

return 0.0;

}

public void set_DesiredAverageConditionChoice( double DesiredAverageConditionChoice ) {

if (DesiredAverageConditionChoice == this.DesiredAverageConditionChoice) {

return;

}

double _oldValue_xjal = this.DesiredAverageConditionChoice;

this.DesiredAverageConditionChoice = DesiredAverageConditionChoice;

onChange_DesiredAverageConditionChoice_xjal( _oldValue_xjal );

onChange();

}

/**

* Calls "On change" action for parameter DesiredAverageConditionChoice.<br>

* Note that 'oldValue' in that action will be unavailable if this method is called by user

* (current parameter value will be passed as 'oldValue').<br>

* Please call <code>set_DesiredAverageConditionChoice()</code> method instead.

*/

protected void onChange_DesiredAverageConditionChoice() {

onChange_DesiredAverageConditionChoice_xjal( DesiredAverageConditionChoice );

}

@AnyLogicInternalCodegenAPI

protected void onChange_DesiredAverageConditionChoice_xjal( double oldValue ) {

}

public

int BreakToleranceChoice;

/**

* Returns default value for parameter <code>BreakToleranceChoice</code>.

* <i>This method should not be called by user</i>

*/

@AnyLogicInternalCodegenAPI

public int _BreakToleranceChoice_DefaultValue_xjal() {

final Main self = this;

return 0;

}

public void set_BreakToleranceChoice( int BreakToleranceChoice ) {

if (BreakToleranceChoice == this.BreakToleranceChoice) {

return;

}

int _oldValue_xjal = this.BreakToleranceChoice;

this.BreakToleranceChoice = BreakToleranceChoice;

onChange_BreakToleranceChoice_xjal( _oldValue_xjal );

onChange();

}

/**

* Calls "On change" action for parameter BreakToleranceChoice.<br>

* Note that 'oldValue' in that action will be unavailable if this method is called by user

* (current parameter value will be passed as 'oldValue').<br>

* Please call <code>set_BreakToleranceChoice()</code> method instead.

*/

protected void onChange_BreakToleranceChoice() {

onChange_BreakToleranceChoice_xjal( BreakToleranceChoice );

}

@AnyLogicInternalCodegenAPI

protected void onChange_BreakToleranceChoice_xjal( int oldValue ) {

}

public

double AverageFlowRate;

/**

* Returns default value for parameter <code>AverageFlowRate</code>.

* <i>This method should not be called by user</i>

*/

@AnyLogicInternalCodegenAPI

public double _AverageFlowRate_DefaultValue_xjal() {

final Main self = this;

return 0.0;

}

public void set_AverageFlowRate( double AverageFlowRate ) {

if (AverageFlowRate == this.AverageFlowRate) {

return;

}

double _oldValue_xjal = this.AverageFlowRate;

this.AverageFlowRate = AverageFlowRate;

onChange_AverageFlowRate_xjal( _oldValue_xjal );

onChange();

}

/**

* Calls "On change" action for parameter AverageFlowRate.<br>

* Note that 'oldValue' in that action will be unavailable if this method is called by user

* (current parameter value will be passed as 'oldValue').<br>

* Please call <code>set_AverageFlowRate()</code> method instead.

*/

protected void onChange_AverageFlowRate() {

onChange_AverageFlowRate_xjal( AverageFlowRate );

}

@AnyLogicInternalCodegenAPI

protected void onChange_AverageFlowRate_xjal( double oldValue ) {

}

public

double AveragePressure;

/**

* Returns default value for parameter <code>AveragePressure</code>.

* <i>This method should not be called by user</i>

*/

@AnyLogicInternalCodegenAPI

public double _AveragePressure_DefaultValue_xjal() {

final Main self = this;

return 0.0;

}

public void set_AveragePressure( double AveragePressure ) {

if (AveragePressure == this.AveragePressure) {

return;

}

double _oldValue_xjal = this.AveragePressure;

this.AveragePressure = AveragePressure;

onChange_AveragePressure_xjal( _oldValue_xjal );

onChange();

}

/**

* Calls "On change" action for parameter AveragePressure.<br>

* Note that 'oldValue' in that action will be unavailable if this method is called by user

* (current parameter value will be passed as 'oldValue').<br>

* Please call <code>set_AveragePressure()</code> method instead.

*/

protected void onChange_AveragePressure() {

onChange_AveragePressure_xjal( AveragePressure );

}

@AnyLogicInternalCodegenAPI

protected void onChange_AveragePressure_xjal( double oldValue ) {

}

public

double AverageFailureDuration;

/**

* Returns default value for parameter <code>AverageFailureDuration</code>.

* <i>This method should not be called by user</i>

*/

@AnyLogicInternalCodegenAPI

public double _AverageFailureDuration_DefaultValue_xjal() {

final Main self = this;

return 0.0;

}

public void set_AverageFailureDuration( double AverageFailureDuration ) {

if (AverageFailureDuration == this.AverageFailureDuration) {

return;

}

double _oldValue_xjal = this.AverageFailureDuration;

this.AverageFailureDuration = AverageFailureDuration;

onChange_AverageFailureDuration_xjal( _oldValue_xjal );

onChange();

}

/**

* Calls "On change" action for parameter AverageFailureDuration.<br>

* Note that 'oldValue' in that action will be unavailable if this method is called by user

* (current parameter value will be passed as 'oldValue').<br>

* Please call <code>set_AverageFailureDuration()</code> method instead.

*/

protected void onChange_AverageFailureDuration() {

onChange_AverageFailureDuration_xjal( AverageFailureDuration );

}

@AnyLogicInternalCodegenAPI

protected void onChange_AverageFailureDuration_xjal( double oldValue ) {

}

public

double EnergyIntensityWater;

/**

* Returns default value for parameter <code>EnergyIntensityWater</code>.

* <i>This method should not be called by user</i>

*/

@AnyLogicInternalCodegenAPI

public double _EnergyIntensityWater_DefaultValue_xjal() {

final Main self = this;

return 0.0;

}

public void set_EnergyIntensityWater( double EnergyIntensityWater ) {

if (EnergyIntensityWater == this.EnergyIntensityWater) {

return;

}

double _oldValue_xjal = this.EnergyIntensityWater;

this.EnergyIntensityWater = EnergyIntensityWater;

onChange_EnergyIntensityWater_xjal( _oldValue_xjal );

onChange();

}

/**

* Calls "On change" action for parameter EnergyIntensityWater.<br>

* Note that 'oldValue' in that action will be unavailable if this method is called by user

* (current parameter value will be passed as 'oldValue').<br>

* Please call <code>set_EnergyIntensityWater()</code> method instead.

*/

protected void onChange_EnergyIntensityWater() {

onChange_EnergyIntensityWater_xjal( EnergyIntensityWater );

}

@AnyLogicInternalCodegenAPI

protected void onChange_EnergyIntensityWater_xjal( double oldValue ) {

}

public

double DemandChoice;

/**

* Returns default value for parameter <code>DemandChoice</code>.

* <i>This method should not be called by user</i>

*/

@AnyLogicInternalCodegenAPI

public double _DemandChoice_DefaultValue_xjal() {

final Main self = this;

return 0.0;

}

public void set_DemandChoice( double DemandChoice ) {

if (DemandChoice == this.DemandChoice) {

return;

}

double _oldValue_xjal = this.DemandChoice;

this.DemandChoice = DemandChoice;

onChange_DemandChoice_xjal( _oldValue_xjal );

onChange();

}

/**

* Calls "On change" action for parameter DemandChoice.<br>

* Note that 'oldValue' in that action will be unavailable if this method is called by user

* (current parameter value will be passed as 'oldValue').<br>

* Please call <code>set_DemandChoice()</code> method instead.

*/

protected void onChange_DemandChoice() {

onChange_DemandChoice_xjal( DemandChoice );

}

@AnyLogicInternalCodegenAPI

protected void onChange_DemandChoice_xjal( double oldValue ) {

}

public

double pop_rateChoice;

/**

* Returns default value for parameter <code>pop_rateChoice</code>.

* <i>This method should not be called by user</i>

*/

@AnyLogicInternalCodegenAPI

public double _pop_rateChoice_DefaultValue_xjal() {

final Main self = this;

return 0.0;

}

public void set_pop_rateChoice( double pop_rateChoice ) {

if (pop_rateChoice == this.pop_rateChoice) {

return;

}

double _oldValue_xjal = this.pop_rateChoice;

this.pop_rateChoice = pop_rateChoice;

onChange_pop_rateChoice_xjal( _oldValue_xjal );

onChange();

}

/**

* Calls "On change" action for parameter pop_rateChoice.<br>

* Note that 'oldValue' in that action will be unavailable if this method is called by user

* (current parameter value will be passed as 'oldValue').<br>

* Please call <code>set_pop_rateChoice()</code> method instead.

*/

protected void onChange_pop_rateChoice() {

onChange_pop_rateChoice_xjal( pop_rateChoice );

}

@AnyLogicInternalCodegenAPI

protected void onChange_pop_rateChoice_xjal( double oldValue ) {

}

public

double waterRateChoice;

/**

* Returns default value for parameter <code>waterRateChoice</code>.

* <i>This method should not be called by user</i>

*/

@AnyLogicInternalCodegenAPI

public double _waterRateChoice_DefaultValue_xjal() {

final Main self = this;

return 0.0;

}

public void set_waterRateChoice( double waterRateChoice ) {

if (waterRateChoice == this.waterRateChoice) {

return;

}

double _oldValue_xjal = this.waterRateChoice;

this.waterRateChoice = waterRateChoice;

onChange_waterRateChoice_xjal( _oldValue_xjal );

onChange();

}

/**

* Calls "On change" action for parameter waterRateChoice.<br>

* Note that 'oldValue' in that action will be unavailable if this method is called by user

* (current parameter value will be passed as 'oldValue').<br>

* Please call <code>set_waterRateChoice()</code> method instead.

*/

protected void onChange_waterRateChoice() {

onChange_waterRateChoice_xjal( waterRateChoice );

}

@AnyLogicInternalCodegenAPI

protected void onChange_waterRateChoice_xjal( double oldValue ) {

}

@Override

public void setParametersToDefaultValues() {

super.setParametersToDefaultValues();

PopulationChoice = _PopulationChoice_DefaultValue_xjal();

NumberHouseholdsChoice = _NumberHouseholdsChoice_DefaultValue_xjal();

InitialPriceChoice = _InitialPriceChoice_DefaultValue_xjal();

TotalPipeLength = _TotalPipeLength_DefaultValue_xjal();

CG20LengthChoice = _CG20LengthChoice_DefaultValue_xjal();

CG40LengthChoice = _CG40LengthChoice_DefaultValue_xjal();

CG60LengthChoice = _CG60LengthChoice_DefaultValue_xjal();

CG80LengthChoice = _CG80LengthChoice_DefaultValue_xjal();

CG100LengthChoice = _CG100LengthChoice_DefaultValue_xjal();

CG20PVCChoice = _CG20PVCChoice_DefaultValue_xjal();

CG40PVCChoice = _CG40PVCChoice_DefaultValue_xjal();

CG60PVCChoice = _CG60PVCChoice_DefaultValue_xjal();

CG80PVCChoice = _CG80PVCChoice_DefaultValue_xjal();

CG100PVCChoice = _CG100PVCChoice_DefaultValue_xjal();

RiskAttitudeChoice = _RiskAttitudeChoice_DefaultValue_xjal();

CapitalImprovementChoice = _CapitalImprovementChoice_DefaultValue_xjal();

DesiredAverageConditionChoice = _DesiredAverageConditionChoice_DefaultValue_xjal();

BreakToleranceChoice = _BreakToleranceChoice_DefaultValue_xjal();

AverageFlowRate = _AverageFlowRate_DefaultValue_xjal();

AveragePressure = _AveragePressure_DefaultValue_xjal();

AverageFailureDuration = _AverageFailureDuration_DefaultValue_xjal();

EnergyIntensityWater = _EnergyIntensityWater_DefaultValue_xjal();

DemandChoice = _DemandChoice_DefaultValue_xjal();

pop_rateChoice = _pop_rateChoice_DefaultValue_xjal();

waterRateChoice = _waterRateChoice_DefaultValue_xjal();

}

@Override

public boolean setParameter(String _name_xjal, Object _value_xjal, boolean _callOnChange_xjal) {

switch ( _name_xjal ) {

case "PopulationChoice":

if ( _callOnChange_xjal ) {

set_PopulationChoice( ((Number) _value_xjal).intValue() );

} else {

PopulationChoice = ((Number) _value_xjal).intValue();

}

return true;

case "NumberHouseholdsChoice":

if ( _callOnChange_xjal ) {

set_NumberHouseholdsChoice( ((Number) _value_xjal).intValue() );

} else {

NumberHouseholdsChoice = ((Number) _value_xjal).intValue();

}

return true;

case "InitialPriceChoice":

if ( _callOnChange_xjal ) {

set_InitialPriceChoice( ((Number) _value_xjal).doubleValue() );

} else {

InitialPriceChoice = ((Number) _value_xjal).doubleValue();

}

return true;

case "TotalPipeLength":

if ( _callOnChange_xjal ) {

set_TotalPipeLength( ((Number) _value_xjal).doubleValue() );

} else {

TotalPipeLength = ((Number) _value_xjal).doubleValue();

}

return true;

case "CG20LengthChoice":

if ( _callOnChange_xjal ) {

set_CG20LengthChoice( ((Number) _value_xjal).doubleValue() );

} else {

CG20LengthChoice = ((Number) _value_xjal).doubleValue();

}

return true;

case "CG40LengthChoice":

if ( _callOnChange_xjal ) {

set_CG40LengthChoice( ((Number) _value_xjal).doubleValue() );

} else {

CG40LengthChoice = ((Number) _value_xjal).doubleValue();

}

return true;

case "CG60LengthChoice":

if ( _callOnChange_xjal ) {

set_CG60LengthChoice( ((Number) _value_xjal).doubleValue() );

} else {

CG60LengthChoice = ((Number) _value_xjal).doubleValue();

}

return true;

case "CG80LengthChoice":

if ( _callOnChange_xjal ) {

set_CG80LengthChoice( ((Number) _value_xjal).doubleValue() );

} else {

CG80LengthChoice = ((Number) _value_xjal).doubleValue();

}

return true;

case "CG100LengthChoice":

if ( _callOnChange_xjal ) {

set_CG100LengthChoice( ((Number) _value_xjal).doubleValue() );

} else {

CG100LengthChoice = ((Number) _value_xjal).doubleValue();

}

return true;

case "CG20PVCChoice":

if ( _callOnChange_xjal ) {

set_CG20PVCChoice( ((Number) _value_xjal).doubleValue() );

} else {

CG20PVCChoice = ((Number) _value_xjal).doubleValue();

}

return true;

case "CG40PVCChoice":

if ( _callOnChange_xjal ) {

set_CG40PVCChoice( ((Number) _value_xjal).doubleValue() );

} else {

CG40PVCChoice = ((Number) _value_xjal).doubleValue();

}

return true;

case "CG60PVCChoice":

if ( _callOnChange_xjal ) {

set_CG60PVCChoice( ((Number) _value_xjal).doubleValue() );

} else {

CG60PVCChoice = ((Number) _value_xjal).doubleValue();

}

return true;

case "CG80PVCChoice":

if ( _callOnChange_xjal ) {

set_CG80PVCChoice( ((Number) _value_xjal).doubleValue() );

} else {

CG80PVCChoice = ((Number) _value_xjal).doubleValue();

}

return true;

case "CG100PVCChoice":

if ( _callOnChange_xjal ) {

set_CG100PVCChoice( ((Number) _value_xjal).doubleValue() );

} else {

CG100PVCChoice = ((Number) _value_xjal).doubleValue();

}

return true;

case "RiskAttitudeChoice":

if ( _callOnChange_xjal ) {

set_RiskAttitudeChoice( ((Number) _value_xjal).intValue() );

} else {

RiskAttitudeChoice = ((Number) _value_xjal).intValue();

}

return true;

case "CapitalImprovementChoice":

if ( _callOnChange_xjal ) {

set_CapitalImprovementChoice( ((Number) _value_xjal).doubleValue() );

} else {

CapitalImprovementChoice = ((Number) _value_xjal).doubleValue();

}

return true;

case "DesiredAverageConditionChoice":

if ( _callOnChange_xjal ) {

set_DesiredAverageConditionChoice( ((Number) _value_xjal).doubleValue() );

} else {

DesiredAverageConditionChoice = ((Number) _value_xjal).doubleValue();

}

return true;

case "BreakToleranceChoice":

if ( _callOnChange_xjal ) {

set_BreakToleranceChoice( ((Number) _value_xjal).intValue() );

} else {

BreakToleranceChoice = ((Number) _value_xjal).intValue();

}

return true;

case "AverageFlowRate":

if ( _callOnChange_xjal ) {

set_AverageFlowRate( ((Number) _value_xjal).doubleValue() );

} else {

AverageFlowRate = ((Number) _value_xjal).doubleValue();

}

return true;

case "AveragePressure":

if ( _callOnChange_xjal ) {

set_AveragePressure( ((Number) _value_xjal).doubleValue() );

} else {

AveragePressure = ((Number) _value_xjal).doubleValue();

}

return true;

case "AverageFailureDuration":

if ( _callOnChange_xjal ) {

set_AverageFailureDuration( ((Number) _value_xjal).doubleValue() );

} else {

AverageFailureDuration = ((Number) _value_xjal).doubleValue();

}

return true;

case "EnergyIntensityWater":

if ( _callOnChange_xjal ) {

set_EnergyIntensityWater( ((Number) _value_xjal).doubleValue() );

} else {

EnergyIntensityWater = ((Number) _value_xjal).doubleValue();

}

return true;

case "DemandChoice":

if ( _callOnChange_xjal ) {

set_DemandChoice( ((Number) _value_xjal).doubleValue() );

} else {

DemandChoice = ((Number) _value_xjal).doubleValue();

}

return true;

case "pop_rateChoice":

if ( _callOnChange_xjal ) {

set_pop_rateChoice( ((Number) _value_xjal).doubleValue() );

} else {

pop_rateChoice = ((Number) _value_xjal).doubleValue();

}

return true;

case "waterRateChoice":

if ( _callOnChange_xjal ) {

set_waterRateChoice( ((Number) _value_xjal).doubleValue() );

} else {

waterRateChoice = ((Number) _value_xjal).doubleValue();

}

return true;

default:

return super.setParameter( _name_xjal, _value_xjal, _callOnChange_xjal );

}

}

@Override

public <T> T getParameter(String _name_xjal) {

Object _result_xjal;

switch ( _name_xjal ) {

case "PopulationChoice": _result_xjal = PopulationChoice; break;

case "NumberHouseholdsChoice": _result_xjal = NumberHouseholdsChoice; break;

case "InitialPriceChoice": _result_xjal = InitialPriceChoice; break;

case "TotalPipeLength": _result_xjal = TotalPipeLength; break;

case "CG20LengthChoice": _result_xjal = CG20LengthChoice; break;

case "CG40LengthChoice": _result_xjal = CG40LengthChoice; break;

case "CG60LengthChoice": _result_xjal = CG60LengthChoice; break;

case "CG80LengthChoice": _result_xjal = CG80LengthChoice; break;

case "CG100LengthChoice": _result_xjal = CG100LengthChoice; break;

case "CG20PVCChoice": _result_xjal = CG20PVCChoice; break;

case "CG40PVCChoice": _result_xjal = CG40PVCChoice; break;

case "CG60PVCChoice": _result_xjal = CG60PVCChoice; break;

case "CG80PVCChoice": _result_xjal = CG80PVCChoice; break;

case "CG100PVCChoice": _result_xjal = CG100PVCChoice; break;

case "RiskAttitudeChoice": _result_xjal = RiskAttitudeChoice; break;

case "CapitalImprovementChoice": _result_xjal = CapitalImprovementChoice; break;

case "DesiredAverageConditionChoice": _result_xjal = DesiredAverageConditionChoice; break;

case "BreakToleranceChoice": _result_xjal = BreakToleranceChoice; break;

case "AverageFlowRate": _result_xjal = AverageFlowRate; break;

case "AveragePressure": _result_xjal = AveragePressure; break;

case "AverageFailureDuration": _result_xjal = AverageFailureDuration; break;

case "EnergyIntensityWater": _result_xjal = EnergyIntensityWater; break;

case "DemandChoice": _result_xjal = DemandChoice; break;

case "pop_rateChoice": _result_xjal = pop_rateChoice; break;

case "waterRateChoice": _result_xjal = waterRateChoice; break;

default: _result_xjal = super.getParameter( _name_xjal ); break;

}

return (T) _result_xjal;

}

@AnyLogicInternalCodegenAPI

private static String[] _parameterNames_xjal;

@Override

public String[] getParameterNames() {

String[] result = _parameterNames_xjal;

if (result == null) {

List<String> list = new ArrayList<>( Arrays.asList( super.getParameterNames() ) );

list.add( "PopulationChoice" );

list.add( "NumberHouseholdsChoice" );

list.add( "InitialPriceChoice" );

list.add( "TotalPipeLength" );

list.add( "CG20LengthChoice" );

list.add( "CG40LengthChoice" );

list.add( "CG60LengthChoice" );

list.add( "CG80LengthChoice" );

list.add( "CG100LengthChoice" );

list.add( "CG20PVCChoice" );

list.add( "CG40PVCChoice" );

list.add( "CG60PVCChoice" );

list.add( "CG80PVCChoice" );

list.add( "CG100PVCChoice" );

list.add( "RiskAttitudeChoice" );

list.add( "CapitalImprovementChoice" );

list.add( "DesiredAverageConditionChoice" );

list.add( "BreakToleranceChoice" );

list.add( "AverageFlowRate" );

list.add( "AveragePressure" );

list.add( "AverageFailureDuration" );

list.add( "EnergyIntensityWater" );

list.add( "DemandChoice" );

list.add( "pop_rateChoice" );

list.add( "waterRateChoice" );

result = list.toArray( new String[ list.size() ] );

_parameterNames_xjal = result;

}

return result;

}

// Plain Variables

public

double

CumulativeDemand;

public

double

CumulativeSupply;

// Collection Variables

public

ArrayList <

Double > AllAvgConditions = new ArrayList<Double>();

public

ArrayList <

Double > AllAnnualBreaks = new ArrayList<Double>();

public

ArrayList <

Double > AnnualLeakageAmount = new ArrayList<Double>();

public

ArrayList <

Double > AnnualWaterLoss = new ArrayList<Double>();

public

ArrayList <

Double > AnnuaEnergyLoss = new ArrayList<Double>();

public

ArrayList <

Double > AverageReliability = new ArrayList<Double>();

@AnyLogicInternalCodegenAPI

private static Map<String, IElementDescriptor> elementDesciptors_xjal = null;

@AnyLogicInternalCodegenAPI

@Override

public Map<String, IElementDescriptor> getElementDesciptors() {

if (elementDesciptors_xjal == null) {

elementDesciptors_xjal = createElementDescriptors(super.getElementDesciptors(), Main.class);

}

return elementDesciptors_xjal;

}

@AnyLogicCustomProposalPriority(type = AnyLogicCustomProposalPriority.Type.STATIC_ELEMENT)

public static final Scale scale = new Scale( 10.0 );

@Override

public Scale getScale() {

return scale;

}

// Events

public EventTimeout yearlySaveAverage = new EventTimeout(this);

@AnyLogicInternalCodegenAPI

public EventTimeout _plot2_autoUpdateEvent_xjal = new EventTimeout(this);

@AnyLogicInternalCodegenAPI

public EventTimeout _plot3_autoUpdateEvent_xjal = new EventTimeout(this);

@AnyLogicInternalCodegenAPI

public EventTimeout _plot4_autoUpdateEvent_xjal = new EventTimeout(this);

@AnyLogicInternalCodegenAPI

public EventTimeout _plot_autoUpdateEvent_xjal = new EventTimeout(this);

@AnyLogicInternalCodegenAPI

public EventTimeout _plot1_autoUpdateEvent_xjal = new EventTimeout(this);

@Override

@AnyLogicInternalCodegenAPI

public String getNameOf( EventTimeout _e ) {

if( _e == yearlySaveAverage ) return "yearlySaveAverage";

if( _e == _plot2_autoUpdateEvent_xjal ) return "plot2 auto update event";

if( _e == _plot3_autoUpdateEvent_xjal ) return "plot3 auto update event";

if( _e == _plot4_autoUpdateEvent_xjal ) return "plot4 auto update event";

if( _e == _plot_autoUpdateEvent_xjal ) return "plot auto update event";

if( _e == _plot1_autoUpdateEvent_xjal ) return "plot1 auto update event";

return super.getNameOf( _e );

}

@Override

@AnyLogicInternalCodegenAPI

public EventTimeout.Mode getModeOf( EventTimeout _e ) {

if ( _e == yearlySaveAverage ) return EVENT_TIMEOUT_MODE_CYCLIC;

if ( _e == _plot2_autoUpdateEvent_xjal ) return EVENT_TIMEOUT_MODE_CYCLIC;

if ( _e == _plot3_autoUpdateEvent_xjal ) return EVENT_TIMEOUT_MODE_CYCLIC;

if ( _e == _plot4_autoUpdateEvent_xjal ) return EVENT_TIMEOUT_MODE_CYCLIC;

if ( _e == _plot_autoUpdateEvent_xjal ) return EVENT_TIMEOUT_MODE_CYCLIC;

if ( _e == _plot1_autoUpdateEvent_xjal ) return EVENT_TIMEOUT_MODE_CYCLIC;

return super.getModeOf( _e );

}

@Override

@AnyLogicInternalCodegenAPI

public double getFirstOccurrenceTime( EventTimeout _e ) {

double _t;

if ( _e == yearlySaveAverage ) {

_t =

1

;

_t = toModelTime( _t, YEAR );

return _t;

}

if ( _e == _plot2_autoUpdateEvent_xjal ) {

_t =

0

;

_t = toModelTime( _t, YEAR );

return _t;

}

if ( _e == _plot3_autoUpdateEvent_xjal ) {

_t =

0

;

_t = toModelTime( _t, YEAR );

return _t;

}

if ( _e == _plot4_autoUpdateEvent_xjal ) {

_t =

0

;

_t = toModelTime( _t, YEAR );

return _t;

}

if ( _e == _plot_autoUpdateEvent_xjal ) {

_t =

0

;

_t = toModelTime( _t, YEAR );

return _t;

}

if ( _e == _plot1_autoUpdateEvent_xjal ) {

_t =

0

;

_t = toModelTime( _t, YEAR );

return _t;

}

return super.getFirstOccurrenceTime( _e );

}

@Override

@AnyLogicInternalCodegenAPI

public double evaluateTimeoutOf( EventTimeout _e ) {

double _t;

if( _e == yearlySaveAverage) {

_t =

1

;

_t = toModelTime( _t, YEAR );

return _t;

}

if( _e == _plot2_autoUpdateEvent_xjal) {

_t =

1

;

_t = toModelTime( _t, YEAR );

return _t;

}

if( _e == _plot3_autoUpdateEvent_xjal) {

_t =

1

;

_t = toModelTime( _t, YEAR );

return _t;

}

if( _e == _plot4_autoUpdateEvent_xjal) {

_t =

1

;

_t = toModelTime( _t, YEAR );

return _t;

}

if( _e == _plot_autoUpdateEvent_xjal) {

_t =

1

;

_t = toModelTime( _t, YEAR );

return _t;

}

if( _e == _plot1_autoUpdateEvent_xjal) {

_t =

1

;

_t = toModelTime( _t, YEAR );

return _t;

}

return super.evaluateTimeoutOf( _e );

}

@Override

@AnyLogicInternalCodegenAPI

public void executeActionOf( EventTimeout _e ) {

if ( _e == yearlySaveAverage ) {

EventTimeout self = _e;

AllAvgConditions.add(pipe.AverageCondition());

AllAnnualBreaks.add(pipe.AnnualNetworkBreakage);

AnnualLeakageAmount.add(pipe.LeakageAmount);

AnnualWaterLoss.add(pipe.AnnualTotalWaterLoss);

AnnuaEnergyLoss.add(pipe.AnnualEnergyLoss_Mwh);

AverageReliability.add((CumulativeDemand/CumulativeSupply)*100);

;

return;

}

if ( _e == _plot2_autoUpdateEvent_xjal ) {

plot2.updateData();

return;

}

if ( _e == _plot3_autoUpdateEvent_xjal ) {

plot3.updateData();

return;

}

if ( _e == _plot4_autoUpdateEvent_xjal ) {

plot4.updateData();

return;

}

if ( _e == _plot_autoUpdateEvent_xjal ) {

plot.updateData();

return;

}

if ( _e == _plot1_autoUpdateEvent_xjal ) {

plot1.updateData();

return;

}

super.executeActionOf( _e );

}

// Embedded Objects

public Pipe pipe;

public User user;

public Agency agency;

public String getNameOf( Agent ao ) {

if ( ao == pipe ) return "pipe";

if ( ao == user ) return "user";

if ( ao == agency ) return "agency";

return super.getNameOf( ao );

}

public AgentAnimationSettings getAnimationSettingsOf( Agent ao ) {

return super.getAnimationSettingsOf( ao );

}

public String getNameOf( AgentList<?> aolist ) {

return super.getNameOf( aolist );

}

public AgentAnimationSettings getAnimationSettingsOf( AgentList<?> aolist ) {

return super.getAnimationSettingsOf( aolist );

}

/**

* Creates an embedded object instance<br>

* <i>This method should not be called by user</i>

*/

protected Pipe instantiate_pipe_xjal() {

Pipe _result_xjal = new Pipe( getEngine(), this, null );

return _result_xjal;

}

/**

* Setups parameters of an embedded object instance<br>

* This method should not be called by user

*/

private void setupParameters_pipe_xjal( final Pipe self ) {

setupParameters_pipe_xjal( self, null );

}

/**

* Setups an embedded object instance<br>

* This method should not be called by user

*/

@AnyLogicInternalCodegenAPI

private void create_pipe_xjal( Pipe self ) {

create_pipe_xjal(self, null );

}

/**

* Setups parameters of an embedded object instance<br>

* This method should not be called by user

*/

private void setupParameters_pipe_xjal( final Pipe self, TableInput _t ) {

self.RehabFraction =

0

;

self.TotalLengthPipeMile =

TotalPipeLength

;

}

/**

* Setups an embedded object instance<br>

* This method should not be called by user

*/

@AnyLogicInternalCodegenAPI

private void create_pipe_xjal( Pipe self, TableInput _t ) {

self.setEnvironment( this );

self.create();

}

/**

* Creates an embedded object instance<br>

* <i>This method should not be called by user</i>

*/

protected User instantiate_user_xjal() {

User _result_xjal = new User( getEngine(), this, null );

return _result_xjal;

}

/**

* Setups parameters of an embedded object instance<br>

* This method should not be called by user

*/

private void setupParameters_user_xjal( final User self ) {

setupParameters_user_xjal( self, null );

}

/**

* Setups an embedded object instance<br>

* This method should not be called by user

*/

@AnyLogicInternalCodegenAPI

private void create_user_xjal( User self ) {

create_user_xjal(self, null );

}

/**

* Setups parameters of an embedded object instance<br>

* This method should not be called by user

*/

private void setupParameters_user_xjal( final User self, TableInput _t ) {

self.BasePopulation =

PopulationChoice

;

self.NumberHouseholds =

NumberHouseholdsChoice

;

self.pop_rate = self._pop_rate_DefaultValue_xjal();

self.t = self._t_DefaultValue_xjal();

}

/**

* Setups an embedded object instance<br>

* This method should not be called by user

*/

@AnyLogicInternalCodegenAPI

private void create_user_xjal( User self, TableInput _t ) {

self.setEnvironment( this );

self.create();

}

/**

* Creates an embedded object instance<br>

* <i>This method should not be called by user</i>

*/

protected Agency instantiate_agency_xjal() {

Agency _result_xjal = new Agency( getEngine(), this, null );

return _result_xjal;

}

/**

* Setups parameters of an embedded object instance<br>

* This method should not be called by user

*/

private void setupParameters_agency_xjal( final Agency self ) {

setupParameters_agency_xjal( self, null );

}

/**

* Setups an embedded object instance<br>

* This method should not be called by user

*/

@AnyLogicInternalCodegenAPI

private void create_agency_xjal( Agency self ) {

create_agency_xjal(self, null );

}

/**

* Setups parameters of an embedded object instance<br>

* This method should not be called by user

*/

private void setupParameters_agency_xjal( final Agency self, TableInput _t ) {

self.TotalPipeLength =

TotalPipeLength

;

self.UnitPriceCapEx_$_Mile = self._UnitPriceCapEx_$_Mile_DefaultValue_xjal();

self.InputUserFees =

InitialPriceChoice

;

self.CapitalImprovement =

CapitalImprovementChoice

;

}

/**

* Setups an embedded object instance<br>

* This method should not be called by user

*/

@AnyLogicInternalCodegenAPI

private void create_agency_xjal( Agency self, TableInput _t ) {

self.setEnvironment( this );

self.create();

}

// Functions

double

LevelOfService( ) {

CumulativeDemand = CumulativeDemand + agency.AgencyWaterDemand;

CumulativeSupply = CumulativeSupply + agency.AgencyWaterSupply;

return CumulativeSupply/CumulativeDemand;

//return agency.AgencyWaterSupply/agency.AgencyWaterDemand;

}

/**

* Function for printing decimal numbers

*/

String

printf( double myvalue ) {

DecimalFormat df = new DecimalFormat("#");

df.setMaximumFractionDigits(2);

return df.format(myvalue);

}

String

ServiceReliability( ) {

double sr = ((CumulativeDemand == 0 || CumulativeSupply == 0) ? 0 : CumulativeSupply/CumulativeDemand);

return printf(sr*100);

}

int

average( ArrayList<Double> arr ) {

double sum = 0;

for(Double i : arr){

sum = i + sum;

}

return (int) (sum/(double)arr.size());

}

String

getRenewalStrategy( ) {

if(RiskAttitudeChoice == 0){

return "Control Break Frequency";

} else if(RiskAttitudeChoice == 1){

return "Control Average Condition";

}

else{

return "Regular Renewal";

}

}

@AnyLogicInternalCodegenAPI

public DataSet _plot2_expression0_dataSet_xjal = new DataSet( 100, new DataUpdater_xjal() {

double _lastUpdateX = Double.NaN;

@Override

public void update( DataSet _d ) {

if ( time() == _lastUpdateX ) { return; }

_d.add( time(), __plot2_expression0_dataSet_xjal_YValue() );

_lastUpdateX = time();

}

} );

/**

* <i>This method should not be called by user</i>

*/

@AnyLogicInternalCodegenAPI

private double __plot2_expression0_dataSet_xjal_YValue() {

return

pipe.LeakageAmount/1000000

;

}

@AnyLogicInternalCodegenAPI

public DataSet _plot3_expression0_dataSet_xjal = new DataSet( 100, new DataUpdater_xjal() {

double _lastUpdateX = Double.NaN;

@Override

public void update( DataSet _d ) {

if ( time() == _lastUpdateX ) { return; }

_d.add( time(), __plot3_expression0_dataSet_xjal_YValue() );

_lastUpdateX = time();

}

} );

/**

* <i>This method should not be called by user</i>

*/

@AnyLogicInternalCodegenAPI

private double __plot3_expression0_dataSet_xjal_YValue() {

return

pipe.AnnualNetworkBreakage

;

}

@AnyLogicInternalCodegenAPI

public DataSet _plot4_expression0_dataSet_xjal = new DataSet( 100, new DataUpdater_xjal() {

double _lastUpdateX = Double.NaN;

@Override

public void update( DataSet _d ) {

if ( time() == _lastUpdateX ) { return; }

_d.add( time(), __plot4_expression0_dataSet_xjal_YValue() );

_lastUpdateX = time();

}

} );

/**

* <i>This method should not be called by user</i>

*/

@AnyLogicInternalCodegenAPI

private double __plot4_expression0_dataSet_xjal_YValue() {

return

pipe.AnnualEnergyLoss_Mwh

;

}

@AnyLogicInternalCodegenAPI

public DataSet _plot_expression0_dataSet_xjal = new DataSet( 100, new DataUpdater_xjal() {

double _lastUpdateX = Double.NaN;

@Override

public void update( DataSet _d ) {

if ( time() == _lastUpdateX ) { return; }

_d.add( time(), __plot_expression0_dataSet_xjal_YValue() );

_lastUpdateX = time();

}

} );

/**

* <i>This method should not be called by user</i>

*/

@AnyLogicInternalCodegenAPI

private double __plot_expression0_dataSet_xjal_YValue() {

return

LevelOfService()*100

;

}

@AnyLogicInternalCodegenAPI

public DataSet _plot1_expression0_dataSet_xjal = new DataSet( 100, new DataUpdater_xjal() {

double _lastUpdateX = Double.NaN;

@Override

public void update( DataSet _d ) {

if ( time() == _lastUpdateX ) { return; }

_d.add( time(), __plot1_expression0_dataSet_xjal_YValue() );

_lastUpdateX = time();

}

} );

/**

* <i>This method should not be called by user</i>

*/

@AnyLogicInternalCodegenAPI

private double __plot1_expression0_dataSet_xjal_YValue() {

return

pipe.AverageCondition()

;

}

// View areas

public ViewArea _origin_VA = new ViewArea( this, "[Origin]", 0, 0, 1000.0, 590.0 );

@Override

@AnyLogicInternalCodegenAPI

public int getViewAreas(Map<String, ViewArea> _output) {

if ( _output != null ) {

_output.put( "_origin_VA", this._origin_VA );

}

return 1 + super.getViewAreas( _output );

}

@AnyLogicInternalCodegenAPI

protected static final Font _text1_Font = new Font("SansSerif", 1, 14 );

@AnyLogicInternalCodegenAPI

protected static final Font _text_Font = _text1_Font;

@AnyLogicInternalCodegenAPI

protected static final Font _text2_Font = _text1_Font;

@AnyLogicInternalCodegenAPI

protected static final Font _text3_Font = _text1_Font;

@AnyLogicInternalCodegenAPI

protected static final Font _text4_Font = _text1_Font;

@AnyLogicInternalCodegenAPI

protected static final Font _text5_Font = _text1_Font;

@AnyLogicInternalCodegenAPI

protected static final Font _text7_Font = _text1_Font;

@AnyLogicInternalCodegenAPI

protected static final int _text1 = 1;

@AnyLogicInternalCodegenAPI

protected static final int _text = 2;

@AnyLogicInternalCodegenAPI

protected static final int _text2 = 3;

@AnyLogicInternalCodegenAPI

protected static final int _text3 = 4;

@AnyLogicInternalCodegenAPI

protected static final int _text4 = 5;

@AnyLogicInternalCodegenAPI

protected static final int _text5 = 6;

@AnyLogicInternalCodegenAPI

protected static final int _text7 = 7;

@AnyLogicInternalCodegenAPI

protected static final int _plot2 = 8;

@AnyLogicInternalCodegenAPI

protected static final int _plot3 = 9;

@AnyLogicInternalCodegenAPI

protected static final int _plot4 = 10;

@AnyLogicInternalCodegenAPI

protected static final int _plot = 11;

@AnyLogicInternalCodegenAPI

protected static final int _plot1 = 12;

/** Internal constant, shouldn't be accessed by user */

@AnyLogicInternalCodegenAPI

protected static final int _SHAPE_NEXT_ID_xjal = 13;

/**

* Top-level presentation group id

*/

@AnyLogicInternalCodegenAPI

protected static final int _presentation = 0;

@AnyLogicInternalCodegenAPI

public boolean isPublicPresentationDefined() {

return true;

}

@AnyLogicInternalCodegenAPI

public boolean isEmbeddedAgentPresentationVisible( Agent _a ) {

return super.isEmbeddedAgentPresentationVisible( _a );

}

/**

* Top-level icon group id

*/

@AnyLogicInternalCodegenAPI

protected static final int _icon = -1;

protected TimePlot plot2;

protected TimePlot plot3;

protected TimePlot plot4;

protected TimePlot plot;

protected TimePlot plot1;

/**

* <i>This method should not be called by user</i>

*/

@AnyLogicInternalCodegenAPI

private void _text1_SetDynamicParams_xjal( ShapeText shape ) {

shape.setText(

ServiceReliability()

);

}

protected ShapeText text1;

/**

* <i>This method should not be called by user</i>

*/

@AnyLogicInternalCodegenAPI

private void _text_SetDynamicParams_xjal( ShapeText shape ) {

shape.setText(

(pipe.YearCounter < 100) ? "" : "Avg "+ average(AllAvgConditions)

);

}

protected ShapeText text;

/**

* <i>This method should not be called by user</i>

*/

@AnyLogicInternalCodegenAPI

private void _text2_SetDynamicParams_xjal( ShapeText shape ) {

shape.setText(

(pipe.YearCounter < 100) ? "" : "Avg "+ average(AnnuaEnergyLoss)

);

}

protected ShapeText text2;

/**

* <i>This method should not be called by user</i>

*/

@AnyLogicInternalCodegenAPI

private void _text3_SetDynamicParams_xjal( ShapeText shape ) {

shape.setText(

(pipe.YearCounter < 100) ? "" : "Avg "+ average(AnnualLeakageAmount)

);

}

protected ShapeText text3;

/**

* <i>This method should not be called by user</i>

*/

@AnyLogicInternalCodegenAPI

private void _text4_SetDynamicParams_xjal( ShapeText shape ) {

shape.setText(

(pipe.YearCounter < 100) ? "" : "Average Water Loss: \n "+average(AnnualWaterLoss)

);

}

protected ShapeText text4;

/**

* <i>This method should not be called by user</i>

*/

@AnyLogicInternalCodegenAPI

private void _text5_SetDynamicParams_xjal( ShapeText shape ) {

shape.setText(

(pipe.YearCounter < 100) ? "" : "Avg "+ average(AllAnnualBreaks)

);

}

protected ShapeText text5;

/**

* <i>This method should not be called by user</i>

*/

@AnyLogicInternalCodegenAPI

private void _text7_SetDynamicParams_xjal( ShapeText shape ) {

shape.setText(

"Service Relibility: "+ServiceReliability()

);

}

protected ShapeText text7;

@AnyLogicInternalCodegenAPI

private void _createPersistentElementsBP0_xjal() {

text1 = new ShapeText(

SHAPE_DRAW_2D, true,920.0, 390.0, 0.0, 0.0,

peru,"text",

_text1_Font, ALIGNMENT_LEFT ) {

@Override

public void updateDynamicProperties(boolean publicOnly) {

_text1_SetDynamicParams_xjal( this );

super.updateDynamicProperties(publicOnly);

}

};

text = new ShapeText(

SHAPE_DRAW_2D, true,50.0, 660.0, 0.0, 0.0,

teal,"text",

_text_Font, ALIGNMENT_LEFT ) {

@Override

public void updateDynamicProperties(boolean publicOnly) {

_text_SetDynamicParams_xjal( this );

super.updateDynamicProperties(publicOnly);

}

};

text2 = new ShapeText(

SHAPE_DRAW_2D, true,-430.0, 380.0, 0.0, 0.0,

gold,"text",

_text2_Font, ALIGNMENT_LEFT ) {

@Override

public void updateDynamicProperties(boolean publicOnly) {

_text2_SetDynamicParams_xjal( this );

super.updateDynamicProperties(publicOnly);

}

};

text3 = new ShapeText(

SHAPE_DRAW_2D, true,50.0, 620.0, 0.0, 0.0,

yellowGreen,"text",

_text3_Font, ALIGNMENT_LEFT ) {

@Override

public void updateDynamicProperties(boolean publicOnly) {

_text3_SetDynamicParams_xjal( this );

super.updateDynamicProperties(publicOnly);

}

};

text4 = new ShapeText(

SHAPE_DRAW_2D, true,220.0, 700.0, 0.0, 0.0,

dodgerBlue,"text",

_text4_Font, ALIGNMENT_LEFT ) {

@Override

public void updateDynamicProperties(boolean publicOnly) {

_text4_SetDynamicParams_xjal( this );

super.updateDynamicProperties(publicOnly);

}

};

text5 = new ShapeText(

SHAPE_DRAW_2D, true,120.0, 630.0, 0.0, 0.0,

brown,"text",

_text5_Font, ALIGNMENT_LEFT ) {

@Override

public void updateDynamicProperties(boolean publicOnly) {

_text5_SetDynamicParams_xjal( this );

super.updateDynamicProperties(publicOnly);

}

};

text7 = new ShapeText(

SHAPE_DRAW_2D, true,180.0, 650.0, 0.0, 0.0,

peru,"text",

_text7_Font, ALIGNMENT_LEFT ) {

@Override

public void updateDynamicProperties(boolean publicOnly) {

_text7_SetDynamicParams_xjal( this );

super.updateDynamicProperties(publicOnly);

}

};

}

@AnyLogicInternalCodegenAPI

private void _createPersistentElementsAP0_xjal() {

{

DataSet _item;

List<DataSet> _items = new ArrayList<DataSet>( 1 );

_items.add( _plot2_expression0_dataSet_xjal );

List<String> _titles = new ArrayList<String>( 1 );

_titles.add( "Amount of Annual Leakage (Mgall)" );

List<Chart2DPlot.Appearance> _appearances = new ArrayList<Chart2DPlot.Appearance>( 1 );

_appearances.add( new Chart2DPlot.Appearance( limeGreen, true, Chart.INTERPOLATION_LINEAR, 1.0, Chart.POINT_NONE ) );

plot2 = new TimePlot(

Main.this, true, 10.0, 210.0,

380.0, 210.0,

null, null,

80.0, 30.0,

270.0, 120.0, white, black, black,

30.0, Chart.SOUTH,

100

, Chart.WINDOW_MOVES_WITH_TIME, null, Chart.SCALE_AUTO,

0, 0, Chart.GRID_DEFAULT, Chart.GRID_DEFAULT,

darkGray, darkGray, _items, _titles, _appearances );

}

{

DataSet _item;

List<DataSet> _items = new ArrayList<DataSet>( 1 );

_items.add( _plot3_expression0_dataSet_xjal );

List<String> _titles = new ArrayList<String>( 1 );

_titles.add( "Number of Annual Breaks" );

List<Chart2DPlot.Appearance> _appearances = new ArrayList<Chart2DPlot.Appearance>( 1 );

_appearances.add( new Chart2DPlot.Appearance( brown, true, Chart.INTERPOLATION_LINEAR, 1.0, Chart.POINT_NONE ) );

plot3 = new TimePlot(

Main.this, true, 330.0, 210.0,

360.0, 210.0,

null, null,

80.0, 30.0,

250.0, 120.0, white, black, black,

30.0, Chart.SOUTH,

100

, Chart.WINDOW_MOVES_WITH_TIME, null, Chart.SCALE_AUTO,

0, 0, Chart.GRID_DEFAULT, Chart.GRID_DEFAULT,

darkGray, darkGray, _items, _titles, _appearances );

}

{

DataSet _item;

List<DataSet> _items = new ArrayList<DataSet>( 1 );

_items.add( _plot4_expression0_dataSet_xjal );

List<String> _titles = new ArrayList<String>( 1 );

_titles.add( "Amount of Annual Energy Loss (Mwh)" );

List<Chart2DPlot.Appearance> _appearances = new ArrayList<Chart2DPlot.Appearance>( 1 );

_appearances.add( new Chart2DPlot.Appearance( gold, true, Chart.INTERPOLATION_LINEAR, 2.0, Chart.POINT_NONE ) );

plot4 = new TimePlot(

Main.this, true, -570.0, 160.0,

360.0, 210.0,

null, null,

80.0, 30.0,

240.0, 120.0, white, black, black,

30.0, Chart.SOUTH,

100

, Chart.WINDOW_MOVES_WITH_TIME, null, Chart.SCALE_AUTO,

0, 0, Chart.GRID_DEFAULT, Chart.GRID_DEFAULT,

darkGray, darkGray, _items, _titles, _appearances );

}

{

DataSet _item;

List<DataSet> _items = new ArrayList<DataSet>( 1 );

_items.add( _plot_expression0_dataSet_xjal );

List<String> _titles = new ArrayList<String>( 1 );

_titles.add( "Service Reliability (%)" );

List<Chart2DPlot.Appearance> _appearances = new ArrayList<Chart2DPlot.Appearance>( 1 );

_appearances.add( new Chart2DPlot.Appearance( peru, true, Chart.INTERPOLATION_LINEAR, 2.0, Chart.POINT_NONE ) );

plot = new TimePlot(

Main.this, true, 680.0, 210.0,

320.0, 210.0,

null, null,

50.0, 30.0,

240.0, 120.0, white, black, black,

30.0, Chart.SOUTH,

100

, Chart.WINDOW_MOVES_WITH_TIME, null, Chart.SCALE_AUTO,

0, 0, Chart.GRID_DEFAULT, Chart.GRID_DEFAULT,

darkGray, darkGray, _items, _titles, _appearances );

}

{

DataSet _item;

List<DataSet> _items = new ArrayList<DataSet>( 1 );

_items.add( _plot1_expression0_dataSet_xjal );

List<String> _titles = new ArrayList<String>( 1 );

_titles.add( "Average Condition " );

List<Chart2DPlot.Appearance> _appearances = new ArrayList<Chart2DPlot.Appearance>( 1 );

_appearances.add( new Chart2DPlot.Appearance( teal, true, Chart.INTERPOLATION_LINEAR, 2.0, Chart.POINT_NONE ) );

plot1 = new TimePlot(

Main.this, true, 680.0, 10.0,

320.0, 210.0,

null, null,

50.0, 30.0,

240.0, 120.0, white, black, black,

30.0, Chart.SOUTH,

100

, Chart.WINDOW_MOVES_WITH_TIME, null, Chart.SCALE_AUTO,

0, 0, Chart.GRID_DEFAULT, Chart.GRID_DEFAULT,

darkGray, darkGray, _items, _titles, _appearances );

}

}

// Static initialization of persistent elements

{

_createPersistentElementsBP0_xjal();

}

protected ShapeTopLevelPresentationGroup presentation;

protected ShapeGroup icon;

@Override

@AnyLogicInternalCodegenAPI

public Object getPersistentShape( int _shape ) {

switch (_shape) {

case _presentation: return presentation;

case _icon: return icon;

case _plot2: return plot2;

case _plot3: return plot3;

case _plot4: return plot4;

case _plot: return plot;

case _plot1: return plot1;

case _text1: return text1;

case _text: return text;

case _text2: return text2;

case _text3: return text3;

case _text4: return text4;

case _text5: return text5;

case _text7: return text7;

default: return super.getPersistentShape( _shape );

}

}

@Override

@AnyLogicInternalCodegenAPI

public String getNameOfShape_xjal( Object _shape ) {

try {

if ( _shape == null ) return null;

String _name_xjal;

_name_xjal = checkNameOfShape_xjal( _shape, presentation, "presentation" ); if (_name_xjal != null) return _name_xjal;

_name_xjal = checkNameOfShape_xjal( _shape, icon, "icon" ); if (_name_xjal != null) return _name_xjal;

_name_xjal = checkNameOfShape_xjal( _shape, plot2, "plot2" ); if (_name_xjal != null) return _name_xjal;

_name_xjal = checkNameOfShape_xjal( _shape, plot3, "plot3" ); if (_name_xjal != null) return _name_xjal;

_name_xjal = checkNameOfShape_xjal( _shape, plot4, "plot4" ); if (_name_xjal != null) return _name_xjal;

_name_xjal = checkNameOfShape_xjal( _shape, plot, "plot" ); if (_name_xjal != null) return _name_xjal;

_name_xjal = checkNameOfShape_xjal( _shape, plot1, "plot1" ); if (_name_xjal != null) return _name_xjal;

_name_xjal = checkNameOfShape_xjal( _shape, text1, "text1" ); if (_name_xjal != null) return _name_xjal;

_name_xjal = checkNameOfShape_xjal( _shape, text, "text" ); if (_name_xjal != null) return _name_xjal;

_name_xjal = checkNameOfShape_xjal( _shape, text2, "text2" ); if (_name_xjal != null) return _name_xjal;

_name_xjal = checkNameOfShape_xjal( _shape, text3, "text3" ); if (_name_xjal != null) return _name_xjal;

_name_xjal = checkNameOfShape_xjal( _shape, text4, "text4" ); if (_name_xjal != null) return _name_xjal;

_name_xjal = checkNameOfShape_xjal( _shape, text5, "text5" ); if (_name_xjal != null) return _name_xjal;

_name_xjal = checkNameOfShape_xjal( _shape, text7, "text7" ); if (_name_xjal != null) return _name_xjal;

} catch (Exception e) {

return null;

}

return super.getNameOfShape_xjal( _shape );

}

@AnyLogicInternalCodegenAPI

private void drawModelElements_Events_xjal(Panel _panel, Graphics2D _g, boolean _publicOnly, boolean _isSuperClass ) {

if (!_publicOnly) {

drawEvent( _panel, _g, 300, -210, 10, 0, "yearlySaveAverage", yearlySaveAverage );

}

}

@AnyLogicInternalCodegenAPI

private void drawModelElements_Parameters_xjal(Panel _panel, Graphics2D _g, boolean _publicOnly, boolean _isSuperClass ) {

if (!_publicOnly) {

drawParameter( _panel, _g, -170, -90, 10, 0, "PopulationChoice", PopulationChoice, 0 );

}

if (!_publicOnly) {

drawParameter( _panel, _g, -170, -40, 10, 0, "NumberHouseholdsChoice", NumberHouseholdsChoice, 0 );

}

if (!_publicOnly) {

drawParameter( _panel, _g, -170, -10, 10, 0, "InitialPriceChoice", InitialPriceChoice, 0 );

}

if (!_publicOnly) {

drawParameter( _panel, _g, -170, -120, 10, 0, "TotalPipeLength", TotalPipeLength, 0 );

}

if (!_publicOnly) {

drawParameter( _panel, _g, -210, 130, 10, 0, "CG20LengthChoice", CG20LengthChoice, 0 );

}

if (!_publicOnly) {

drawParameter( _panel, _g, -210, 160, 10, 0, "CG40LengthChoice", CG40LengthChoice, 0 );

}

if (!_publicOnly) {

drawParameter( _panel, _g, -210, 190, 10, 0, "CG60LengthChoice", CG60LengthChoice, 0 );

}

if (!_publicOnly) {

drawParameter( _panel, _g, -210, 220, 10, 0, "CG80LengthChoice", CG80LengthChoice, 0 );

}

if (!_publicOnly) {

drawParameter( _panel, _g, -210, 250, 10, 0, "CG100LengthChoice", CG100LengthChoice, 0 );

}

if (!_publicOnly) {

drawParameter( _panel, _g, -160, 320, 10, 0, "CG20PVCChoice", CG20PVCChoice, 0 );

}

if (!_publicOnly) {

drawParameter( _panel, _g, -160, 350, 10, 0, "CG40PVCChoice", CG40PVCChoice, 0 );

}

if (!_publicOnly) {

drawParameter( _panel, _g, -160, 380, 10, 0, "CG60PVCChoice", CG60PVCChoice, 0 );

}

if (!_publicOnly) {

drawParameter( _panel, _g, -160, 410, 10, 0, "CG80PVCChoice", CG80PVCChoice, 0 );

}

if (!_publicOnly) {

drawParameter( _panel, _g, -160, 440, 10, 0, "CG100PVCChoice", CG100PVCChoice, 0 );

}

if (!_publicOnly) {

drawParameter( _panel, _g, -170, -150, 10, 0, "RiskAttitudeChoice", RiskAttitudeChoice, 0 );

}

if (!_publicOnly) {

drawParameter( _panel, _g, -170, -180, 10, 0, "CapitalImprovementChoice", CapitalImprovementChoice, 0 );

}

if (!_publicOnly) {

drawParameter( _panel, _g, -170, -210, 10, 0, "DesiredAverageConditionChoice", DesiredAverageConditionChoice, 0 );

}

if (!_publicOnly) {

drawParameter( _panel, _g, -170, -240, 10, 0, "BreakToleranceChoice", BreakToleranceChoice, 0 );

}

if (!_publicOnly) {

drawParameter( _panel, _g, -170, -330, 10, 0, "AverageFlowRate", AverageFlowRate, 0 );

}

if (!_publicOnly) {

drawParameter( _panel, _g, -170, -300, 10, 0, "AveragePressure", AveragePressure, 0 );

}

if (!_publicOnly) {

drawParameter( _panel, _g, -170, -270, 10, 0, "AverageFailureDuration", AverageFailureDuration, 0 );

}

if (!_publicOnly) {

drawParameter( _panel, _g, -170, -360, 10, 0, "EnergyIntensityWater", EnergyIntensityWater, 0 );

}

if (!_publicOnly) {

drawParameter( _panel, _g, -170, -390, 10, 0, "DemandChoice", DemandChoice, 0 );

}

if (!_publicOnly) {

drawParameter( _panel, _g, -170, -70, 10, 0, "pop_rateChoice", pop_rateChoice, 0 );

}

if (!_publicOnly) {

drawParameter( _panel, _g, -170, 20, 10, 0, "waterRateChoice", waterRateChoice, 0 );

}

}

@AnyLogicInternalCodegenAPI

private void drawModelElements_PlainVariables_xjal(Panel _panel, Graphics2D _g, boolean _publicOnly, boolean _isSuperClass ) {

if (!_publicOnly) {

drawPlainVariable( _panel, _g, -160, 540, 10, 0, "CumulativeDemand", CumulativeDemand, false );

}

if (!_publicOnly) {

drawPlainVariable( _panel, _g, -160, 570, 10, 0, "CumulativeSupply", CumulativeSupply, false );

}

}

@AnyLogicInternalCodegenAPI

private void drawModelElements_CollectionVariables_xjal(Panel _panel, Graphics2D _g, boolean _publicOnly, boolean _isSuperClass ) {

if (!_publicOnly) {

drawCollection( _panel, _g, 140, -190, 15, 0, "AllAvgConditions", AllAvgConditions );

}

if (!_publicOnly) {

drawCollection( _panel, _g, 140, -220, 15, 0, "AllAnnualBreaks", AllAnnualBreaks );

}

if (!_publicOnly) {

drawCollection( _panel, _g, 140, -160, 15, 0, "AnnualLeakageAmount", AnnualLeakageAmount );

}

if (!_publicOnly) {

drawCollection( _panel, _g, 140, -130, 15, 0, "AnnualWaterLoss", AnnualWaterLoss );

}

if (!_publicOnly) {

drawCollection( _panel, _g, 140, -100, 15, 0, "AnnuaEnergyLoss", AnnuaEnergyLoss );

}

if (!_publicOnly) {

drawCollection( _panel, _g, 140, -70, 15, 0, "AverageReliability", AverageReliability );

}

}

@AnyLogicInternalCodegenAPI

private void drawModelElements_Functions_xjal(Panel _panel, Graphics2D _g, boolean _publicOnly, boolean _isSuperClass ) {

if (!_publicOnly) {

drawFunction( _panel, _g, -160, 480, 10, 0, "LevelOfService");

}

if (!_publicOnly) {

drawFunction( _panel, _g, -170, 50, 10, 0, "printf");

}

if (!_publicOnly) {

drawFunction( _panel, _g, -160, 610, 10, 0, "ServiceReliability");

}

if (!_publicOnly) {

drawFunction( _panel, _g, 50, -220, 10, 0, "average");

}

if (!_publicOnly) {

drawFunction( _panel, _g, 50, -250, 10, 0, "getRenewalStrategy");

}

}

@AnyLogicInternalCodegenAPI

private void drawModelElements_EmbeddeObjects_xjal(Panel _panel, Graphics2D _g, boolean _publicOnly, boolean _isSuperClass ) {

// Embedded object "pipe"

if (!_publicOnly) {

drawEmbeddedObjectModel( _panel, _g, 30 , -100 , -60, 135, null, this.pipe );

}

// Embedded object "user"

if (!_publicOnly) {

drawEmbeddedObjectModelDefault( _panel, _g, -160 , 70 , 15, 0, "user", this.user );

}

// Embedded object "agency"

if (!_publicOnly) {

drawEmbeddedObjectModelDefault( _panel, _g, -160 , 90 , 15, 0, "agency", this.agency );

}

}

@AnyLogicInternalCodegenAPI

private void drawModelElements_AgentLinks_xjal(Panel _panel, Graphics2D _g, boolean _publicOnly, boolean _isSuperClass ) {

if (_publicOnly) { return; }

drawLinkToAgent( _panel, _g, 50, -50, 15, 0, "connections", true, connections );

}

@Override

@AnyLogicInternalCodegenAPI

public void drawModelElements( Panel _panel, Graphics2D _g, boolean _publicOnly, boolean _isSuperClass ) {

super.drawModelElements( _panel, _g, _publicOnly, true );

drawModelElements_Events_xjal( _panel, _g, _publicOnly, _isSuperClass );

drawModelElements_Parameters_xjal( _panel, _g, _publicOnly, _isSuperClass );

drawModelElements_PlainVariables_xjal( _panel, _g, _publicOnly, _isSuperClass );

drawModelElements_CollectionVariables_xjal( _panel, _g, _publicOnly, _isSuperClass );

drawModelElements_Functions_xjal( _panel, _g, _publicOnly, _isSuperClass );

drawModelElements_EmbeddeObjects_xjal( _panel, _g, _publicOnly, _isSuperClass );

drawModelElements_AgentLinks_xjal( _panel, _g, _publicOnly, _isSuperClass );

}

@AnyLogicInternalCodegenAPI

private boolean onClickModelAt_EmbeddedObjects_xjal( Panel _panel, double _x, double _y, int _clickCount, boolean _publicOnly, boolean _isSuperClass ) {

if ( pipe.onClickIconAt( _x - 30, _y - -100, true ) ) {

if ( _clickCount == 2 ) {

_panel.browseAgent_xjal( _x, _y, this, "pipe" );

} else {

_panel.addInspect( _x, _y, this, "pipe" );

}

return true;

}

if ( modelElementContains(_x, _y, -160, 70) ) {

if ( _clickCount == 2 ) {

_panel.browseAgent_xjal( -160, 70, this, "user" );

} else {

_panel.addInspect( -160, 70, this, "user" );

}

return true;

}

if ( modelElementContains(_x, _y, -160, 90) ) {

if ( _clickCount == 2 ) {

_panel.browseAgent_xjal( -160, 90, this, "agency" );

} else {

_panel.addInspect( -160, 90, this, "agency" );

}

return true;

}

return false;

}

@AnyLogicInternalCodegenAPI

private boolean onClickModelAt_AgentLinks_xjal( Panel _panel, double _x, double _y, int _clickCount, boolean _publicOnly, boolean _isSuperClass ) {

if ( modelElementContains(_x, _y, 50, -50) ) {

_panel.addInspect_xjal( 50, -50, this, "connections", Panel.INSPECT_CONNECTIONS_xjal );

return true;

}

return false;

}

@AnyLogicInternalCodegenAPI

private boolean onClickModelAt_Parameters_xjal( Panel _panel, double _x, double _y, int _clickCount, boolean _publicOnly, boolean _isSuperClass ) {

if( !_publicOnly && modelElementContains(_x, _y, -170, -90) ) {

_panel.addInspect( -170, -90, this, "PopulationChoice" );

return true;

}

if( !_publicOnly && modelElementContains(_x, _y, -170, -40) ) {

_panel.addInspect( -170, -40, this, "NumberHouseholdsChoice" );

return true;

}

if( !_publicOnly && modelElementContains(_x, _y, -170, -10) ) {

_panel.addInspect( -170, -10, this, "InitialPriceChoice" );

return true;

}

if( !_publicOnly && modelElementContains(_x, _y, -170, -120) ) {

_panel.addInspect( -170, -120, this, "TotalPipeLength" );

return true;

}

if( !_publicOnly && modelElementContains(_x, _y, -210, 130) ) {

_panel.addInspect( -210, 130, this, "CG20LengthChoice" );

return true;

}

if( !_publicOnly && modelElementContains(_x, _y, -210, 160) ) {

_panel.addInspect( -210, 160, this, "CG40LengthChoice" );

return true;

}

if( !_publicOnly && modelElementContains(_x, _y, -210, 190) ) {

_panel.addInspect( -210, 190, this, "CG60LengthChoice" );

return true;

}

if( !_publicOnly && modelElementContains(_x, _y, -210, 220) ) {

_panel.addInspect( -210, 220, this, "CG80LengthChoice" );

return true;

}

if( !_publicOnly && modelElementContains(_x, _y, -210, 250) ) {

_panel.addInspect( -210, 250, this, "CG100LengthChoice" );

return true;

}

if( !_publicOnly && modelElementContains(_x, _y, -160, 320) ) {

_panel.addInspect( -160, 320, this, "CG20PVCChoice" );

return true;

}

if( !_publicOnly && modelElementContains(_x, _y, -160, 350) ) {

_panel.addInspect( -160, 350, this, "CG40PVCChoice" );

return true;

}

if( !_publicOnly && modelElementContains(_x, _y, -160, 380) ) {

_panel.addInspect( -160, 380, this, "CG60PVCChoice" );

return true;

}

if( !_publicOnly && modelElementContains(_x, _y, -160, 410) ) {

_panel.addInspect( -160, 410, this, "CG80PVCChoice" );

return true;

}

if( !_publicOnly && modelElementContains(_x, _y, -160, 440) ) {

_panel.addInspect( -160, 440, this, "CG100PVCChoice" );

return true;

}

if( !_publicOnly && modelElementContains(_x, _y, -170, -150) ) {

_panel.addInspect( -170, -150, this, "RiskAttitudeChoice" );

return true;

}

if( !_publicOnly && modelElementContains(_x, _y, -170, -180) ) {

_panel.addInspect( -170, -180, this, "CapitalImprovementChoice" );

return true;

}

if( !_publicOnly && modelElementContains(_x, _y, -170, -210) ) {

_panel.addInspect( -170, -210, this, "DesiredAverageConditionChoice" );

return true;

}

if( !_publicOnly && modelElementContains(_x, _y, -170, -240) ) {

_panel.addInspect( -170, -240, this, "BreakToleranceChoice" );

return true;

}

if( !_publicOnly && modelElementContains(_x, _y, -170, -330) ) {

_panel.addInspect( -170, -330, this, "AverageFlowRate" );

return true;

}

if( !_publicOnly && modelElementContains(_x, _y, -170, -300) ) {

_panel.addInspect( -170, -300, this, "AveragePressure" );

return true;

}

if( !_publicOnly && modelElementContains(_x, _y, -170, -270) ) {

_panel.addInspect( -170, -270, this, "AverageFailureDuration" );

return true;

}

if( !_publicOnly && modelElementContains(_x, _y, -170, -360) ) {

_panel.addInspect( -170, -360, this, "EnergyIntensityWater" );

return true;

}

if( !_publicOnly && modelElementContains(_x, _y, -170, -390) ) {

_panel.addInspect( -170, -390, this, "DemandChoice" );

return true;

}

if( !_publicOnly && modelElementContains(_x, _y, -170, -70) ) {

_panel.addInspect( -170, -70, this, "pop_rateChoice" );

return true;

}

if( !_publicOnly && modelElementContains(_x, _y, -170, 20) ) {

_panel.addInspect( -170, 20, this, "waterRateChoice" );

return true;

}

return false;

}

@AnyLogicInternalCodegenAPI

private boolean onClickModelAt_PlainVariables_xjal( Panel _panel, double _x, double _y, int _clickCount, boolean _publicOnly, boolean _isSuperClass ) {

if( !_publicOnly && modelElementContains(_x, _y, -160, 540) ) {

_panel.addInspect( -160, 540, this, "CumulativeDemand" );

return true;

}

if( !_publicOnly && modelElementContains(_x, _y, -160, 570) ) {

_panel.addInspect( -160, 570, this, "CumulativeSupply" );

return true;

}

return false;

}

@AnyLogicInternalCodegenAPI

private boolean onClickModelAt_CollectionVariables_xjal( Panel _panel, double _x, double _y, int _clickCount, boolean _publicOnly, boolean _isSuperClass ) {

if( !_publicOnly && modelElementContains(_x, _y, 140, -190) ) {

_panel.addInspect( 140, -190, this, "AllAvgConditions" );

return true;

}

if( !_publicOnly && modelElementContains(_x, _y, 140, -220) ) {

_panel.addInspect( 140, -220, this, "AllAnnualBreaks" );

return true;

}

if( !_publicOnly && modelElementContains(_x, _y, 140, -160) ) {

_panel.addInspect( 140, -160, this, "AnnualLeakageAmount" );

return true;

}

if( !_publicOnly && modelElementContains(_x, _y, 140, -130) ) {

_panel.addInspect( 140, -130, this, "AnnualWaterLoss" );

return true;

}

if( !_publicOnly && modelElementContains(_x, _y, 140, -100) ) {

_panel.addInspect( 140, -100, this, "AnnuaEnergyLoss" );

return true;

}

if( !_publicOnly && modelElementContains(_x, _y, 140, -70) ) {

_panel.addInspect( 140, -70, this, "AverageReliability" );

return true;

}

return false;

}

@AnyLogicInternalCodegenAPI

private boolean onClickModelAt_Events_xjal( Panel _panel, double _x, double _y, int _clickCount, boolean _publicOnly, boolean _isSuperClass ) {

if( !_publicOnly && modelElementContains(_x, _y, 300, -210) ) {

_panel.addInspect( 300, -210, this, "yearlySaveAverage" );

return true;

}

return false;

}

@Override

@AnyLogicInternalCodegenAPI

public boolean onClickModelAt( Panel _panel, double _x, double _y, int _clickCount, boolean _publicOnly, boolean _isSuperClass ) {

if ( onClickModelAt_EmbeddedObjects_xjal( _panel, _x, _y, _clickCount, _publicOnly, _isSuperClass ) ) { return true; }

if ( onClickModelAt_AgentLinks_xjal( _panel, _x, _y, _clickCount, _publicOnly, _isSuperClass ) ) { return true; }

if ( onClickModelAt_Parameters_xjal( _panel, _x, _y, _clickCount, _publicOnly, _isSuperClass ) ) { return true; }

if ( onClickModelAt_PlainVariables_xjal( _panel, _x, _y, _clickCount, _publicOnly, _isSuperClass ) ) { return true; }

if ( onClickModelAt_CollectionVariables_xjal( _panel, _x, _y, _clickCount, _publicOnly, _isSuperClass ) ) { return true; }

if ( onClickModelAt_Events_xjal( _panel, _x, _y, _clickCount, _publicOnly, _isSuperClass ) ) { return true; }

return super.onClickModelAt( _panel, _x, _y, _clickCount, _publicOnly, true );

}

/**

* Constructor

*/

public Main( Engine engine, Agent owner, AgentList<? extends Main> ownerPopulation ) {

super( engine, owner, ownerPopulation );

instantiateBaseStructureThis_xjal();

}

@AnyLogicInternalCodegenAPI

public void onOwnerChanged_xjal() {

super.onOwnerChanged_xjal();

setupReferences_xjal();

}

@AnyLogicInternalCodegenAPI

public void instantiateBaseStructure_xjal() {

super.instantiateBaseStructure_xjal();

instantiateBaseStructureThis_xjal();

}

@AnyLogicInternalCodegenAPI

private void instantiateBaseStructureThis_xjal() {

pipe = instantiate_pipe_xjal();

user = instantiate_user_xjal();

agency = instantiate_agency_xjal();

setupReferences_xjal();

}

@AnyLogicInternalCodegenAPI

private void setupReferences_xjal() {

}

/**

* Simple constructor. Please add created agent to some population by calling goToPopulation() function

*/

public Main() {

}

/**

* Simple constructor. Please add created agent to some population by calling goToPopulation() function

*/

public Main( int PopulationChoice, int NumberHouseholdsChoice, double InitialPriceChoice, double TotalPipeLength, double CG20LengthChoice, double CG40LengthChoice, double CG60LengthChoice, double CG80LengthChoice, double CG100LengthChoice, double CG20PVCChoice, double CG40PVCChoice, double CG60PVCChoice, double CG80PVCChoice, double CG100PVCChoice, int RiskAttitudeChoice, double CapitalImprovementChoice, double DesiredAverageConditionChoice, int BreakToleranceChoice, double AverageFlowRate, double AveragePressure, double AverageFailureDuration, double EnergyIntensityWater, double DemandChoice, double pop_rateChoice, double waterRateChoice ) {

markParametersAreSet();

this.PopulationChoice = PopulationChoice;

this.NumberHouseholdsChoice = NumberHouseholdsChoice;

this.InitialPriceChoice = InitialPriceChoice;

this.TotalPipeLength = TotalPipeLength;

this.CG20LengthChoice = CG20LengthChoice;

this.CG40LengthChoice = CG40LengthChoice;

this.CG60LengthChoice = CG60LengthChoice;

this.CG80LengthChoice = CG80LengthChoice;

this.CG100LengthChoice = CG100LengthChoice;

this.CG20PVCChoice = CG20PVCChoice;

this.CG40PVCChoice = CG40PVCChoice;

this.CG60PVCChoice = CG60PVCChoice;

this.CG80PVCChoice = CG80PVCChoice;

this.CG100PVCChoice = CG100PVCChoice;

this.RiskAttitudeChoice = RiskAttitudeChoice;

this.CapitalImprovementChoice = CapitalImprovementChoice;

this.DesiredAverageConditionChoice = DesiredAverageConditionChoice;

this.BreakToleranceChoice = BreakToleranceChoice;

this.AverageFlowRate = AverageFlowRate;

this.AveragePressure = AveragePressure;

this.AverageFailureDuration = AverageFailureDuration;

this.EnergyIntensityWater = EnergyIntensityWater;

this.DemandChoice = DemandChoice;

this.pop_rateChoice = pop_rateChoice;

this.waterRateChoice = waterRateChoice;

}

/**

* Creating embedded object instances

*/

@AnyLogicInternalCodegenAPI

private void instantiatePopulations_xjal() {

}

@Override

@AnyLogicInternalCodegenAPI

public void doCreate() {

super.doCreate();

// Creating embedded object instances

instantiatePopulations_xjal();

// Assigning initial values for plain variables

setupPlainVariables_Main_xjal();

// Dynamic initialization of persistent elements

_createPersistentElementsAP0_xjal();

presentation = new ShapeTopLevelPresentationGroup( Main.this, true, 0, 0, 0, 0 , text1, text, text2, text3, text4, text5, text7, plot2, plot3, plot4, plot, plot1 );

icon = new ShapeGroup( Main.this, true, 0, 0, 0 );

// Creating embedded object instances

instantiatePopulations_xjal();

// Environments setup

{

double _x_xjal =

500

;

double _y_xjal =

500

;

double _z_xjal =

0

;

setupSpace( _x_xjal, _y_xjal, _z_xjal );

}

disableSteps();

setNetworkUserDefined();

setLayoutType( LAYOUT_USER_DEFINED );

// Creating non-replicated embedded objects

setupParameters_pipe_xjal( pipe );

create_pipe_xjal( pipe );

setupParameters_user_xjal( user );

create_user_xjal( user );

setupParameters_agency_xjal( agency );

create_agency_xjal( agency );

// Port connectors with non-replicated objects

// Creating replicated embedded objects

setupInitialConditions_xjal( Main.class );

}

@AnyLogicInternalCodegenAPI

public void setupExt_xjal(AgentExtension _ext) {

// Agent properties setup

if ( _ext instanceof ExtAgentWithSpatialMetrics && _ext instanceof ExtWithSpaceType ) {

double _value;

_value =

10

;

((ExtAgentWithSpatialMetrics) _ext).setSpeed( _value, MPS );

}

}

@Override

@AnyLogicInternalCodegenAPI

public void doStart() {

super.doStart();

yearlySaveAverage.start();

_plot2_autoUpdateEvent_xjal.start();

_plot3_autoUpdateEvent_xjal.start();

_plot4_autoUpdateEvent_xjal.start();

_plot_autoUpdateEvent_xjal.start();

_plot1_autoUpdateEvent_xjal.start();

pipe.start();

user.start();

agency.start();

}

/**

* Assigning initial values for plain variables<br>

* <em>This method isn't designed to be called by user and may be removed in future releases.</em>

*/

@AnyLogicInternalCodegenAPI

public void setupPlainVariables_xjal() {

setupPlainVariables_Main_xjal();

}

/**

* Assigning initial values for plain variables<br>

* <em>This method isn't designed to be called by user and may be removed in future releases.</em>

*/

@AnyLogicInternalCodegenAPI

private void setupPlainVariables_Main_xjal() {

CumulativeDemand =

0

;

CumulativeSupply =

0

;

}

// User API -----------------------------------------------------

@AnyLogicInternalCodegenAPI

static LinkToAgentAnimationSettings _connections_commonAnimationSettings_xjal = new LinkToAgentAnimationSettingsImpl( false, black, 1.0, LINE_STYLE_SOLID, ARROW_NONE, 0.0 );

public LinkToAgentCollection<Agent, Agent> connections = new LinkToAgentStandardImpl<Agent, Agent>(this, _connections_commonAnimationSettings_xjal);

@Override

public LinkToAgentCollection<? extends Agent, ? extends Agent> getLinkToAgentStandard_xjal() {

return connections;

}

@AnyLogicInternalCodegenAPI

public void drawLinksToAgents(boolean _underAgents_xjal, LinkToAgentAnimator _animator_xjal) {

super.drawLinksToAgents(_underAgents_xjal, _animator_xjal);

if ( _underAgents_xjal ) {

_animator_xjal.drawLink( this, connections, true, true );

}

}

public List<Object> getEmbeddedObjects() {

List<Object> list = super.getEmbeddedObjects();

if (list == null) {

list = new LinkedList<Object>();

}

list.add( pipe );

list.add( user );

list.add( agency );

return list;

}

public AgentList<? extends Main> getPopulation() {

return (AgentList<? extends Main>) super.getPopulation();

}

public List<? extends Main> agentsInRange( double distance ) {

return (List<? extends Main>) super.agentsInRange( distance );

}

@Override

@AnyLogicInternalCodegenAPI

public boolean isLoggingToDB(EventOriginator _e) {

if ( _e == _plot2_autoUpdateEvent_xjal ) return false;

if ( _e == _plot3_autoUpdateEvent_xjal ) return false;

if ( _e == _plot4_autoUpdateEvent_xjal ) return false;

if ( _e == _plot_autoUpdateEvent_xjal ) return false;

if ( _e == _plot1_autoUpdateEvent_xjal ) return false;

return super.isLoggingToDB( _e );

}

@AnyLogicInternalCodegenAPI

public void onDestroy() {

yearlySaveAverage.onDestroy();

_plot2_autoUpdateEvent_xjal.onDestroy();

_plot3_autoUpdateEvent_xjal.onDestroy();

_plot4_autoUpdateEvent_xjal.onDestroy();

_plot_autoUpdateEvent_xjal.onDestroy();

_plot1_autoUpdateEvent_xjal.onDestroy();

pipe.onDestroy();

user.onDestroy();

agency.onDestroy();

_plot2_expression0_dataSet_xjal.destroyUpdater_xjal();

_plot3_expression0_dataSet_xjal.destroyUpdater_xjal();

_plot4_expression0_dataSet_xjal.destroyUpdater_xjal();

_plot_expression0_dataSet_xjal.destroyUpdater_xjal();

_plot1_expression0_dataSet_xjal.destroyUpdater_xjal();

logToDB( _plot2_expression0_dataSet_xjal, "plot2 : Amount of Annual Leakage (Mgall)" );

logToDB( _plot3_expression0_dataSet_xjal, "plot3 : Number of Annual Breaks" );

logToDB( _plot4_expression0_dataSet_xjal, "plot4 : Amount of Annual Energy Loss (Mwh)" );

logToDB( _plot_expression0_dataSet_xjal, "plot : Service Reliability (%)" );

logToDB( _plot1_expression0_dataSet_xjal, "plot1 : Average Condition " );

super.onDestroy();

}

@AnyLogicInternalCodegenAPI

@Override

public void doFinish() {

pipe.doFinish();

super.doFinish();

user.doFinish();

super.doFinish();

agency.doFinish();

super.doFinish();

}

}
