# Supplementary figures and images for: Resilience as an emergent property of human-infrastructure dynamics: A multi-agent simulation model for characterizing regime shifts and tipping point behaviors in infrastructure systems
Source: PLoS One. 2018 Nov 21;13(11):e0207674. doi: 10.1371/journal.pone.0207674 (PMC6248985; doi:10.1371/journal.pone.0207674)

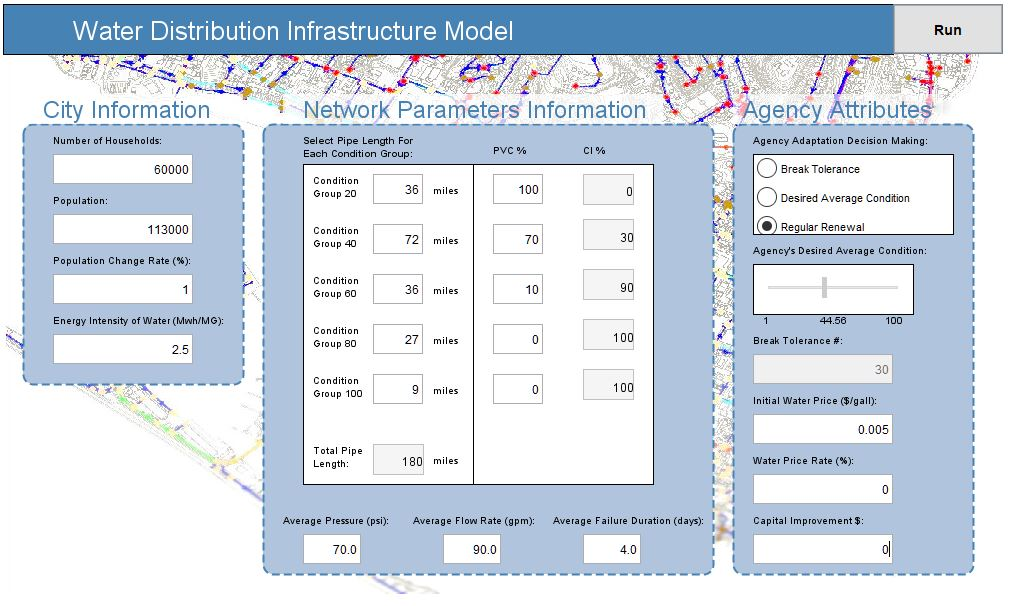

Supplement: S1 Fig — (TIF) [file pone.0207674.s002.tif]

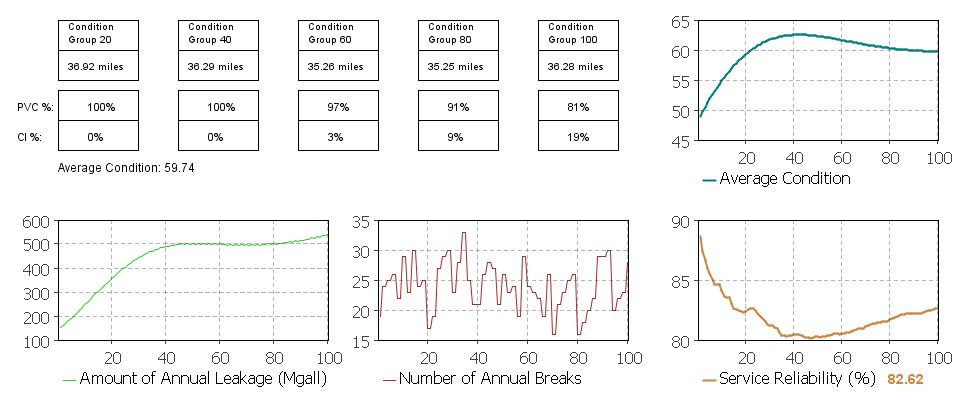

Supplement: S2 Fig — (TIF) [file pone.0207674.s003.tif]
